# Reappraisal of Europe’s most complete Early Cretaceous plesiosaurian: *Brancasaurus brancai* Wegner, 1914 from the “Wealden facies” of Germany

**DOI:** 10.7717/peerj.2813

**Published:** 2016-12-22

**Authors:** Sven Sachs, Jahn J. Hornung, Benjamin P. Kear

**Affiliations:** 1 Naturkundemuseum Bielefeld, Abteilung Geowissenschaften, Bielefeld, Germany; 2 Engelskirchen, Germany; 3 Hamburg, Germany; 4 Museum of Evolution, Uppsala Universitet, Uppsala, Sweden

**Keywords:** Leptocleididae, Elasmosauridae, *Gronausaurus wegneri*, Berriasian, Wealden facies, Bückeberg Group, Ontogenetic variability

## Abstract

The holotype of *Brancasaurus brancai* is one of the most historically famous and anatomically complete Early Cretaceous plesiosaurian fossils. It derived from the Gerdemann & Co. brickworks clay pit near Gronau (Westfalen) in North Rhine-Westphalia, northwestern Germany. Stratigraphically this locality formed part of the classic European “Wealden facies,” but is now more formally attributed to the upper-most strata of the Bückeberg Group (upper Berriasian). Since its initial description in 1914, the type skeleton of *B. brancai* has suffered damage both during, and after WWII. Sadly, these mishaps have resulted in the loss of substantial information, in particular many structures of the cranium and limb girdles, which are today only evidenced from published text and/or illustrations. This non-confirmable data has, however, proven crucial for determining the relationships of *B. brancai* within Plesiosauria: either as an early long-necked elasmosaurid, or a member of the controversial Early Cretaceous leptocleidid radiation. To evaluate these competing hypotheses and compile an updated osteological compendium, we undertook a comprehensive examination of the holotype as it is now preserved, and also assessed other Bückeberg Group plesiosaurian fossils to establish a morphological hypodigm. Phylogenetic simulations using the most species-rich datasets of Early Cretaceous plesiosaurians incorporating revised scores for *B. brancai*, together with a second recently named Bückeberg Group plesiosaurian *Gronausaurus wegneri* (Hampe, 2013), demonstrated that referral of these taxa to Leptocleididae was not unanimous, and that the topological stability of this clade is tenuous. In addition, the trait combinations manifested by *B. brancai* and *G. wegneri* were virtually identical. We therefore conclude that these monotypic individuals are ontogenetic morphs and *G. wegneri* is a junior synonym of *B. brancai*. Finally, anomalies detected in the diagnostic features for other “Wealden” plesiosaurians have prompted reconsiderations of interspecies homology versus intraspecific variability. We therefore propose that the still unresolved taxonomy of *B. brancai* should emphasize only those character states evident in the examinable fossil material, and specifically accommodate for growth-related modifications delimited via osteologically mature referred specimens.

## Introduction

**Figure 1:**
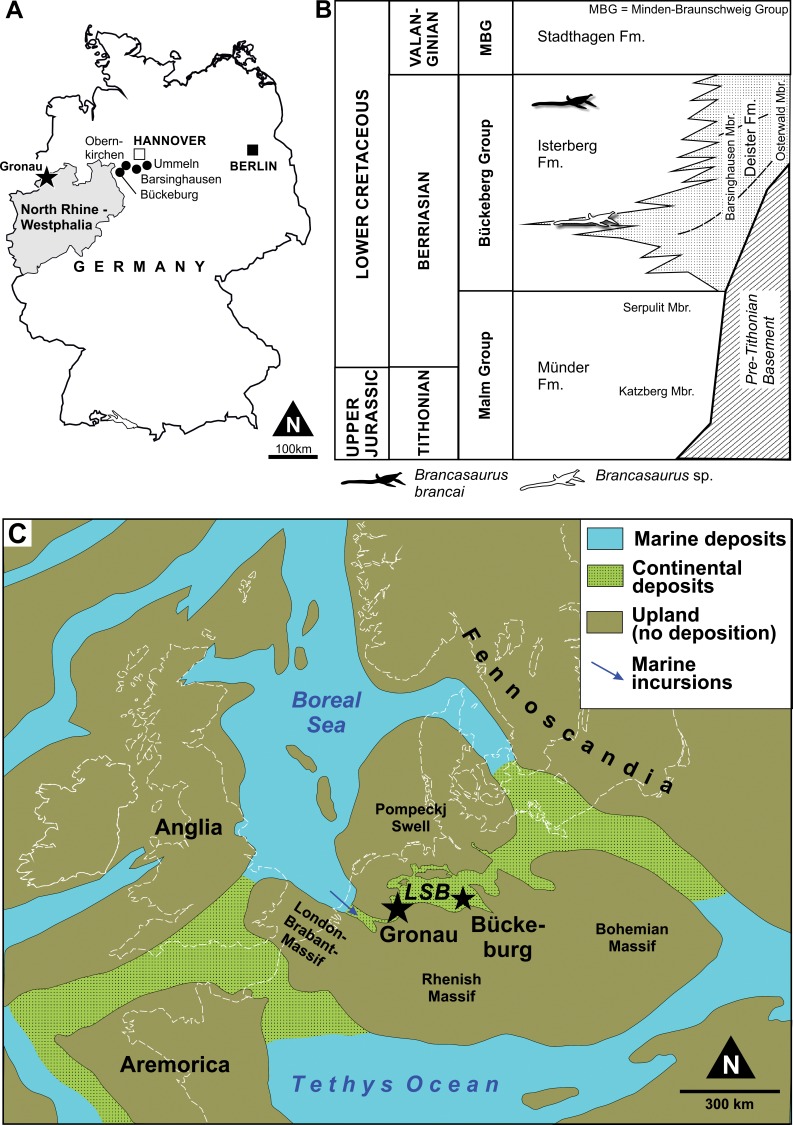
(A) Map of the type locality for *Brancasaurus brancai* at Gronau (Westfalen), Germany (asterisk), together with other pertinent sources of fossil material. (B) Lithostratigraphy of the lowermost Cretaceous in the central and southeastern Lower Saxony Basin (northwestern Germany) with the position of *B. brancai* and *B.* sp. in the upper Berriasian Bückeberg Group. (C) Palaeogeographical map of Central Europe during the Berriasian-Valanginian (after Mutterlose, 1997, modified), incorporating the Lower Saxony Basin (LSB) and the localities of Gronau and Bückeburg.

**Figure 2:**
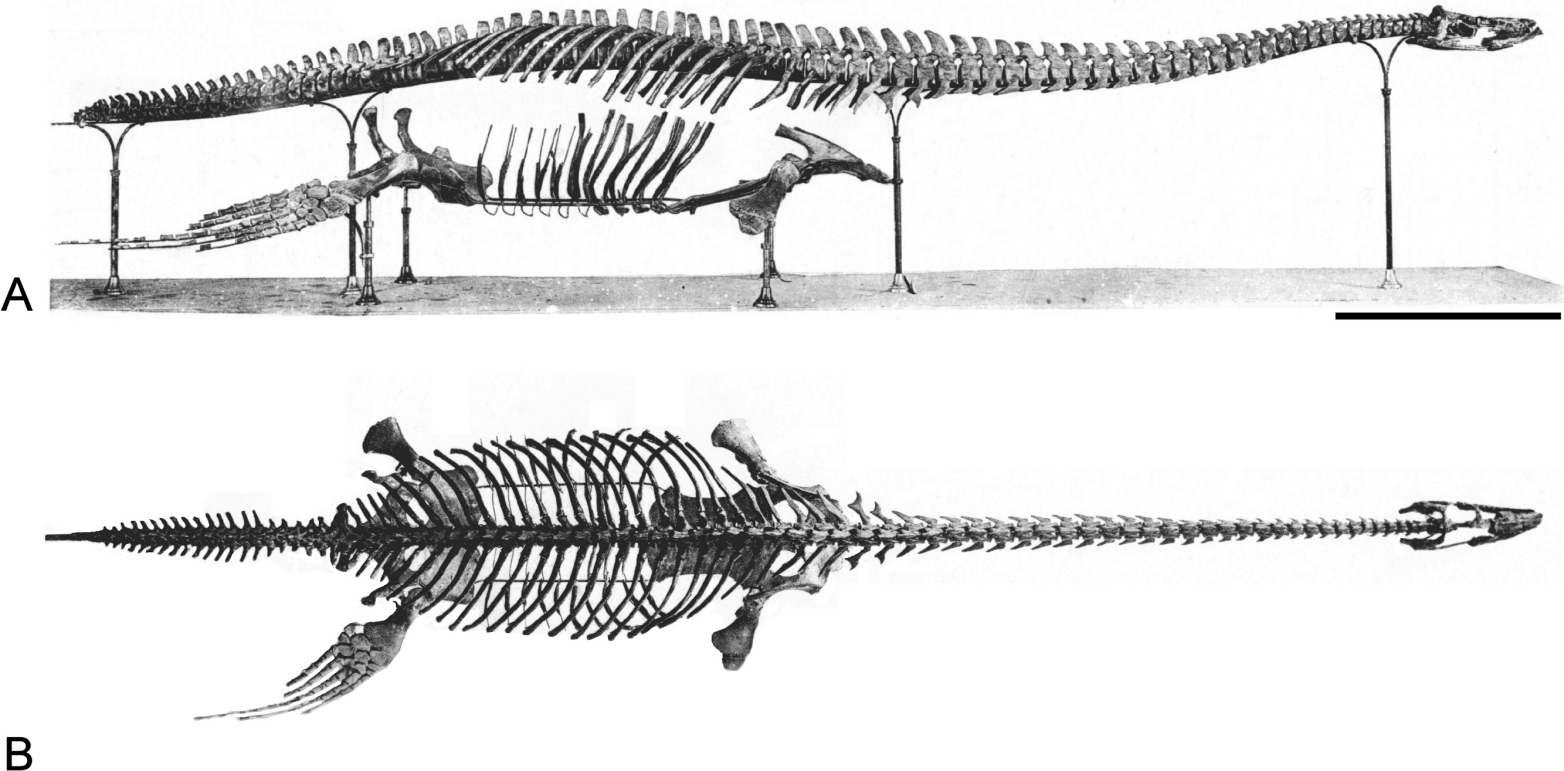
*Brancasaurus brancai*Wegner, 1914, Isterberg Formation, upper Berriasian of Gronau (Westfalen), North Rhine-Westphalia. GPMM A3.B4 (holotype), mounted skeleton as originally displayed at the Geological-Palaeontological Museum in Münster (from Wegner, 1914): (A) Lateral and (B) dorsal views. Scale bar = 500 mm.

*Brancasaurus brancai* is the most complete plesiosaurian taxon currently known from the Lower Cretaceous of Europe. The holotype skeleton (GPMM A3.B4) was discovered in July 1910 during commercial excavations at the Gerdemann & Co. brickworks clay-pit near Gronau (Westfalen) in North Rhine-Westphalia, northwestern Germany (Fig. 1A). Theodor Wegner (1880–1934), a palaeontologist at the University of Münster who initially inspected the specimen, reported that GPMM A3.B4 was exposed and broken up by pit workers using pickaxes (Wegner, 1914). Several days later he visited the site to collect the remaining elements, which were disarticulated, intermixed, and in some cases highly fragmented. Indeed, Wegner (1914) mentioned that only a few pectoral vertebrae (“Brustwirbel”) with appertaining ribs were left in association, and that the severely damaged right pubis had to be reassembled from 167 individual pieces. The pit owners, Mr. Gerdemann and Mr. Bertelsmann, eventually donated all of this material to the University of Münster, where it was painstakingly prepared and reconstructed under Wegner’s supervision (Fig. 2). Wegner finally published his formal description of the 3.26 m long skeleton in a festschrift commemorating the 70th birthday of Wilhelm von Branca (1844–1928), his former mentor, upon whom he bestowed the genus and species name *Brancasaurus brancai*.

Wegner (1914) provisionally assigned *B. brancai* to the ubiquitous long-necked plesiosauroid group Elasmosauridae, based on osteological comparisons and its compatibility with the family-level definition proposed by Andrews (1910: 77). However, he also explicitly stated that *B. brancai* differed from elasmosaurids in its small and narrow cranial proportions and relative length of the neck, development of the skull roof bones, dentition, and number of vertebrae along the column. Wegner further remarked on the unusual “triangular” shape of cervical neural spines (Wegner, 1914: 292). These observations initiated later classifications of *B. brancai* as a basal member (e.g., Welles, 1962; Brown, 1981; Brown, 1993; Carpenter, 1999; O’Keefe, 2001; O’Keefe, 2004a; Großmann, 2007), and clade specifier of Elasmosauridae (O’Keefe, 2001). Nevertheless, counter arguments were voiced by White (1940), who erected a separate family Brancasauridae, comprising *B. brancai*, *Seeleyosaurus guilelmiimperatoris* (Dames, 1895), and “*Thaumatosaurus*”—a redundant name occasionally applied to species of *Rhomaleosaurus* Seeley, 1874 and *Meyerasaurus* Smith & Vincent, 2010 (see Smith & Vincent, 2010). Sato (2002) also questioned the relationship of *B. brancai* with Elasmosauridae, and Ketchum & Benson (2010) derived an alternative placement within Leptocleididae, a clade revived by Druckenmiller & Russell (2008a) to encompass the iconic British Wealden taxon *Leptocleidus superstes* Andrews, 1922. The affinities of *B. brancai* with Leptocleididae have since been reiterated by derivative phylogenies, but were most explicitly espoused by Benson et al. (2013a) in a taxonomic reassessment of English Wealden plesiosaurian remains. Benson et al. (2013a) nested *B. brancai* within an exclusive Early Cretaceous lineage comprising the latest Valanginian *Leptocleidus capensis* (Andrews, 1911), Barremian *L. superstes*, late Barremian *Vectocleidus pastorum* Benson et al., 2013a early Aptian–early Albian *Umoonasaurus demoscyllus* Kear, Schroeder & Lee, 2006, and early Albian *Nichollssaura borealis* (Druckenmiller & Russell, 2008b). Benson & Druckenmiller (2014) also later incorporated the Valanginian *Hastanectes valdensis* (Lydekker, 1889), which Benson et al. (2013a) had placed in Pliosauridae. In addition, Benson et al. (2013a) listed various traits allying *B. brancai* with the more inclusive clade Leptocleidia: a reduced pair of rostral-most premaxillary alveoli; postorbital with a prolonged caudal process extending approximately one-third along the temporal fenestrae; a triangular fossa tapering proximally from the pineal foramen to the merge with the sagittal crest; the presence of a notch on the dorsal surface of the articular adjacent to the glenoid; cervical neural spines curved with the caudal-most bearing sub-oval, concave dorsal surfaces; dorsal neural spines sub-equal to the height of the centrum and bearing an alternating, asymmetrical morphology; a scapular shelf; and proximodistally elongate epipodials.

Recently, Hampe (2013) described a second articulated plesiosaurian skeleton GPMM A3.B2 (GMM A3.B2 *sensu* Hampe, 2013: 475) recovered from the Gerdemann & Co. clay-pit in 1912 (Wegner, 1914). This specimen derived from the uppermost horizon of the Bückeberg Group, about eight metres above the *B. brancai* type stratum. Siegfried (1961) provisionally allied GPMM A3.B2 with *B. brancai*; however, Hampe (2013) established it as the holotype of a new taxon, *Gronausaurus wegneri*, and placed it within Leptocleididae as the sister of *B. brancai*. Benson & Druckenmiller (2014), on the other hand, returned GPMM A3.B2 as a basal elasmosaurid using a non-exclusive trait combination: caudal cervical to dorsal neural spines with grooved caudal edge, dorsal neural spines with craniocaudally constricted base, presence of a ventral projection along the intercoracoid symphysis, and humerus to femur length ratio >1.1.

Because of these compounding uncertainties, we undertook a comprehensive survey of the German “Wealden facies” plesiosaurian material housed in museum and university collections across Germany and The Netherlands. Our objective was to evaluate the condition of these fossils first-hand, and clarify their stratigraphical context as well as critically appraise the character states used to advocate competing taxonomies. In addition, we compiled a detailed descriptive atlas of the *B. brancai* holotype, which is presented here as part of an updated comparative overview of Europe’s most complete Early Cretaceous plesiosaurian.

## Geological Context

### Lithostratigraphical setting

All of the remains attributable to *Brancasaurus brancai* originate from the Bückeberg Group (Fig. 1B). This unit reaches a thickness of more than 700 m at its depocenter and consists of mudstones, black-shales with subordinate sandstones, limestones and coals that accumulated within the epicontinental Lower Saxony Basin in northwestern Germany and the eastern Netherlands (Kemper, 1973). Historically, the Bückeberg Group was known as the “Deutscher Wealden” (German Wealden) because of its lithological, biotic and facies compatibility with the classical Valanginian–Aptian Wealden succession of southern England. The “Deutscher Wealden,” however, is stratigraphically older than its English equivalent, being mid to late Berriasian in age. Allen (1955) thus proposed an alternative nominal “Wealden facies”, which Casey et al. (1975) superseded with formal designation as the Bückeberg Formation (now Bückeberg Group, Erbacher et al., 2014a).

At Gronau, halotectonic uplift has locally exposed strata of the Isterberg Formation (sensu Erbacher et al., 2014b) within the Bückeberg Group, which are otherwise subsurface elsewhere in the region (Kemper, 1976; Kemper, 1992). Records from the Gerdemann & Co. clay-pit indicate that a 30 m thick succession of this unit was worked during the 19th and early 20th centuries (Hosius, 1893; Wegner, 1914). After abandonment in 1917, the pit was flooded with water, but pumped dry in 1959 before being filled again with soil. During this brief interval, Kemper (1961) produced a lithological log that correlated the outcrop with both the uppermost Isterberg Formation and lower Stadthagen Formation (= *Platylenticeras* Beds, Erbacher et al., 2014c.).

Lithostratigraphically, the Bückeberg Group overlies the marine to hyperhaline Münder Formation (Tithonian–lower Berriasian) and is succeeded by the marine Stadthagen Formation (Lower Valanginian: Elstner & Mutterlose, 1996; Mutterlose, 1997; Erbacher et al., 2014c.). Fossil and sedimentological distinctions have facilitated further subdivision of the Bückeberg Group into several formations and members, including the Isterberg Formation for the predominantly argillaceous basin deposits, passing margin-ward into regionally differentiated, coarser clastic units, including deltaic and fluviatile settings (Erbacher et al., 2014a). The latter include the Deister and Fuhse Formations (Erbacher et al., 2014d; Erbacher et al., 2014e.), which have also yielded some plesiosaurian material discussed herein. This more complex lithostratigaphical scheme was recently introduced to supersede the more simple subdivision in two members, the Obernkirchen and Osterwald Members, respectively, a nomenclature that has been established for almost 40 years (e.g., Kemper, 1976; Elstner & Mutterlose, 1996; Hornung, Böhme & Reich, 2012).

As a predominantly limnic-brackish sequence, the biostratigraphy of the Bückeberg Group is based on ostracods, charophytes, and palynomorphs (see Strauss et al., 1993; Elstner & Mutterlose, 1996; Pelzer, 1998; Mutterlose, 1997; Mutterlose, 2000; Hornung, Böhme & Reich, 2012; Hornung et al., 2012). Wolburg (1949) initially introduced a six-fold faunal zonation of “Wealden 1” through “Wealden 6,” which was then more finely split into 11 ostracod sub-zones (Wolburg, 1959). Until recently, the Berriasian/Valanginian boundary was assumed to be located within “Wealden 4” (e.g., Mutterlose, 2000). However, new results have pinpointed the Berriasian/Valanginian boundary at the top of the Bückeberg Group (Mutterlose, Bodin & Fähnrich, 2014). The upper Isterberg Formation at Gronau correlates to the “Wealden 5” and “Wealden 6” (Kemper, 1976), and therefore to the uppermost Berriasian.

### Palaeogeography and palaeoenvironment

The depositional setting of the Bückeberg Group (Fig. 1C) is thought to have been a large lake that received fluvial drainage from the surrounding uplands and sustained deltaic networks along its margins (Pelzer, 1998). At its western extremity, this lacustrine system communicated with the Boreal Sea via a narrow barrier gateway. This presumably functioned as an outflow for most of the lake’s life span; however, episodic transgressive phases, probably together with tectonic activity, enabled some marine ingression. Based on comparative microfaunal assemblage compositions, the accompanying propagation of brackish conditions seems to have followed a gradational decrease from West to East through “Wealden 1” to “Wealden 3,” but with more sustained marine influx in “Wealden 4,” and basin-wide brackish reinstatement associated with rapid transgression and lake expansion in “Wealden 5” and “Wealden 6” (Pelzer, 1998; Mutterlose & Bornemann, 2000; Berner, 2011).

The city of Gronau is situated in the western part of the Lower Saxony Basin, close to what was the Early Cretaceous Bückeberg Group lacustrine opening to the Boreal Sea (Wolburg, 1954; Kemper, 1976). The fossiliferous strata at this locality consist of predominantly C_org_-rich, calcareous claystones and shales with subordinate thin sideritic limestone coquinas, lumachelles, and bioclastic pack/floatstones (Wegner, 1914; Kemper, 1961; Kemper, 1973; Kemper, 1976; Kemper, 1992; Nyhuis & Herbig, 2009). The claystones and shales are largely devoid of benthic fauna and bioturbation, indicating deposition within a dysoxic hypolimnion (Berner, Kahl & Scheeder, 2010); this was linked to a basinal trough termed the Gronau Rinne by Wolburg (1954). However, interspersed low-diversity neomiodontid bivalve coquinas and intensely bioturbated horizons imply short phases of deep-water oxygenation. Bioclastic packstones and floatstones are concentrated near the top of the Isterberg Formation, and reflect a gradual transition into the fully marine Stadthagen Formation. Fossils from these sequences include shallow-water benthic invertebrates (Struckmann, 1880; Struckmann, 1891; Huckriede, 1967), fish remains (Nyhuis & Herbig, 2009), and semi-aquatic and terrestrial tetrapods (crocodilians and dinosaurs: Sachs & Hornung, 2013). These mostly represent allochthonous elements that were introduced via occasional basin-ward mass transport from density currents and debris flows that deposited debrites and tempestites from the oxygenated shallow water regions and epilimnion.

### Taphonomy

Wegner (1914) mentioned that GPMM A3.B4 was found 9–10 m below the top of the Isterberg Formation within a calcareous bituminous shale containing abundant neomiodontid bivalves. Conversely, the holotype of *Gronausaurus wegneri* (GPMM A3.B2) occurred approximately eight metres up-sequence within an unfossiliferous calcareous shale 1–2 m below the contact with the Stadthagen Formation (Wegner, 1914; Hampe, 2013). At least one more plesiosaurian skeleton has been reported from the Gerdemann & Co. clay-pit (Koken, 1905), suggesting that other articulated specimens might have been encountered but were probably destroyed during quarry operations (Wegner, 1914). The dysoxic hypoliminion implied by the shale-claystone sequences at Gronau should have favoured exceptional preservation of undisturbed remains (as evidenced by possible bromalites and soft-tissue remnants: Wegner, 1914). In contrast, the prevalence of benthic bivalves with GPMM A3.B4 infers occasional oxygenation of the sediment-water interface. Irrespectively, the Gronau plesiosaurians were probably parautochthonous, being transported into the hypolimnion via sinking through the water column shortly after death.

## Materials and Methods

We redescribe the holotype specimen of *Brancasaurus brancai* (GPMM A3.B4) and further referrable material, housed in the the Geomuseum der Universität Münster (GPMM) in Münster in Westfalen, Germany. Additional referrable and comparable specimens were studied in the collections of the Driland Museum (DLM) in Gronau (Westfalen), Germany, Geowissenschaftliches Zentrum der Georg-August-Universität Göttingen (GZG) in Göttingen, Germany, Museum für Naturkunde (MB) in Berlin, Germany, Naturmuseum Senckenberg (SMF) in Frankfurt am Main, Germany, Museum TwentseWelle (MTWE) in Enschede, The Netherlands and Natural History Museum (NHMUK) in London, UK. The cited material was studied and documented first-hand in conjunction with appropriate comparative literature where relevant. All studied material is stored in public collections and was accessed with formal permission from the responsible curating personel. Phylogenetic methods are explained below.

## Results

### Systematic palaeontology

**Table:** 

Sauropterygia Owen, 1860
Plesiosauria De Blainville, 1835
Plesiosauroidea Gray, 1825
*Brancasaurus* Wegner, 1914

*Type species: Brancasaurus brancai* Wegner, 1914

*Diagnosis:* As for the type and only species.

*Stratigraphical and geographical range:* Isterberg, Deister, and (?)Fuhse Formations, Bückeberg Group, upper Berriasian; Lower Saxony Basin, northwestern Germany.

*Brancasaurus brancai* Wegner, 1914

Our synonym list follows the recommended protocols of Richter (1948), Matthews (1973) and Becker (2001), who prescribed inclusion of both total reference data arising from the species, together with works that directly contribute either morphological information or interpretations (see Matthews, 1973: 717). In addition to the definition of Matthews (1973) we added all references known to us to synthesize recognition of the taxon in both scientific and popular scientific works. An abbreviation system was also advocated by Matthews (1973) and Becker (2001) to indicate qualifying comments: “year of publication in roman” = work contributes to knowledge of the species; “*year of publication in italics*” = work mentions species without description or illustration; “v” = *vidimus*—referral confirmed via inspection of deposited specimen/s; “v*” = referral confirmed via inspection of type specimen/s; “v?” = condition of deposited specimen/s prevents clear decision; “v•” = we accept reponsibility and have basis for attaching this reference to the discussed species; “no sign in front of year of publication” = we have no basis for accepting reponsibility but have no cause to doubt allocation.

**Table:** 

v ?	1887	*Plesiosaurus limnophilus* n. sp.—Koken: 417ff., pl. IX, Figs. 5A–C.
?	1905	*Plesiosaurus Degenhardti* Koken—Koken: 682ff., Figs. 1–3.
?	1905	*Plesiosaurus limnophilus* Koken—Koken: 687f., Figs. 4 and 5.
?	1905	*Plesiosaurus valdensis* Lydekker—Koken: 688ff., Fig. 6.
?	1905	*Plesiosaurus Kanzleri* n. sp.—Koken: 691ff., Fig. 7.
v*	1914	*Brancasaurus Brancai* n. gen n. sp—Wegner: 235ff., Figs. 1–10, pl. V–IX.
v•	1922	*Brancasaurus brancai* Wegner—Andrews: 287ff.
v•	1926	*Brancasaurus Brancai* Wegner—Wegner: 228ff, Fig. 142.
v•	1928	*Plesiosaurus* sp.—Edinger: 380, Fig. 1.
v•	*1928*	*Brancasaurus brancai*—Janensch: 94.
v•	1930	*Brancasaurus Brancai* Wegner—Edinger: 135f.
v•	1934	*Brancasaurus brancai* Wegner, 1914—Kuhn: 94.
v•	1935	*Brancasaurus brancai*—Stromer: 8ff.
v•	1940	*Brancasaurus brancai* Wegner—White: 463, Figs. 9C and 13.
v•	1943	*Brancasaurus*—Welles: 198, Fig. 37.
v•	1949	*Brancasaurus brancai*—Colbert: 8ff., Table 1.
v•	1956	*Brancasaurus* Wegner—Von Huene: 399, Fig. 443.
v•	*1957*	*Brancasaurus*—Krul: 139.
v•	1961	*Brancasaurus brancai*—Siegfried: 176ff., Figs. 1–3.
v•	1962	*Brancasaurus brancai* Wegner—Welles: 41ff., Fig. 8, Table 4.
?	1962	*Plesiosaurus kanzleri* Koken—Welles: 45.
v•	1963	*Brancasaurus brancai*—Persson: 6ff.
?	*1963*	“*Plesiosaurus” limnophilus* Koken, 1887—Persson: 27
?	*1963*	“*Plesiosaurus” kanzleri* Koken, 1905—Persson: 27
v•	1967	*Brancasaurus*—Kuhn: 67, Fig. 27.4.
v•	1968	*Brancasaurus*—Müller, Figs. 193 and 197.
v•	*1968*	*Brancasaurus brancai* Wegner—Thiermann: 44
v•	*1972*	*Brancasaurus brancai* Wegner, 1914—Kuhn: 2.
v•	*1975*	*Brancasaurus*—Brown: 11ff.
v•	1976	*Brancasaurus brancai* Wegner—Kemper, Fig. 7.
v•	1979	*Brancasaurus*—Hopson: 121f.
v	*1980*	*Brancasaurus*—Dong: 196
v•	*1981*	*Brancasaurus brancai*—Brown pp. 333ff.
v•	1982	*Brancasaurus brancai* Wegner—Dickel: 32ff., Figs. 1–8.
v•	1982	*Brancasaurus brancai* Wegner—Anonymus: 138f., 2 Figs.
v•	1985	*Plesiosaurus brancai*—Corcos: 21ff., Fig. 2.
v•	1986	*Brancasaurus brancai*—Probst: 186, 1 Fig.
v•	1992	*Brancasaurus brancai* Wegner—Kemper, pl. 1, Fig. 1.
v•	1992	*Brancasaurus brancai* Wegner—Schleicher: 118ff., 2 Fig.
v•	1993	*Brancasaurus*—Brown: 13f.
v•	1993	*Brancasaurus brancai*—Bakker: 657ff., Figs. 11E and 15.
v•	1995	*Brancasaurus brancai* Wegner—Schleicher: 111ff., Figs. 1–7.
v•	*1996*	*Brancasaurus brancai*—Sachs: 243.
v•	1997	*Brancasaurus brancai*—Carpenter: 206ff., Fig. 8A
v•	1997	*Brancasaurus brancai*—Sachs (a): 22ff., Fig. 1, Table 1.
v•	*1997*	*Brancasaurus brancai*—Sachs (b): 56.
v•	1999	*Brancasaurus brancai*—Carpenter: 150ff., Table 2, Fig. 15.
v•	1999	*Brancasaurus*—Bardet, Godefroit & Sciau: 946.
v•	2000	*Brancasaurus brancai*—Sachs: 32.
v•	2001	*Brancasaurus brancai* Wegner, 1914—O’Keefe: 14ff., Fig. 20, Table 1, Appendix 2.
v•	2002	*Brancasaurus*—O’Keefe, Fig. 2.
v•	2002	*Brancasaurus brancai*—Sato: 92ff., Figs. 4.11–4.22, Table 4.1, Appendix F.
v•	2003	*Brancasaurus*—O’Keefe & Wahl: 57, Fig. 7, Appendix 2.
v•	2003	*Brancasaurus brancai*—Smith: 8ff., Figs. 2.2, 2.10, 2.11, 4.5–4.7 and 4.10, Appendix 3, 5
v•	*2003*	*Brancasaurus* Wegner, 1914—Lazo & Cichowolski: 784.
v•	*2003*	*Brancasaurus*—Ellis: 169.
v•	2004	*Brancasaurus*—O’Keefe (a), Fig. 8, Appendix.
v•	2004	*Brancasaurus*—O’Keefe (b): 336, Fig. 11.
v•	2004	*Brancasaurus*—Sachs: 217ff.
v•	2004	*Mosasaurus*—Polenz & Spaeth: 138, 1 Fig.
v•	2005	*Brancasaurus*—Kear (a): 796ff., Appendix 2.
v•	2005	*Brancasaurus*—O’Keefe & Carrano, Figs. 2 and 4, Appendix 2.
v•	2005	*Brancasaurus*—Sachs (a): 434ff., Fig. 8, Table 1.
v•	2005	*Brancasaurus brancai*—Sachs (b): 104ff.
v•	*2005*	*Brancasaurus brancai* (Wegner, 1914)—Hampe: 49
v•	2006	*Brancasaurus brancai*—Druckenmiller: 131ff., Fig. 4.41.
v•	2006	*Brancasaurus brancai*Wegner, 1914—Druckenmiller & Russell: 184ff.
v•	2006	*Brancasaurus*—Großmann: 54ff., Fig. 4.1, Tables 4.1, 6.1.
v•	2006	*Brancasaurus brancai*—O’Keefe & Hiller: 207ff., Fig. 4, Table 3.
v•	2006	*Brancasaurus*—Kear, Schroeder & Lee, Supporting Material.
v•	2007	*Brancasaurus*—Gasparini, Fig. 12.2.
v•	2007	*Brancasaurus*—Großmann: 553ff., Fig. 8, matrix.
v•	2007	*Brancasaurus*—Schumacher, Fig. 8.
v•	*2007*	*Brancasaurus brancai*—Smith, Apendix 1
v•	2008	*Brancasaurus*—Druckenmiller & Russell (b): 22ff.
v•	2009	*Brancasaurus*—O’Keefe & Street: 53, Fig. 8, Apendix 2.
v•	*2009*	*Brancasaurus brancai*—Nyhuis & Herbig: 85.
v•	2009	*Brancasaurus*—McHenry: 120.
v•	2010	*Brancasaurus*—Benson et al., Apendix S1.
v•	2010	*Brancasaurus brancai*—Ketchum & Benson: 366ff., Figs. 2–8, Table 3.
v•	2010	*Brancasaurus brancai*—Carpenter et al.: 1ff., Fig. 2.
v•	*2011*	*Brancasaurus*—Benson et al.: 271.
v•	2011	*Brancasaurus brancai*—Kear & Barrett: 664ff.
v•	2011	*Brancasaurus brancai*—Ketchum & Benson, Fig. 16, Appendix.
v•	2011	*Brancasaurus*—Sato et al.: 315ff.
v•	2011	*Brancasaurus*—Vincent et al.: 1064ff.
v•	*2011*	*Brancasaurus brancai*—Sachs: 12
v•	2011	*Brancasaurus brancai*—Benson et al. (a), Appendix.
v•	2011	*Brancasaurus brancai*—Schwermann & Sander, Fig. 26.
v•	2012	*Brancasaurus brancai*—Druckenmiller & Knutsen: 282, Figs. 1–2.
v•	2012	*Brancasaurus brancai*—Evans: 2.80ff., Appendix II, IV.
v•	2012	*Brancasaurus brancai*—Kubo, Mitchell & Henderson: 568, Fig. 10.
v•	2012	*Plesiosaurus*—Oftring: 088, 1 Fig.
v•	2012	*Brancasaurus brancai*—Otero, Soto-Acuña & Rubilar-Rogers, Fig. 11.
v•	2012	*Brancasaurus*—Smith, Araújo & Mateus: 258, Fig. 4, Appendix 1
v•	2012	*Brancasaurus brancai*—Böhme et al.: 157, Fig. 7A.
v•	2012	*Brancasaurus brancai* (Wegner, 1914)—Karl, Nyhuis & Schleicher: 32ff.
v•	2013	*Brancasaurus*—Benson et al.: 234ff., Figs. 4–5, Appendix.
v•	2013	*Gronausaurus wegneri*, n. gen. n. sp.—Hampe: 475ff., Figs. 2–9, Table 1, character matrix.
v•	2013	*Brancasaurus brancai*—Hampe: 474ff., Figs. 2–9, Table 1, character matrix.
v•	2013	*Brancasaurus*—Brown, Vincent & Bardet: 544ff.
v•	2013	*Brancasaurus brancai* Wegner, 1914—Hornung, Sachs & Kear: 75
v•	2013	*Brancasaurus* (Wegner, 1914)—Smith: 151.
v•	2013	*Brancasaurus brancai*—Benson et al. (b): 29, Fig. 23.
v•	2013	*Brancasaurus brancai*—O’Gorman: 224f., Fig. 7.1, Apéndice II.
v•	2014	*Brancasaurus brancai*—Benson & Druckenmiller: 6ff., Figs. 2–3, character matrix, Appendix 1–2.
v•	2014	*Brancasaurus brancai*—Otero et al., Fig. 17, Appendix 1.
v•	2014	*Brancasaurus brancai*—Otero et al.: 325.
v•	*2014*	*Brancasaurus brancai*—Sachs & Hornung: 30.
v•	*2014*	*Brancasaurus brancai* Wegner, 1914—Sachs, Schubert & Kear: 30.
v•	*2015*	*Brancasaurus brancai* Wegner, 1914—Sachs & Kear: 694f.
v•	2015	*Brancasaurus brancai*—O’Gorman et al., Fig. 14, dataset.
v•	*2015*	*Brancasaurus brancai* (Wegner, 1914)—O’Gorman et al.: 381ff.
v•	2015	*Brancasaurus brancai*—Parrilla-Bel & Canudo: 221ff, Fig. 5, Table 1
v•	*2015*	*Gronausaurus wegneri* (Hampe, 2013)—Parrilla-Bel & Canudo: 216ff.
v•	2016	*Brancasaurus brancai*—Schumacher & Martin, Fig. 16.
v•	2016	*Brancasaurus brancai*—Schumacher & Martin, Fig. 16.
v•	2016	*Brancasaurus brancai*—Otero: 36ff., Fig. 13, Table 6.
v•	2016	*Gronausaurus wegneri*—Otero: 36.
v•	2016	*Brancasaurus brancai*—Sachs et al. (a): 36.
v•	2016	*Gronausaurus wegneri*—Sachs et al. (a): 36.

**Figure 3:**
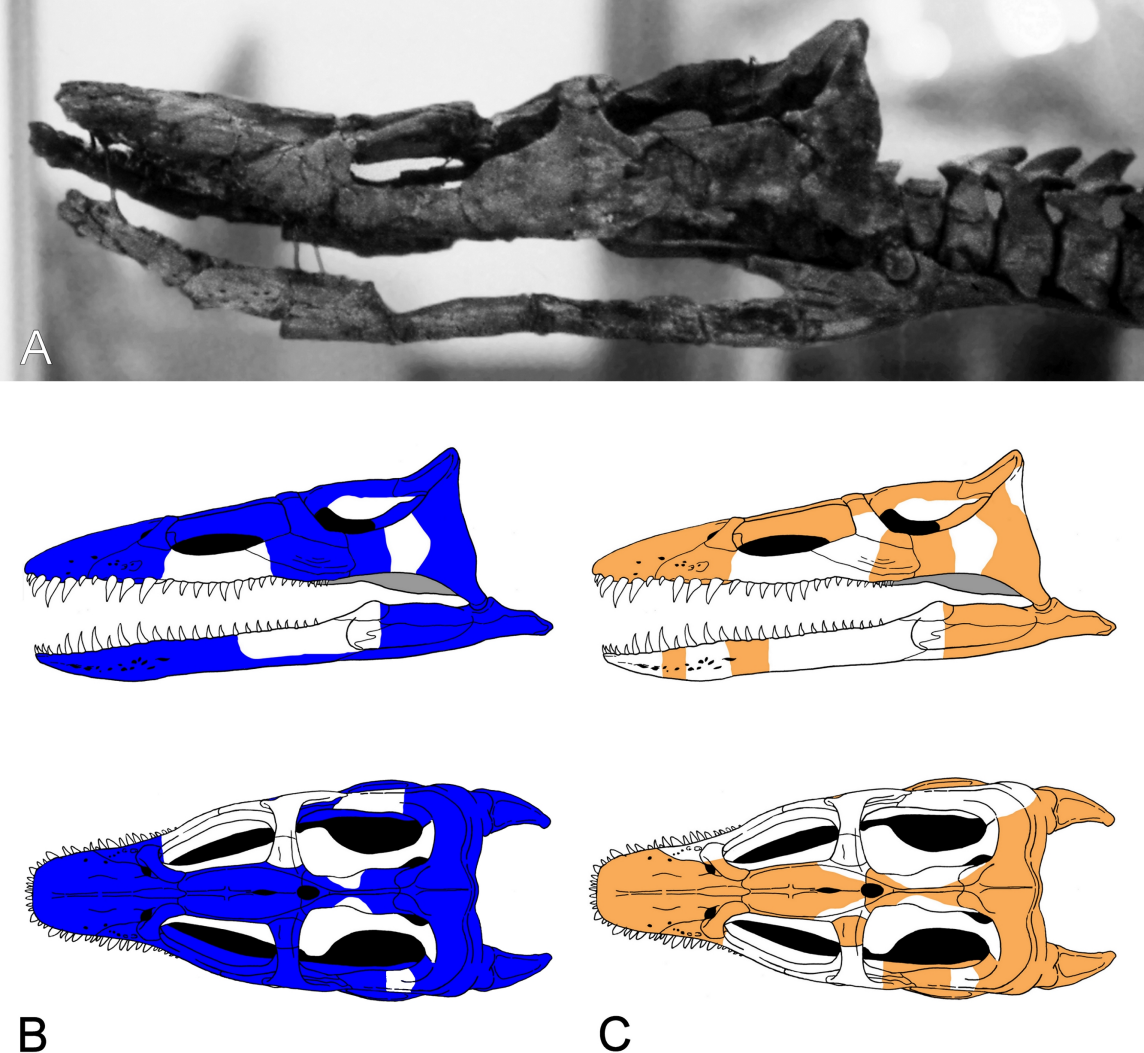
*Brancasaurus brancai*, GPMM A3.B4 (holotype). (A) Cranium and mandible in lateral view, showing its condition in the late 1980s. (B) Reconstructed cranium and mandible in lateral and dorsal views; recovered components identified by Wegner (1914) (blue); (C) components restored in the present mount (orange).

*Holotype:* Elements listed by Wegner (1914) but now lost (see Fig. 3) are marked with †. We also define “partial” as less than 50% intact. GPMM A3.B4, almost complete skeleton, includes an incomplete skull with both premaxillae, partial left and † right maxilla, partial prefrontals (originally complete), both frontals, partial left jugal, partial left postorbital (originally complete), left postfrontal, both parietals, partial squamosals, both quadrates, both vomera, † partial palatines, † partial pterygoids, basioccipital, basisphenoid, parasphenoid, partial left exoccipital-opisthotic (originally complete), † supraoccipital, partial prootics (originally complete), partial dentaries, both surangulars, both angulars, left articular, † teeth, 37 cervical vertebrae (including the atlas-axis complex), partial cervical ribs, three pectoral vertebrae, 19 dorsal vertebrae, several complete and partial ribs, 22 gastralia (originally 37), three sacral vertebrae with sacral ribs, 22 caudal vertebrae (originally 25), partial caudal ribs, partial interclavicle, partial clavicles, partial scapulae, partial coracoids, both humeri, † right radius, both pubes, both ischia, both ilia, right and † left femur, † right tibia, † right fibula, 14 mesopodials and 14 phalanges. SMF R4076 wax endoneurocranial cast of GPMM A3.B4.

*Referred specimens:* GPMM A3.B2 (holotype of *Gronausaurus wegneri*): three teeth, basioccipital, basisphenoid, partial parasphenoid, fragmentary maxillary and/or dentary components, parietal, squamosal arch, vomers, pterygoids, six caudad cervical vertebrae, three pectoral vertebrae, 17 dorsal vertebrae, rib fragments, four sacral vertebrae, 22 caudal vertebrae, partial coracoids, partial left scapula, both pubes, left ischium, left ilium, partial right ilium, right humerus, partial left humerus, one radius, one ulna, both femora, one fibula, four mesopodials, two metapodials, 12 phalanges.

Numerous isolated propodials and vertebrae from the Gerdemann & Co. clay-pit are housed in the collections of the GPMM, MTWE, DLM and GZG. These are morphologically indistinguishable from the corresponding elements of *B. brancai*. Koken (1905) also referred vertebrae from the same locality to *Plesiosaurus degenhardti* Koken, 1887, *Plesiosaurus limnophilus* Koken, 1887, *Plesiosaurus valdensis* (Lydekker, 1889) (= *Cimoliosaurus valdensis* (Lydekker, 1889) = *Hastanectes valdensis* (Lydekker, 1889) *sensu* Benson et al., 2013a), and *Plesiosaurus kanzleri* Koken, 1905. *P. limnophilus* and *P. kanzleri* were erected by Koken (1887) and Koken (1905) based upon isolated, undiagnostic and partly lost material. These specimens have been considered *nomina dubia* (by Welles, 1962) and, although similar to *B. brancai* (compare Koken, 1887, pl. 9, Figs. 5A–5C and Koken, 1905, Fig. 7), show no diagnostic character combinations which would allow an unambigious referral.Therefore both are not available as senior synonyms for *B. brancai*.

*Type stratum and locality:* Isterberg Formation (“Wealden 6,” *Pachycytheridea trapezoidalis* ostracod zone, Mutterlose, 2000), Bückeberg Group, uppermost Berriasian, Lower Cretaceous; Gerdemann & Co brick-works clay-pit, northeast of Gronau (Westfalen), North Rhine-Westphalia, northwestern Germany (Wegner, 1914; Kemper, 1976).

*Stratigraphical and geographical range:* Diagnostic remains of *Brancasaurus brancai* are thus far restricted to the type stratum and locality. Compatible isolated elements also occur in roughly coeval strata of Barsinghausen (upper Isterberg Formation), as well as Ummeln (Fuhse Formation) in Lower Saxony. These localities are located within the central and eastern areas of the Lower Saxony Basin, suggesting that remains referrable to the taxon could potentially be found basin wide.

*Revised diagnosis*: Plesiosaurian distinguished by a unique character state combination: palatal surface of premaxillae with prominent rostrally converging ridges adjacent to the vomers; maxilla-squamosal contact short; frontals fused dorsally and enclosing a mid-line foramen; frontals rectangular in outline with a conspicuously concave dorsal surface and ventrally confluent lateral sides (imparting a triangular cross-section); prominent parietal table extending to pineal foramen; inter-squamosal suture abruptly raised; deep notch in caudad edge of the mandibular glenoid fossa; exoccipital-opisthotic perforated by three foramina medially (rostralmost foramen slit-like) and two foramina laterally; prominent oval excavation on the lateral surface of mandible close to the glenoid fossa; cervical and pectoral centra with deeply excavated notochordal pits; combined width of cervical pre- and postzygapophyses narrower than the width of the centrum; distinctly triangular (caudally arcuate) neural spines in the craniad and middle cervicals; transverse processes of dorsal vertebrae with subdiapophyseal fossae; scapula bears a prominent lateral shelf; coracoid with pronounced ventral process at the inter-coracoid symphysis; medial pubis-ischium contact forms a pelvic bar; pubis with craniolateral cornu; propodials bear facets for supernumerary ossifications.

*Phylogenetic Definition:* Character (number [state change]) distributions derive from our re-analysis of the Benson et al. (2013a) and Benson & Druckenmiller (2014) phylogenetic datasets. Because these topologies are labile and conflicting, we also herein restrict our usage of plesiosaurian higher-level nomenclature to family-level clade designations. Benson et al. (2013a): *Brancasaurus brancai* can be distinguished from all plesiosaurians outside of Cryptoclididae + Leptocleididae + Polycotylidae by its possession of shallowly concave cervical vertebrae with deeply excavated notochordal pits (47 [1 ≥ 0/1]; this character is polymorphic and probably ontogenetically influenced in both the holotype GPMM A3.B4, and referred specimen GPMM A3.B2), and the presence of a craniolateral cornu on the pubis (174 [0 ≥ 1). *Brancasaurus brancai* is further excluded from Cryptoclididae by its maxilla-squamosal contact (16 [0 ≥ 1]), presence of a deep notch in the posterior border of the glenoid (104 [0 ≥ 1]) and mandible with a prominent longitudinal trough on its caudolateral surface (180 [0 ≥ 1]). *Brancasaurus brancai* differs from polycotylids in its possession of a lateral scapular shelf (146 [0 ≥ 1]) and caudodorsally curving cervical neural spines (212 [1 ≥ 0/1]; but these become straight and sheet-like in the more caudal cervicals). Finally, *B. brancai* specifically contrasts with the leptocleidids *Nichollssaura borealis* + *Umoonasaurus demoscyllus* + *Vectocleidus pastorum* + *Leptocleidus capensis* + *L. superstes* in its greater combined number of cervical and pectoral vertebrae (118 [G ≥ B]; unknown in GPMM A3.B2), dorsal neural spines being conspicuously taller than the accompanying centra (137 [1 ≥ 0]; polymorphic in GPMM A3.B4), and slightly more equal humerus to femur length ratio (153 [C ≥ B]). Benson & Druckenmiller (2014): *B. brancai* can be discriminated from plesiosaurians other than Leptocleididae + Polycotylidae by its maxilla-squamosal contact (26 [0 ≥ 1]), abruptly raised inter-squamosal suture (48 [0 ≥ 2]), prominent trough on the lateral surface of the mandible adjacent to the glenoid (121 [0 ≥ 1]), deep notch in the caudal border of the glenoid (130 [0 ≥ 1]), and proportional width of the cervical centra ranging up to 1.2 times their height (173 [1 ≥ 0/1]; polymorphic in GPMM A3.B4 but “0” in GPMM A3.B2). It also uniquely differentiates in its possession of a ventral process on the intercoracoid symphysis (215 [0 ≥ 1]) and the length/width ratio of the ischium being <0.9 (231 [1 ≥ 0]). Furthermore, *B. brancai* lacks planar cervical zygapophyses (169 [1 ≥ 0]), caudal ribs positioned at the mid-height of the centrum (188 [2 ≥ 1/2]; polymorphic in GPMM A3.B4, “2” in GPMM A3.B2), and sigmoid ilial shaft (221 [1 ≥ 2]) that otherwise characterise Leptocleididae + Polycotylidae. The absence of a prominent condylar groove on the basioccipital (65 [0 ≥ 2]; potentially ontogenetic) and caudomedial inflection of the retroarticular process (123 [0 ≥ 1]; “?” in GPMM A3.B2) additionally excludes *B. brancai* from Leptocleididae.

*Brancasaurus* sp.

*Material:* GZG.BA.0079, associated pubes, ischium, dorsal neurapophyses, partial centrum, fragmentary dorsal rib of a subadult individual.

*Stratigraphic and geographic range:* Obernkirchen Sandstone (“Wealden 3,” *Cypridea alta formosa* ostracod subzone, Elstner & Mutterlose, 1996), Barsinghausen Member, Deister Formation (Erbacher et al., 2014d), Bückeberg Group, upper Berriasian, Lower Cretaceous, Bückeburg area, Lower Saxony, northwestern Germany.

*Remarks:* This material shows a combination of characters similar to *B. brancai* (see discussion below).

### Descriptive reassessment of the holotype

Wegner (1914: 240) reported that the vertebral column of the *Brancasaurus brancai* holotype specimen (GPMM A3.B4, Fig. 2) was articulated prior to excavation, except for some slight displacement of the caudal series. His reassembly was therefore based upon outline impressions preserved in the surrounding sedimentary matrix. The limb elements were otherwise completely disassociated, and the skull was transversely fractured and suffered damage to the ventral side. Wegner’s (1914) restoration of GPMM A3.B4 was intended for a display mount with the skeleton embedded in plaster on its right-hand side. During preparation the recovered bones were reassembled and therefore coated with shellac to enhance their appearance. During WWII the specimen was evacuated to a humid storage facility, which propagated dissolution of the shellac and disaggregation of many elements especially parts of the skull. More disastrous, however, was an accidental fall of the skull from a suspended steel armature during renovation of the exhibition in 2002 (M Bertling, pers. comm., 2012). This resulted in shattering of the skull and complete destruction of parts of the basicranium and palate. Today, these missing components are evidenced only from Wegner’s (1914) published drawings (see Fig. 3).

Our first-hand inspections of GPMM A3.B4 were undertaken periodically from 2012 to 2015, at which time the fossil was mostly off-display and held in a secure storage facility. The only exception was during exhibition of the skull at MTWE in 2012. Our virtually unrestricted access permitted detailed documentation of key diagnostic structures. Furthermore, we were able to confirm the osteologically immature state of the specimen (see below), as well as the loss of a substantial amount of bone material incurred via damage to the skull and postcranium. In addition, some potentially referrable skeletal elements were located in the collection of the University of Münster. These are discussed where relevant but with the caveat that they cannot be definitively associated with GPMM A3.B4. Finally, Edinger (1928) figured a wax endoneurocranial cast labelled *Plesiosaurus* sp.. Edinger (1930), Hopson (1979) and Carpenter (1997) later identified this as a model of GPMM A3.B4 that had been assembled from impressions of various basicranial elements. Edinger (1930) mentioned that three copies of this cast were manufactured by Ms. Erfurt of Wiesbaden, with one from the collection of Otto Jaekel in Greifswald eventually deposited in the SMF. We describe it here as part of the total reference material pertaining to *B. brancai*.

#### Ontogenetic stage of GPMM A3.B4

The unfused neurocentral sutures in all vertebrae indicate that GPMM A3.B4 was an immature individual (sensu Brown, 1981). However, the propodials have well defined epipodial facets and cornua are present on the pubes. This demonstrates that the specimen was not in an early juvenile stage (sensu Brown, 1981). Indeed, the substantial maximum length of the articulate skeleton (3.26 m as measured by Wegner, 1914) suggests that GPMM A3.B4 was likely a subadult individual.

**Table 1:** Cranial measurements (mm) of *Brancasaurus brancai* (GPMM A3.B4).

Cranium—complete length rostrocaudally along midline (pmx-sq)	237
Premaxillae—rostrocaudal length (as preserved)	99
Maxilla—transverse diameter of largest alveolus	9
Frontals—transverse width	20
Parietals—rostrocaudal length (as preserved)	67
Basioccipital—maximum transverse width (as preserved)	29
Basioccipital—transverse width of condylus occipitalis	15
Basioccipital—dorsoventral height of condylus occipitalis	15
Exoccipital—dorsoventral heigth (as preserved)	21
Exoccipital—rostrocaudal length dorsally	18
Parasphenoid—transverse width of base	18
Basisphenoid—maximum transverse width (as preserved)	24
Quadrate—transverse width ventrally	24
Vomer—rostrocaudal length (left side)	61
Dentary—dorsoventral height midlength (as preserved)	11
Dentary—transverse width midlength	14
Surangular-angular complex—dorsoventral height at preserved most rostal section	27
Articular—transverse width of glenoid fossa	24
Articular—rostrocaudal length of retroarticular process	42

#### Cranium

**Figure 4:**
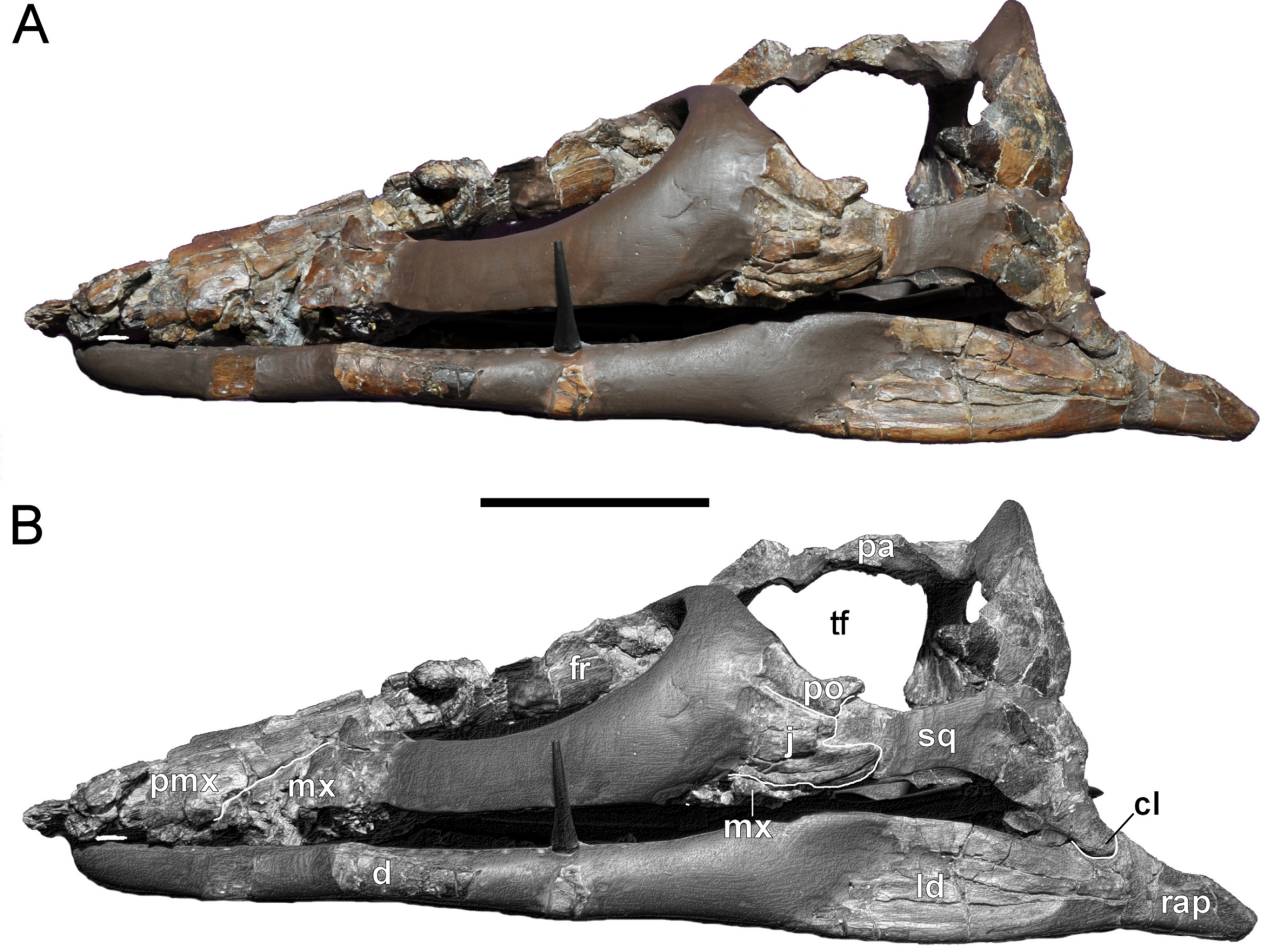
*Brancasaurus brancai*, GPMM A3.B4 (holotype). (A, B) Cranium and mandible in lateral view. Scale bar = 50 mm. Abbreviations: cl, condylus lateralis of quadrate; d, dentary; fr, frontal; j, jugal; ld, lateral depression; mx, maxilla; pa, parietal; pmx, premaxilla; po, postorbital; rap, retroarticular process; sq, squamosal; tf, temporal fenestra.

**Figure 5:**
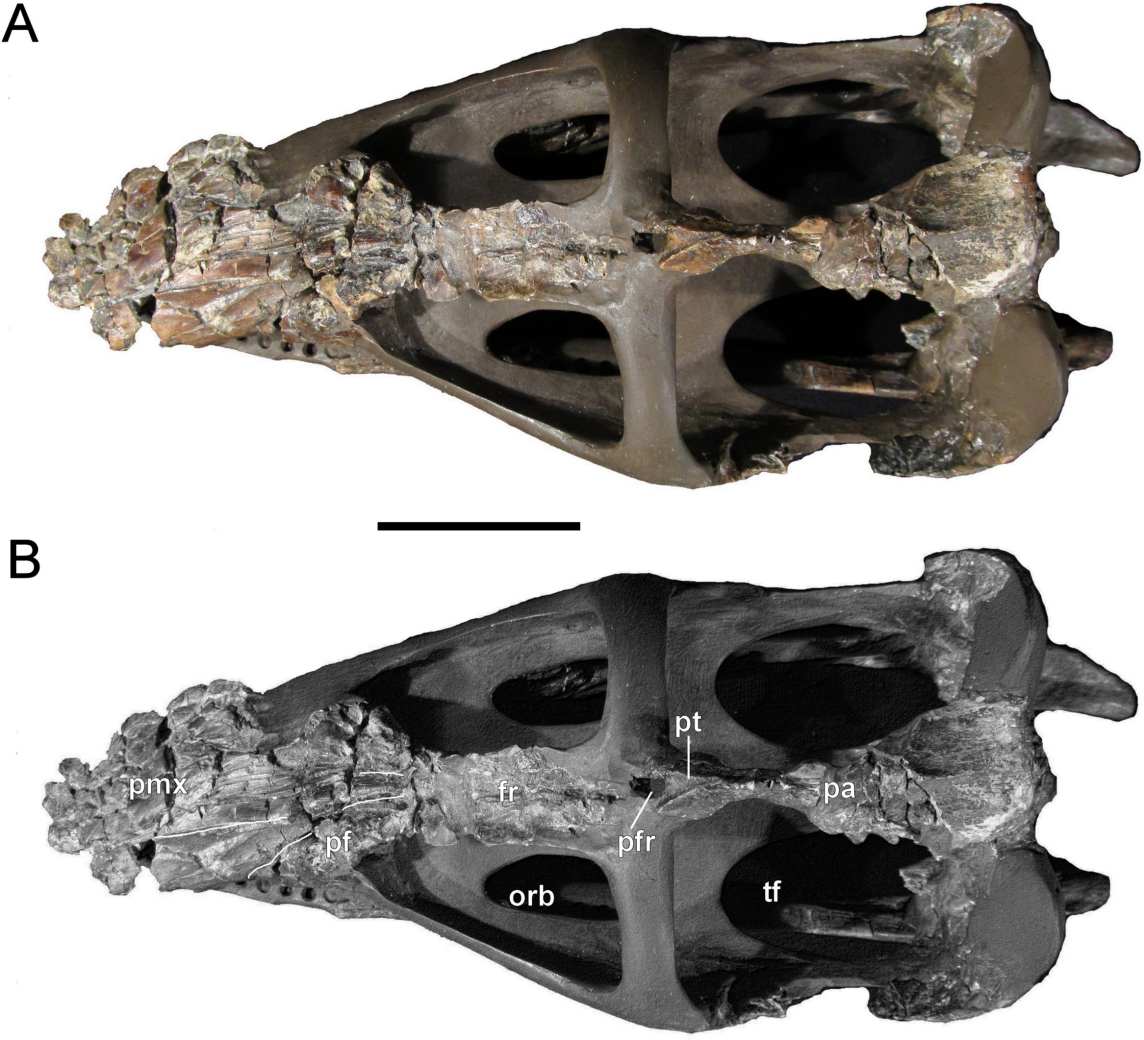
*Brancasaurus brancai*, GPMM A3.B4 (holotype). (A, B) Cranium and mandible in dorsal view. Scale bar = 50 mm. Abbreviations: fr, frontal; orb, orbita; pa, parietal; pf, prefrontal; pfr, pineal foramen; pmx, premaxilla; pt, parietal table; tf, temporal fenestra.

**Figure 6:**
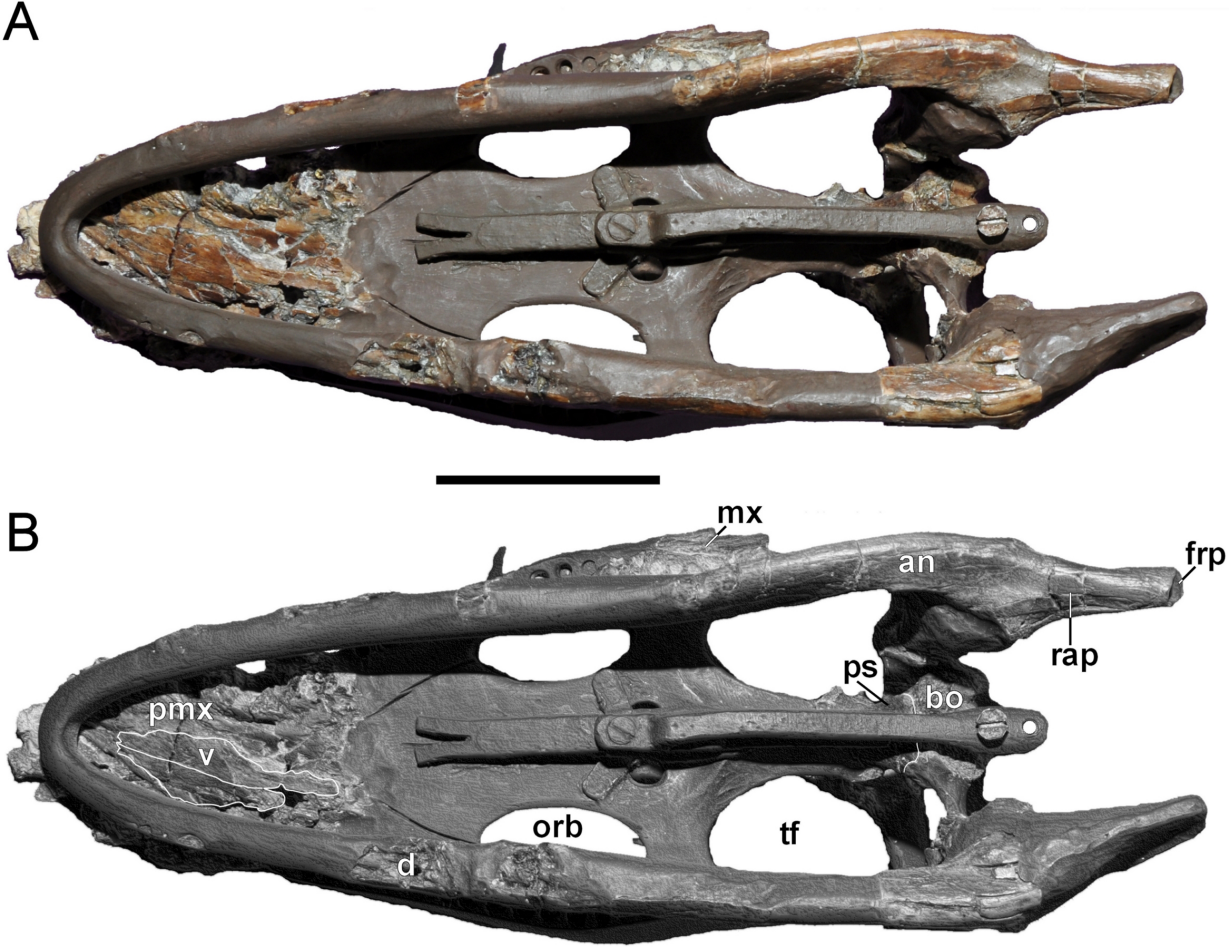
*Brancasaurus brancai*, GPMM A3.B4 (holotype). (A, B) Cranium and mandible in ventral view. Scale bar = 50 mm. Abbreviations: an, angular; bo, basioccipital; d, dentary; frp, facet of retroarticular process; mx, maxilla; orb, orbita; pmx, premaxilla; ps, parasphenoid; rap, retroarticular process; tf, temporal fenestra; v, vomer.

**Figure 7:**
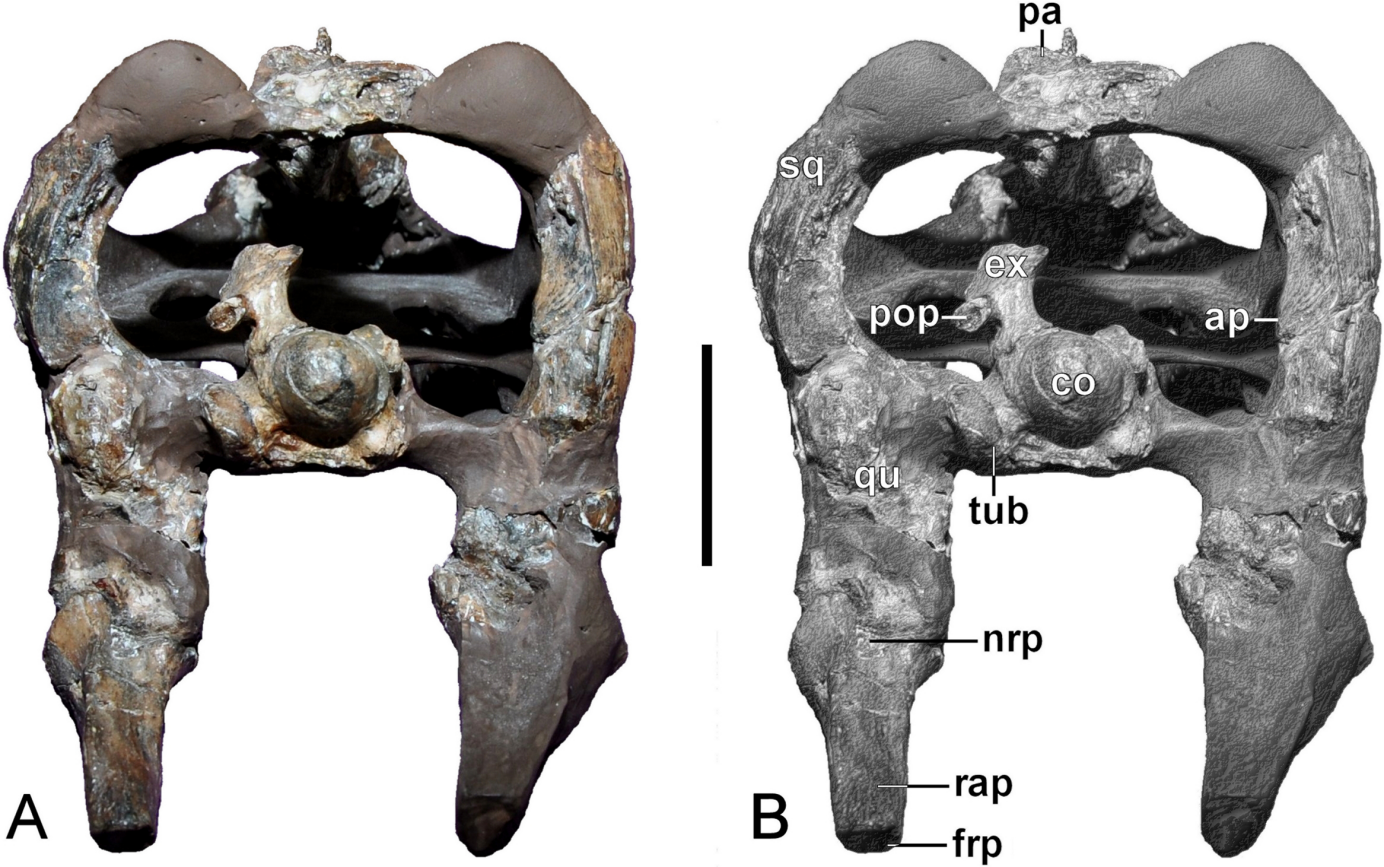
*Brancasaurus brancai*, GPMM A3.B4 (holotype). (A, B) Cranium and mandible in occipital view. Scale bar = 30 mm. Abbreviations: ap, articular surface of paroccipital process; co, condylus occipitalis; ex, exoccipital-opisthotic; frp, facet of retroarticular process; nrp, notch at retroarticular process; pa, parietal; pop, paroccipital process; qu, quadrate; rap, retroarticular process; tub, tubera.

**Figure 8:**
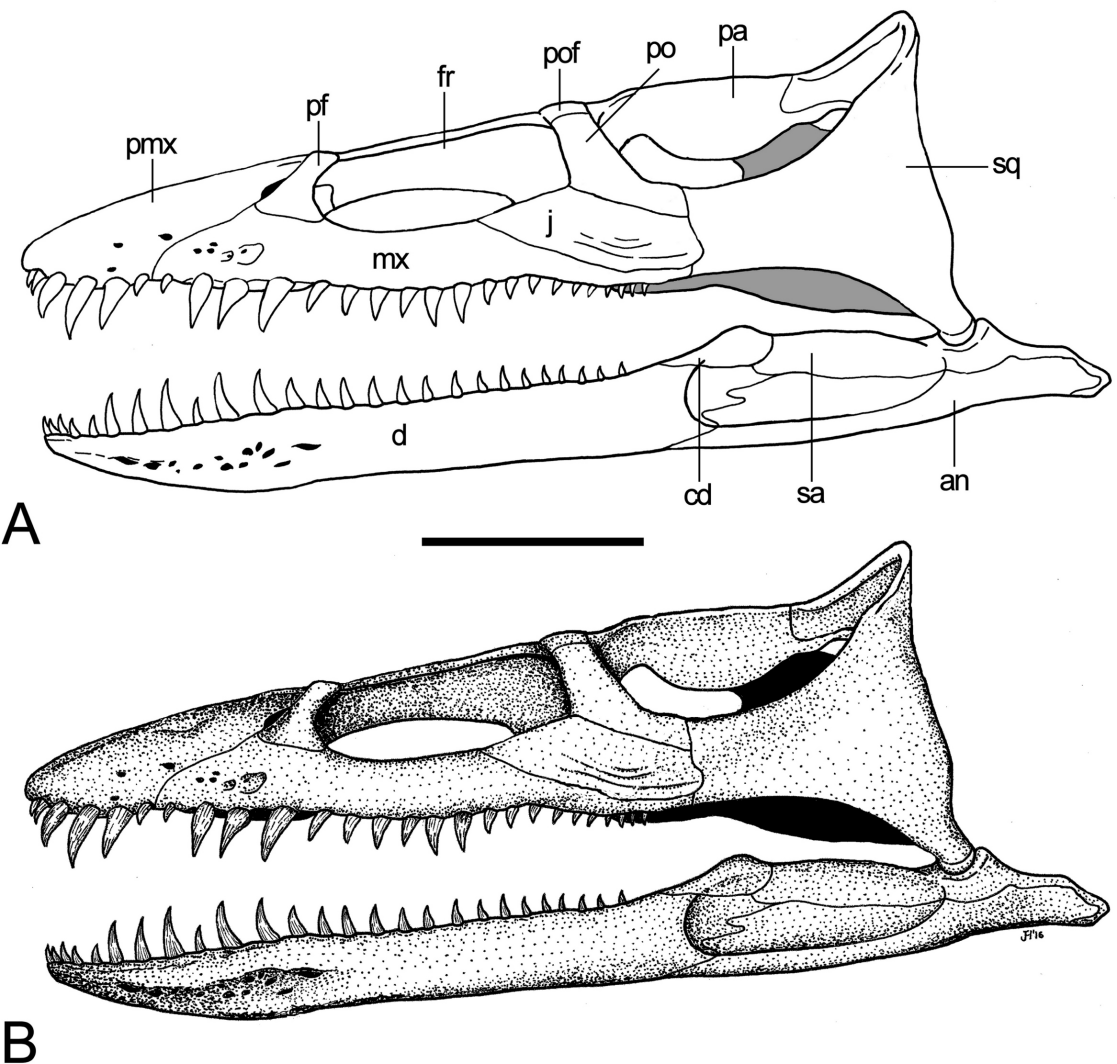
*Brancasaurus brancai*, reconstruction of cranium and mandible in lateral view. (A) Restoration, (B) legend to cranial elements. Scale bar = 50 mm. Abbreviations: an, angular; cd, coronoid; d, dentary; fr, frontal; j, jugal; mx, maxilla; pa, parietal; pf, prefrontal; pmx, premaxilla; po, postorbital; pof, postfrontal; sa, surangular; sq, squamosal.

**Figure 9:**
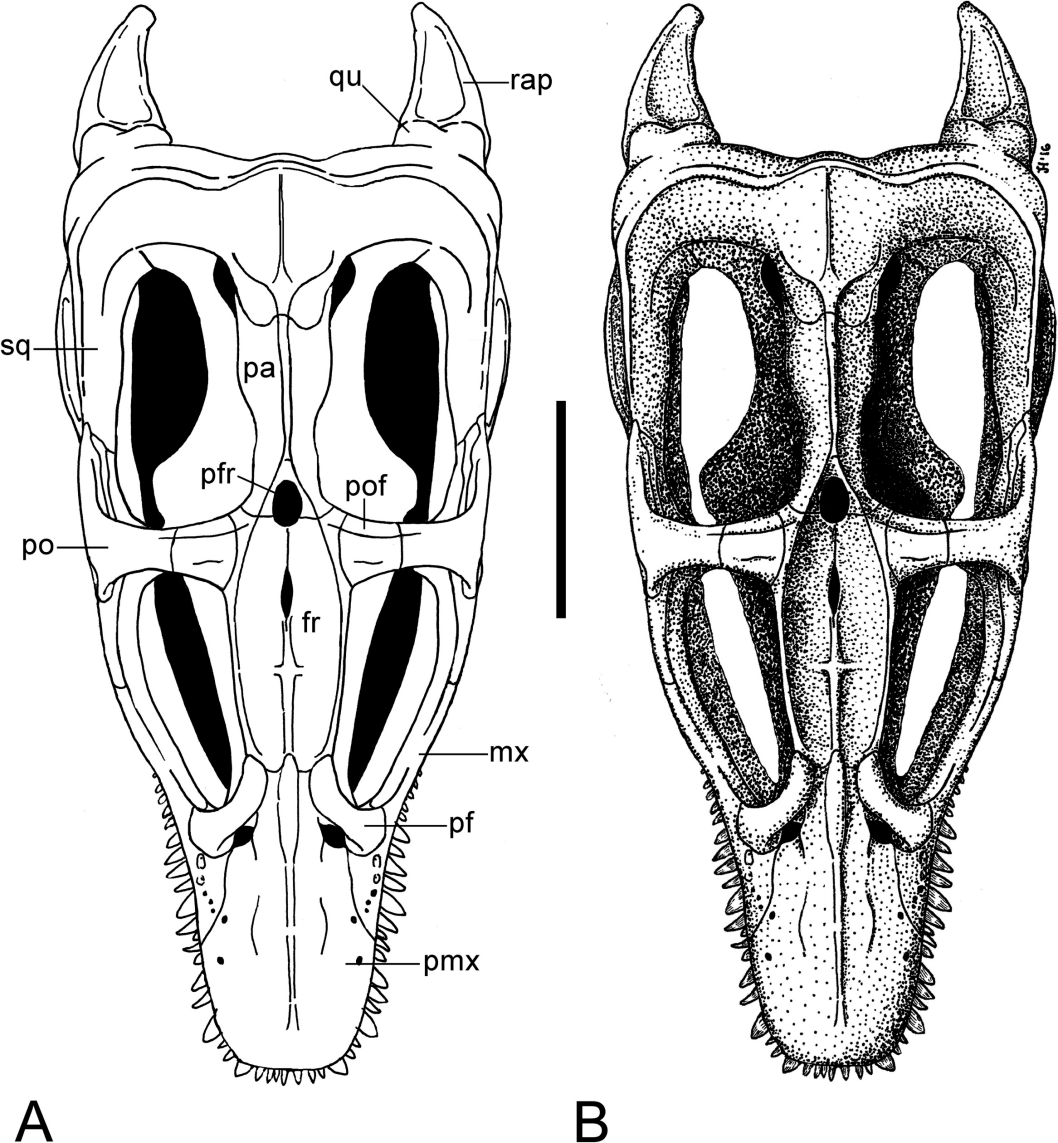
*Brancasaurus brancai*, reconstruction of cranium and mandible in dorsal view. (A) Restoration, (B) legend to cranial elements. Scale bar = 50 mm. Abbreviations: fr, frontal; mx, maxilla; pa, parietal; pf, prefrontal; pfr, pineal foramen; pmx, premaxilla; po, postorbital; pof, postfrontal; qu, quadrate; rap, retroarticular process; sq, squamosal.

The cranium of GPMM A3.B4 (Figs. 3–9) is rostrocaudally elongate and transversely narrow. The snout is tapered and lacks obvious evidence of diastemata. The dorsal profile is inclined caudally at approximately 15°relative to the longitudinal plane. Based on Wegner’s (1914: 243, Fig. 1) drawings, the orbits were originally of near equal length to the temporal openings, but apparently somewhat narrower. The ratio of pre-orbital skull to total skull length is 0.3 (see measurements in Table 1).

#### Premaxilla

The premaxillae are virtually complete, but severely fractured across both the dorsal surface and distorted left-hand side (Figs. 4–6). The external midline premaxillary suture is barely visible over most of its length. The facial processes of the premaxillae (*sensu* Taylor, 1992) can be recognised. Their transversely expanded rostral section appears to have been symmetrical in outline as indicated in Wegner’s (1914: 243, Fig. 1A) illustration. At their midline, the facial processes become vaulted to form a transversely narrow, rounded crest that tapers and terminates between the rostral margins of the orbits; this implies a dorsal contact with the frontals. What might be the premaxilla-maxilla suture is traceable along a crack on the left side of the skull, and corresponds with the premaxilla-maxilla contact depicted by Wegner (1914: 243, Fig. 1B). A similar suture is present on the right side. The external bony nasal openings cannot be delimited because of fracturing, although a thin medial ledge probably delimits the right narial margin. There is also no clear definition of the alveoli, but their approximate positions can be inferred from concavities representing their lingual walls; these are insufficient to confirm the number of teeth or their relative sizes. Wegner (1914: 251) originally depicted six premaxillary alveoli of varying diameters: the initial two being small, followed by three much larger tooth positions, and a final reduced alveolus at the premaxilla-maxilla suture.

A long furrow is present on the palatal surface medial to the alveolar row. Its floor is perforated by numerous foramina that equate to the dental lamina foramina of Rieppel (2001). Wegner (1914: 250) stated that unerupted replacement teeth were visible within these foramina, however this is no longer evident. Rostromedially directed ridges extend parallel to the dental lamina foramina, and converge apically where they enclose a triangular opening; this is bordered caudally by the broken edges of the vomers. It is unclear whether the vacuity is natural or an artefact of damage, but it coincides in position with the rostral vomerian fenestra of Buchy, Frey & Salisbury (2006).

Wegner (1914: 243, Fig. 1) illustrated additional structures on the premaxillae that were probably idealised to some degree. For example, the midline crest was shown to emerge further caudally, at around in the midsection of the rostrum (note that this structure was not described in Wegner’s text). In addition, the exact positioning of the external bony nasal openings were not specified, although, Wegner (1914: 243, Figs. 1A and 1B), depicted their medial edges incorporating the premaxillae. Wegner (1914: 250–251) also mentioned that the premaxilla-prefrontal sutures extend from the terminal ends of the facial processes; the premaxilla-maxilla sutures traced obliquely across the rostrum to contact the external bony nasal opening, and laterally to the margin of the alveolar row. The external surfaces of the premaxillae were apparently smooth, but with some shallow pitting.

#### Maxilla

Only a short rostral section, together with the caudal process of the left maxilla, is preserved (Fig. 4). Originally, however, both maxillae were much more complete (see Fig. 3) and included discernible sutural contacts with the premaxillae and prefrontals (see Wegner, 1914: 243, Figs. 1A and 1B); these are now represented by corresponding cracks. A large ovoid depression near the premaxillary suture and smaller depressions adjacently are remnants of the maxillary ornamentation.

At least one large maxillary alveolus (possibly for the second maxillary tooth) is observable near the premaxilla-maxilla suture (maximum diameter = 8.91 mm). The premaxillary palatal furrow continues onto the maxilla, and is likewise perforated by dental lamina foramina (Wegner, 1914: 251 stated that one of these exposed an replacement tooth crown). Along the midline, the palatal surface of the maxilla is vaulted, and would have formed a rostral cavity (*sensu* Buchy, Frey & Salisbury, 2006; or “central cavity” of Taylor, 1992) floored by the palatines. The caudal process of the left maxilla tapers and has a short contact to the horizontal ramus of the squamosal. Its termination lies parallel to the rostral third of the temporal opening. The maxilla also contacts the jugal. In contrast to Wegner’s (1914: 243, Fig. 1B) interpretation of these elements, the maxilla seems to be almost completely obscured by the jugal.

Four alveoli are preserved on the palatal surface of the left maxilla’s caudal extremity. Three of these are complete.

#### Prefrontal

Both prefrontals are incomplete but their original disposition can be inferred from Wegner (1914, p: 243, Figs. 1A and 1B). Most of the lateroventral portion of the left prefrontal is preserved (Fig. 5). The bone is thin with a smooth external surface. Wegner (1914: 243, Fig. 1A) showed a suture between the prefrontal and maxilla, which is now represented by a crack. The body of the prefrontal is caudomedially curved and terminates in the rostral third of the orbit where it contacts the frontal; Wegner (1914: 243, Fig. 1A) illustrated an additional, now missing mid-section of the bone. The dorsal-most portions of both prefrontals are preserved (more so on the right hand side) where they contribute to the orbital rims. Medially, the prefrontals are delimited by the premaxillae over their entire length. Wegner (1914: 243, Figs. 1A and 1B) also reconstructed the prefrontal involvement in the external bony nasal opening.

#### Frontal

The frontals create a rectangular dorsal bridge separating the orbits (Fig. 5), and as mentioned by Wegner (1914: 249), are depressed out of alignment in the reconstructed display mount of the skull. The sutural extremities of the frontals are broken but the rostroventral lobe-like contacts with the prefrontals and caudal processes of the premaxillae are still preserved. The dorsal surface of the frontals is smooth and concave with raised orbital margins. There is no obvious midline suture (contrary to Wegner, 1914: 249), but a dagger-like structure, formed by a weakly developed rostrocaudally running midline keel and another, shorter transverse keel as indictated in the rostral halves of the frontals (see Figs. 5 and 9). A small dorsomedian foramen (5.08/2.53 mm in maximum length/width) situated 13 mm in front of the pineal foramen equates to the “foramen frontale” described by Wegner (1914: 249). The lateral walls of the frontals are ventromedially inclined, imparting a triangular cross-section, and form a sharp edge at their intersection. The morphology of the conjoined frontals appears to be autapomorphic for *Brancasaurus brancai*, but the incomplete preservation and missing comparative data do not allow verification.

Wegner (1914: 243, Fig. 1A) also recorded a minor participation of the frontals within the margins of the temporal openings, and their enclosure of the pineal foramen in conjunction with the parietals.

#### Jugal

Wegner’s (1914: 243, Fig. 1B) interpretation of the left jugal appears to be partly incorrect. The bone is represented by a roughly triangular fragment, which contacts the postorbital via a rostrodorsally directed suture (Fig. 4). Most of Wegner’s (1914, Fig. 1B) caudad maxillary process seems to be formed by the jugal, which laterally overlay the maxillary almost completely. The caudal extremity of the jugal reaches parallel to the rostral third of the temporal opening and overlaps the horizontal ramus of the squamosal. Wegner (1914) did not describe the jugal of GPMM A3.B4, but his figure (Wegner, 1914: 243, Fig. 1B) indicates that the element was originally rectangular in shape and contributed to the bony edge of the orbit. The postorbital suture likewise extended much further (covering around two-thirds the length of the jugal), and the maxilla bordered its entire ventral margin.

#### Postorbital

A component of the left postorbital is preserved in articulation with the jugal (Fig. 4). When complete, it would have participated in the rostral wall of the temporal opening and overlapped the horizontal ramus of the squamosal (Wegner, 1914: 243, Fig. 1B). Although Wegner’s (1914: 249) discussion is brief, he did show (Wegner, 1914, Fig 1) that the left postorbital was originally intact and formed the caudoventral frame of the orbit. In addition, it seems to have had a short dorsal contact against the postfrontal and an elongate ventral suture with the jugal.

#### Postfrontal

There is no trace of a postfrontal in the restored skull, but an incomplete bone stored in the GPMM collection represents one of these elements. It has a smooth, flat external surface and bears a buttress-like structure at its ventral midsection. The fragment becomes higher and wider towards the probable medial side and flatter towards the opposing surface. Wegner (1914: 249) reconstructed the postfrontal forming the margin of the left orbit and bordering the temporal opening. It reportedly contacted the frontal and was loosely associated with the postorbital.

#### Parietal

The parietals are highly fractured but have been pieced together from several sections and fixed in modelling putty (Figs. 4 and 5). Rostrally, the parietals enclose the pineal foramen (2.82 mm in maximum width); this has been restored along its left lateral edge and is missing its contact with the frontals. As noted by Benson et al. (2013a), a conspicuous triangular fossa (= “parietal table” of Druckenmiller & Russell, 2008a) tapers proximally from the pineal foramen. It is enclosed by two thin ridges, which proximally meet into a pointed apex and merge with the parietal crest. The latter is now incomplete but following Wegner’s (1914: 243, Fig. 1A) restoration it seems to have originally extended caudally up until the parietal-squamosal contact. Most of the parietal mid-section has been reconstructed but sections of the sloping parietal walls are still present. In opposition to Benson et al. (2013a, Appendix S1: p. 5, character 206), this region of the parietals does not exceed “more than half the transverse width of the posterior cranium,” rather only around a third (ratio of 0.31 based on maximum widths of 37/118 mm).

Wegner (1914: 249) described the parietals as massive elements with a triangular cross-section. He additionally reported a thin ridge extending forward from the pineal foramen, and adjacent “zygapophysis-like” processes arching over the frontals. Sections of what might have been the parietal walls were also mentioned; the ventral surfaces of the parietals were apparently vaulted with a rounded midline keel.

#### Squamosal

Wegner’s (1914: 248–249) convoluted description of the squamosals was brief. Our examination detected a partly restored left horizontal ramus (maximum length = 9.45 mm) that contacts the maxilla ventrally, as well as both the jugal and postorbital dorsally (Fig. 4). Both the left and right ventral rami enclose remnants of the quadrates, although the sutures are indistinct (see Wegner, 1914: 250), and suggest that inclination of the suspensorium was minimal. In lateral view, an unusual triangular process (maximum length/height at base = 15/14 mm) projects forward from the left dorsal ramus of the squamosal arch. The broken remnant of a corresponding process is likewise preserved on the right squamosal. Wegner (1914: 243, Fig. 1B) did not illustrate these structures, and archival slide photographs of the original skeletal mount (Fig. 3A) show that these are actually parts of the dorsal edges of the originally complete lateral rami.

In occipital view, the dorsal rami of the squamosals arch around the post-temporal openings, but these are incomplete towards the parietal-squamosal contact. A vertically flared transverse expansion is present at the squamosal apex, which bears a raised inter-squamosal suture and projects caudally as a small bulge along the midline (Fig. 5; also evident in the GPMM A3.B2 holotype of *Gronausaurus wegneri*). The occipital faces of the dorsal rami bear a continuous ridge that follows the curvature of the squamosal arch, and presumably served as attachment for the neck musculature (*sensu* Taylor, 1992). The contact surface for the paroccipital process descending from the exoccipital-opisthotic is evidenced on medial face of the right dorsal ramus, and is approximately level with the dorsal edge of the occipital condyle.

#### Quadrate

Both quadrates are preserved but have been covered by layers of modelling putty (Fig. 7). This has obscured most of the bone surfaces, and Wegner’s (1914: 250) description provides little additional information. Nevertheless, the exposed left quadrate does reveal a rounded lateral articular condyle with a squared profile in occipital aspect. The medial condyle is broader and offset ventrally; this imparts an oblique orientation to the glenoid fossa. The rear surface of the left quadrate is inset above the condylar articulation, and a low ridge (maximum length = 10 mm) runs from the medial edge above the medial condyle towards the basioccipital. A squamosal suture is not evident.

#### Vomer

**Figure 10:**
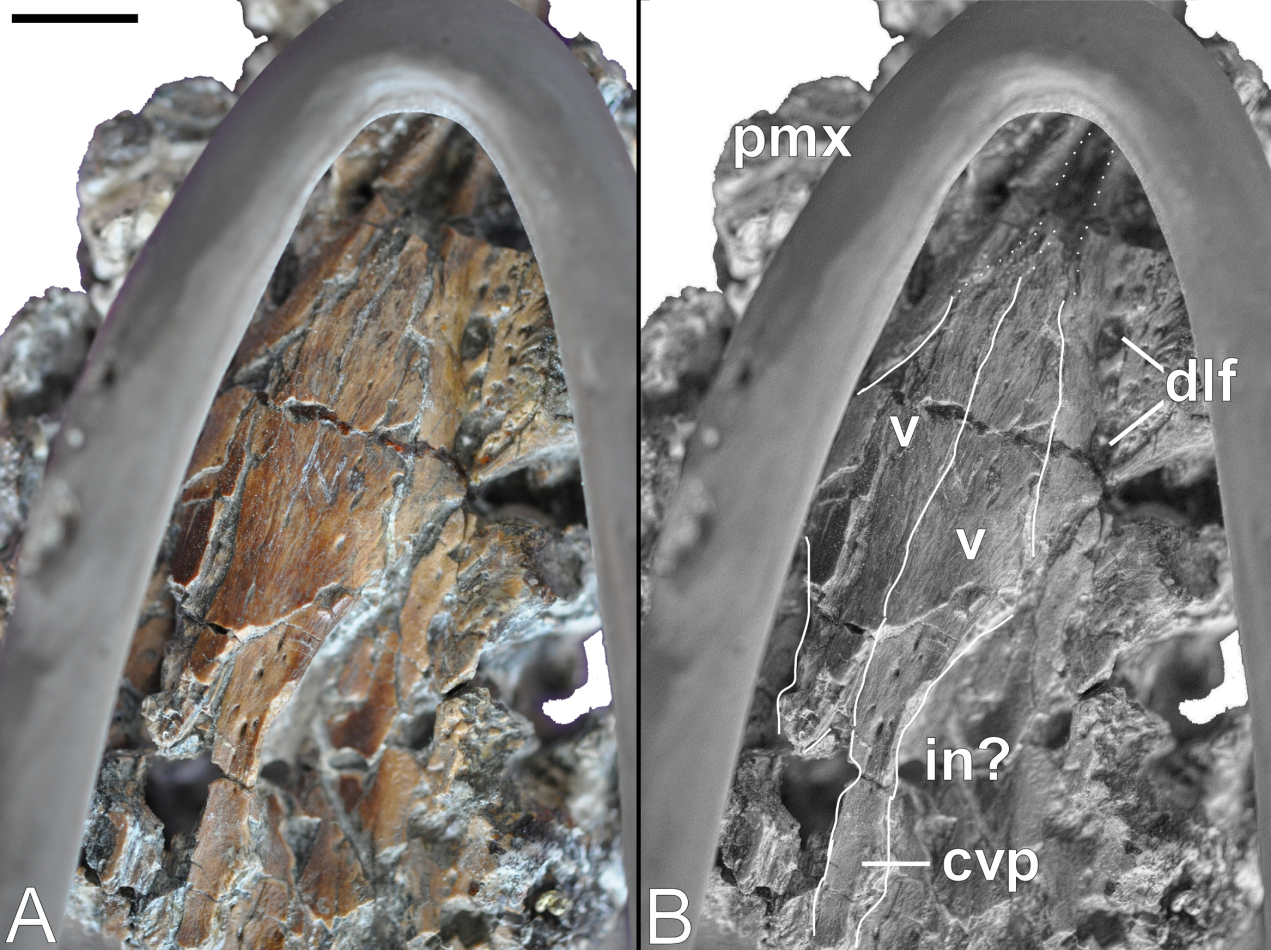
*Brancasaurus brancai*, GPMM A3.B4 (holotype). (A, B) Palate. Scale bar = 10 mm. Abbreviations: cvp, caudal vomeral process; dlf, dental lamina foramina; in, possibly interal naris; pmx, premaxilla; v, vomer.

Both the left and right vomers are observable in palatal view (Fig. 10), and generally conform to the depiction in Wegner (1914: 243, Fig. 1C). They contribute to the midline of the palate, and although slightly distorted, maintain both a straight medial inter-vomerine suture and tapered lateral contact with the enclosing premaxillae. The truncated apex of the vomers exposes the smooth-walled rostral cavity (possibly a rostral vomerian fenestra: Buchy, Frey & Salisbury, 2006).

Wegner (1914: 243, Fig. 1C) envisaged a pair of medial sutures with the pterygoids. These separated the vomers caudally, and were bordered laterally by the palatines. Sadly, all of these elements are now lost and the remaining palatal surface is severely fractured (but numerous small nutrient foramina are still evident). Disposition of the internal bony nasal opening (= caudal vomerian fenestra: Buchy, Frey & Salisbury, 2006) is impossible to infer accurately. However, the long and slender caudal extremity of the left vomer is laterally embayed and preserves a finished edge that might represent part of its medial margin (compare with Wegner, 1914: 243, Fig. 1C).

#### Palatine

Wegner (1914: 243, Fig. 1C) figured rostral components of both palatines, as well as their contacts with the vomers, pterygoids, and lateral borders of the maxillae. Wegner (1914: 251) stated that the palatines formed part of the bony nasal openings, but this is impossible to confirm given the current state of preservation.

#### Pterygoid

Wegner (1914: 250) identified parts of the pterygoids in situ between the caudal extremities of the vomers. Only a non-descript remnant of the quadrate ramus of the left pterygoid now remains in contact with the quadrate.

**Figure 11:**
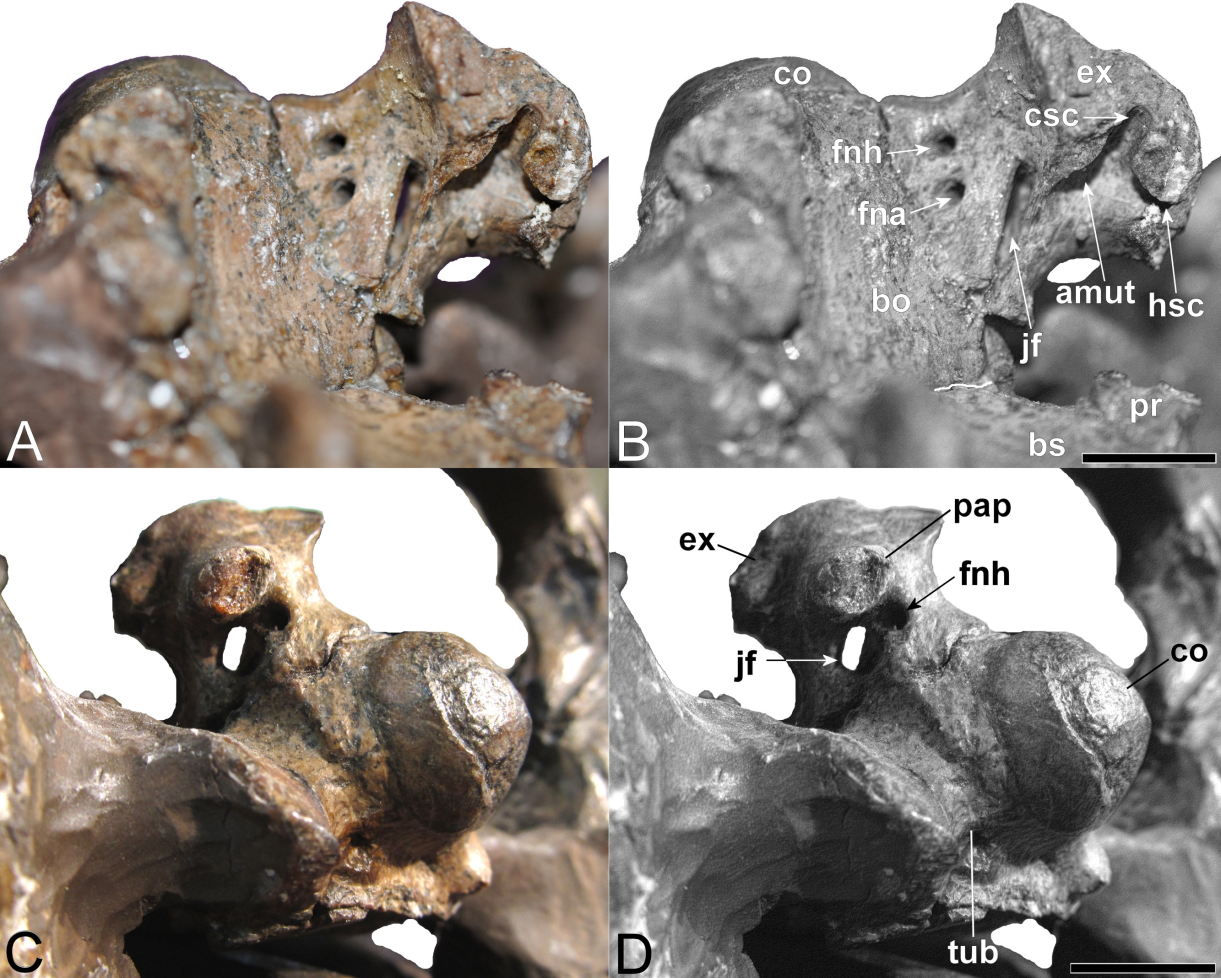
*Brancasaurus brancai*, GPMM A3.B4 (holotype), braincase components. (A, B) Basioccipital in dorsal, and exoccipital-opisthotic in medial views. (C, D) Basioccipital and exoccipital-opisthotic in lateral view. Scale bars = 10 mm. Abbreviations: amut, chamber for ampulla and utriculus; bs, basisphenoid; bo, basioccipital; co, condylus occipitalis; csc, caudal semicircular canal; ex, exoccipital-opisthotic; fna, foramen for accessory nerve (XI); fnh, foramen for hypoglossal nerve (XII); hsc, opening for the horizontal semicircular canal; jf, jugalar foramen for glossopharyngeal [IX], vagus [X] and accessory [XI] nerves and perhaps the jugular vein; pop, paroccipital process; pr, prootic; tub, tubera.

#### Basioccipital

**Figure 12:**
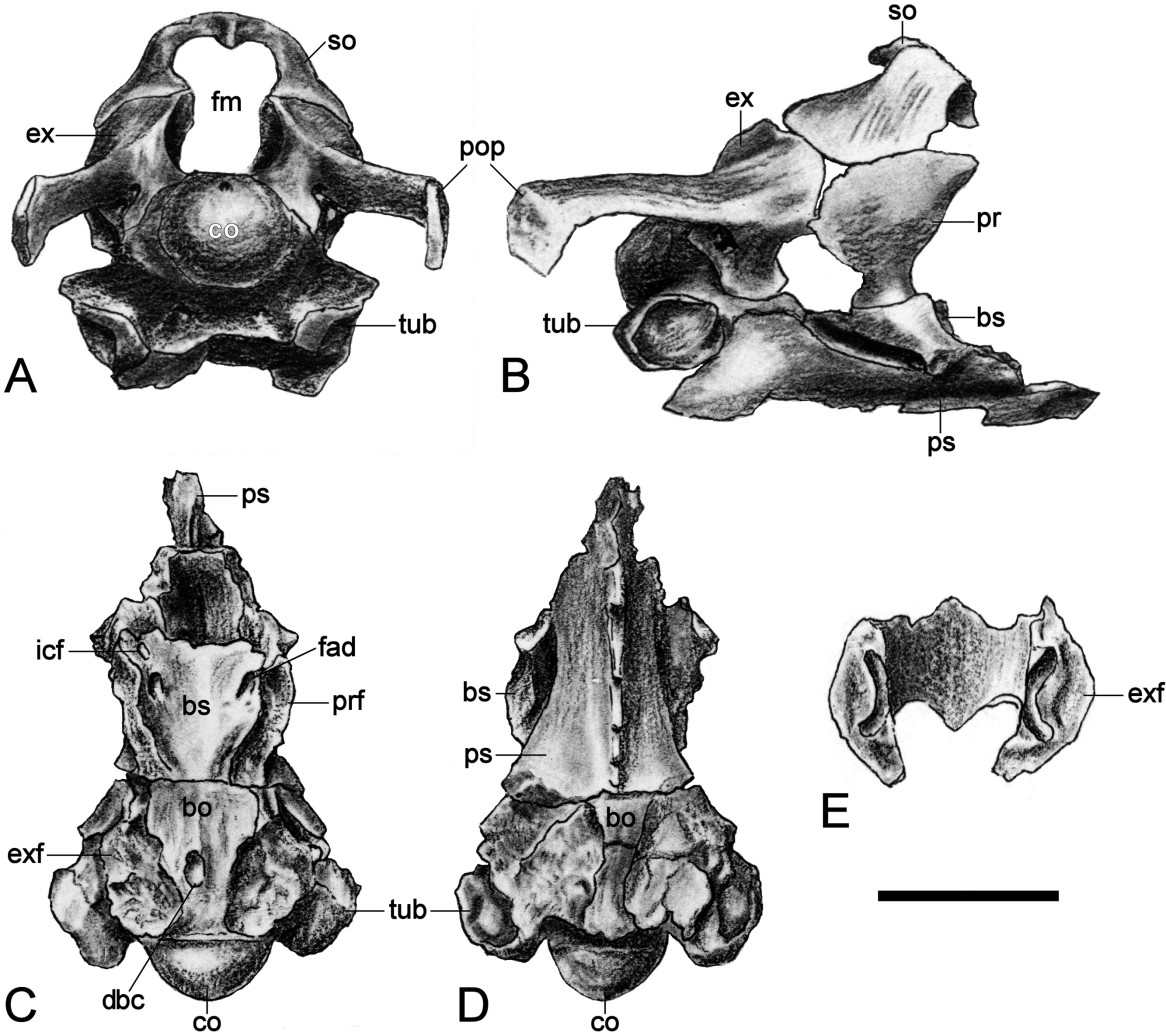
*Brancasaurus brancai*, GPMM A3.B4 (holotype), braincase as depicted in Wegner (1914). (A) Articulated braincase in occipital and (B) lateral views. Base of braincase in dorsal (C), and (d) ventral views. (E) Supraoccipital in ventral view. Scale bar = 20 mm. Abbreviations: bo, basioccipital; bs, basisphenoid; co, condylus occipitalis; dbc, depression in basioccipital; ex, exoccipital-opisthotic; exf, facet to exoccipital-opisthotic and prootic; fad, foramen probably for cranial nerve (abducens) VI; fbc, facet in basioccipital; fm, foramen magnum; icf, internal carotid foramen; pop, paroccipital process; pr, prootic; prf, prootic facet; ps, parasphenoid; rr, recessus retriculus; so, supraoccipital; tub, tubera.

The restored basioccipital is caudally inclined with a hemispherical occipital condyle (maximum horizontal/vertical diameter = 16/15 mm, Figs. 11 and 12). The condylar articular surface is weakly circumscribed by an inset area that becomes more prominent dorsally. A distinct notochordal pit is positioned vertically above the transverse condylar midline. It is aligned longitudinally with an oval depression on the dorsal surface of the basioccipital where it contributed to the floor of the cavum cranii; this could have accommodated the notochord (e.g., as in ichthyosaurians: Kear, 2005b). The right basioccipital tuber is damaged but the left is complete and ventrolaterally oriented. The lateral facet for the pterygoid process of the basioccipital (maximum vertical dimension = 12 mm) was longitudinally expanded and had a concave occipital surface. The caudal face of the basioccipital tuber is concave. Wegner (1914: 244) mentioned that the exocipital-opisthotic facets are bilobed with a narrow medial constriction. The intervening neural canal forms a gently concave floor and is transversely expanded where it enters the endocranial space. The transverse basioccipital-basisphenoid suture, as well as the contact with the parasphenoid, are closely adherent but retain obvious separation as would be expected in an osteological immature individual (*sensu* Brown, 1981). The ventral surface of the basioccipital is obscured by steel mounting armature. Nonetheless, the figures from Wegner (1914, plate 6, Fig. 2) show that this was flat and that the parasphenoid underlapped the basioccipital via a short (“5 mm” in length) medial protrusion (Wegner, 1914: 244).

#### Basisphenoid

The basisphenoid is exposed in dorsal aspect and delineated caudally by the basioccipital suture, as well as its lateral contacts with the underlying parasphenoid. The dorsal surface of the basisphenoid is concave and rostrally declined. As illustrated by Wegner (1914, plate 6), long irregular furrows (= “cochlear facets” *sensu* Hampe, 2013: 475) inscribe the sides of the basisphenoid and house the broken remnants of the prootics. A prominent foramen is visible on the left rostral edge immediately below the dorsum sellae. This corresponds in position with the internal carotid foramen (Benson et al., 2011b: 568, Fig. 4A; Sato et al., 2011: 318, Fig. 3A), and is associated with a second, slightly larger foramen probably for the abducens (VI) nerve (Sato et al., 2011: 318, Fig. 3A). Wegner (1914, plate 6, Fig. 8) figured the exit point of this latter foramen (labelled “f.ca.x” = foramen caroticum externum: Wegner, 1914: 246) at the intersection of the basisphenoid and parasphenoid.

Only the concave left side of the sella turcica now remains, and is separated from the dorsum sellae by a transverse keel. A short, incomplete ledge on the lateroventral side of the sella turcica accords with the “lower cylindrical process” of Carpenter (1997: 205).

#### Parasphenoid

The parasphenoid is largely hidden behind the reinforcing display framework but its broad contact with the basioccipital is still evident; this underlaps the entire transverse width of the basioccipital and apparently also extended caudally below the basioccipital as a medial protrusion. Wegner’s (1914, plate 6, Fig. 2) drawing additionally shows the cultriform process, which bore a narrow keel along its entire length and tapered well beyond the length of the basisphenoid.

The lateral sides of the parasphenoid were sloped within the caudal interpterygoid vacuities.

#### Exoccipital-opisthotic

The left exoccipital-opisthotic is preserved in articulation with the basioccipital (Fig. 11). Its base is bilobed (slightly tapering rostrally), and its main body is successively perforated along its medial wall by three foramina. Rostrally, there is a slit-like jugular foramen probably for the glossopharyngeal (IX) and vagus (X) nerves: (Romer, 1956; Hopson, 1979). Ventrally, in about the midsection of the base of the main body, there is a foramen that might have served the accessory nerve (XI) followed by a caudal opening, probably for the passage of the hypoglossal nerve (XII: compare Sachs, Lindgren & Siversson, 2016). There are traceable impressions for the caudal vertical and horizontal semicircular canals of the membranous inner ear. The base of the dorsal branch is expanded and possibly housed the ampulla and utriculus (*sensu* Benson et al., 2011b).

In lateral view, the exoccipital-opisthotic preserves the broken base of the paroccipital process. Wegner (1914, plate 6, Figs. 8 and 9) reconstructed this structure as a transversely flattened, caudoventrally directed rod, with an expanded distal extremity that contacted the squamosal and enclosed the cranioquadrate passage. The paroccipital processes seemingly did not extend below the level of the occipital condyle. Ventral to the paroccipital process base, the external face of the exoccipital-opisthotic bears a small caudally situated opening for the hypoglossal nerve (XII), and a larger adjacent rostral foramen for the glossopharyngeal (IX), vagus (X) and accessory (IX) nerves and perhaps the jugular vein.

The concave medial walls of the exoccipital-opisthotic enclosed the foramen magnum. There was also an articulation with the prootic that enclosed the fenestra ovalis (see Brown, 1981; Cruickshank, 1994; Carpenter, 1997; Sato et al., 2011).

#### Supraoccipital

The supraoccipital has been lost. Wegner (1914, plate 6, Figs. 7–9) described a broad, arching element that medially constricted the foramen magnum via transversely (and caudally) expanded exoccipital-opisthotic facets. The external dorsal midline was produced into an occipital crest (*sensu* Andrews, 1910; Brown, Milner & Taylor, 1986; Sato et al., 2011) with rostral and caudal projections; the adjacent articulation surface for the parietals was apparently rugose and inclined (Wegner, 1914: 248).

#### Prootic

Wegner (1914: 248) described the prootics as trapezoidal in profile with a narrow ventral margin and dorsal contacts against both the exoccipital-opisthotics and supraoccipital. The rostral edge was almost vertical and bore weak vertical dorsal and ventral protrusions. The rear margin of the prootic was straight and formed part of the fenestra ovalis at its base (Carpenter, 1997; Sato et al., 2011). Wegner (1914: 248) states that the lateral side of the prootic was gently convex, whereas medially it was inset by the recessus utricularis (Wegner, 1914, plate 6, Fig. 5). This also purportedly comprised an open superior semicircular canal with a foramen that penetrated the exoccipital-opisthotic facet (perhaps serving as exit for the horizontal semicircular canal: *sensu* Sato et al., 2011). Only the bases of the prootics are still preserved.

#### Endoneurocranial cast

**Figure 13:**
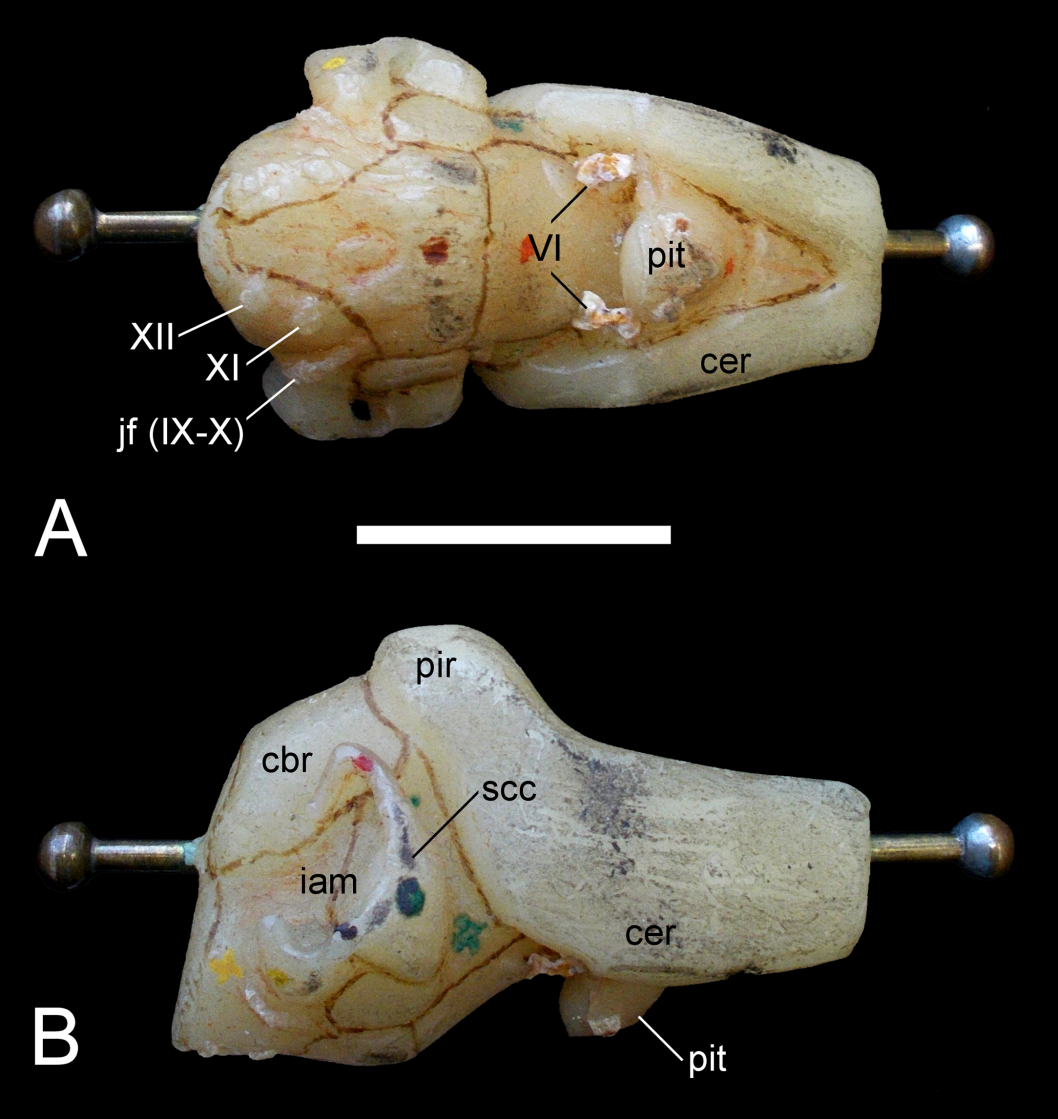
*Brancasaurus brancai*, GPMM A3.B4 (holotype), endoneurocranial wax cast (SMF R4076). (A) Ventral, and (B) lateral views. Scale bar = 30 mm. Abbreviations: cbr, cerebellar region; iam, internal auditory meatus; jf (IX–X), jugular foramen opening for the glossopharyngeal (IX) and vagus (X) nerves; pit, pituitary fossa; scc, semi circular canal; VI, abducens foramina; XII, foramina for the hypoglossal (XII) nerve branches.

Edinger (1928), Edinger (1930) and Hopson (1979) summarized the endoneurocranial impressions on SMF R4076 as depicting only the hindbrain, inner ear cavity, and pituitary fossa (Fig. 13). Our comparison with the corresponding basicranial elements of GPMM A3.B4 confirmed reconstruction of the cerebellar area, including impressions of the internal auditory meatus and semicircular canals, as well as infillings of the canals for branches of the hypoglossal (XII), accessory (XI), glossopharyngeal (IX) and vagus (X) nerves observable from the exoccipital-opisthotic. The pituitary fossa and probable abducens (VI) foramen are also indicated (see endocranial interpretation of Carpenter, 1997).

### Mandible and dentition

#### Dentary

A fragment of the right dentary ramus, and parts from the rostral and mid-section of the left ramus are included within the restored mandible. The external surfaces of these bones are smooth, and the ventral edge of the left dentary is narrow; the dentigerous margin retains remnants of the alveoli.

Wegner (1914: 252) briefly remarked on the short mandibular symphysis and the presence of 21 alveoli in a 140 mm long section of the right dentary. The rostral-most of these were small, but the subsequent alveoli increased in size towards the 10th tooth position, after which their diameter remained consistent.

#### Surangular, angular and articular

The post-coronoid components of both mandibular rami are preserved, but extensive fracturing and distortion prevents identification of the sutures. The dorsal side of the surangular is transversely narrow and slightly rostrodorsally curved along its dorsal profile; this implies a low coronoid eminence. The lateral surface of the surangular is conspicuously depressed to form an oval trough (see Benson et al., 2013a) that extends from the broken rostral end of the mandible caudally to the level of the glenoid fossa. The medial side of the surangular is concave where it forms the Meckelian canal (about 270 mm of this is visible), and is enclosed dorsally by a shelf of bone with a corresponding ventral lip; these likely contacted the splenial and prearticular. The caudoventral margins of the mandibular rami probably incorporated the angulars, which are transversely rounded and expanded towards the mandibular glenoid fossae (what might be the surangular-angular suture is observable just below the glenoid rostral wall). The glenoid articulations are otherwise covered by modelling putty but were clearly situated behind the condylus occipitalis.

The left retroarticular process is long and sub-rectangular in profile. Its ventral margin is longitudinally straight and transversely rounded; the dorsal edge is dorsorostrally inclined. A prominent notch is evident on the rear articular face of the glenoid fossa. This could have accommodated a tendinous insertion, with another prominent circular scar (9.4 mm in diameter) for the m. depressor mandibulae visible at the apex of the retroarticular process.

*Dentition:* The teeth of GPMM A3.B4 have been lost. However, Wegner (1914: 251–252) described them as being “awl-shaped,” long and slender (Wegner, 1914, plate 6, Fig. 10). The labial side of each tooth crown was smooth. The enamel surfaces were otherwise ornamented by coarse ridges (up to 19 in the largest tooth fragment), which terminated (“Auskeilen” (“pinched out”) according to Wegner, 1914: 252) just proximal to the apex.

### Axial skeleton

#### Atlas-axis complex

**Figure 14:**
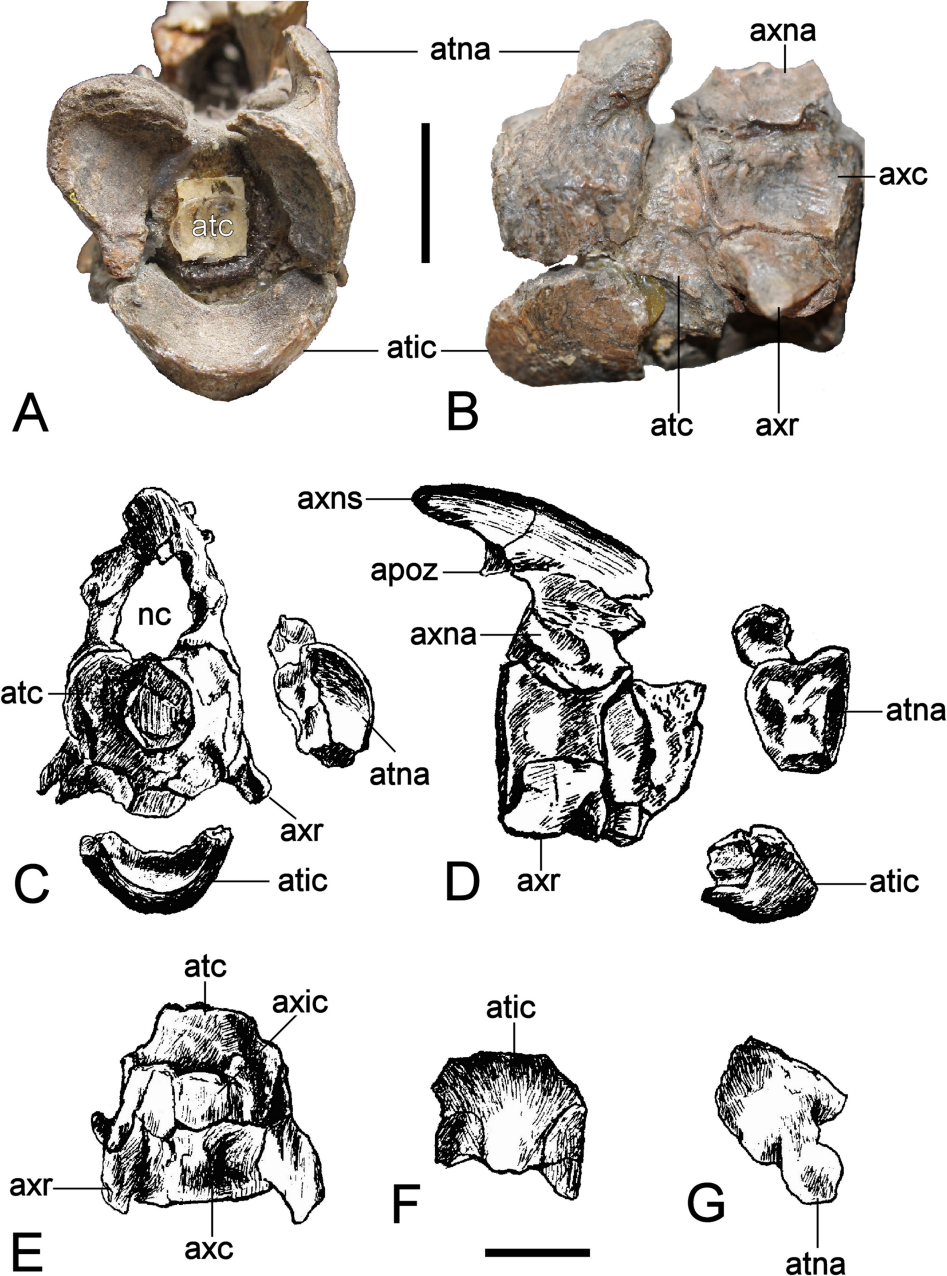
*Brancasaurus brancai*, GPMM A3.B4 (holotype). (A, B) atlas-axis complex as preserved, (A) cranial, and (B) lateral views. (C–G) Atlas-axis complex as depicted by Wegner (1914) in (C) cranial, (D) lateral, and (E) ventral views. (F) Atlas intercentrum in ventral view; (G) atlas neural arch in lateral view. Scale bars = 10 mm. Abbreviations: apoz, axis postzygapophysis; atc, atlas centrum; atic, atlas intercentrum; atna, atlas neural arch; axc, axis centrum; axna, axis neural arch; axns, axis neural spine; axr, axis rib; nc, neural canal.

The individual components of the atlas-axis complex are not fused (Fig. 14). The atlas centrum exceeds the axis centrum in length (Table 2). Cranially, the deep atlantal cup is rimmed ventrally by the atlas intercentrum and dorsolaterally by the atlas neural arch pedicles. It is caudally demarcated by the atlas pleurocentrum (= atlas centrum *sensu* Druckenmiller & Russell, 2008b). The craniad edge of the atlas intercentrum is concave and protrudes beyond the level of the atlas neural arch. The convex ventral surface is produced into a mid-line bulge. The lateral contacts between the atlas intercentrum and atlas neural arch pedicles are linear (although this is slightly distorted on the left side). A remnant of the atlas neural canal wall is preserved on the right-hand side, as are the bases of the axis neural arch pedicles. Wegner (1914: 254, Fig. 2) showed both of these to be originally complete, and enclosing an oval neural canal. The axis neural spine was low and projected beyond the centrum by about half its length; the dorsal margin was rounded. The postzygapophyses were also elevated and horizontally oriented.

Part of the atlas pleurocentrum is exposed on the lateral surface of the atlas-axis complex, and is bordered ventrally by the concave facet for the atlas rib. The axis rib base extends along the entire ventrolateral length of the axis centrum but is more dorsally placed than the atlas rib. The remainder of the lateral centrum surface is deeply concave. Dorsally, the elliptical bases of the axis neural arch pedicles enclose the neural canal; this is widest at its mid-section where an opening between the atlas and axis neural arch was present.

**Table 2:** Vertebral measurements (mm) of *Brancasaurus brancai* (GPMM A3.B4) (height was measured caudally).

**Atlas-axis complex**	
Length	31
Width of atlas centrum cranially	19
Height of atlas centrum	21
Width of axis centrum	20
Height of axis centrum	18
**Additional cervical vertebrae**	
Cervical vertebra 3	
Length	16
Height	16
Cervical vertebra 4	
Length	18
Height	17
Cervical vertebra 5	
Length	18
Height	18
Cervical vertebra 6	
Length	20
Height	20
Cervical vertebra 7	
Length	20
Height	23
Cervical vertebra 8	
Length	21
Height	22
Cervical vertebra 9	
Length	23
Height	23
Cervical vertebra 10	
Length	23
Height	25
Cervical vertebra 11	
Length	24
Height	25
Cervical vertebra 12	
Length	26
Height	25
Cervical vertebra 13	
Length	26
Height	29
Cervical vertebra 14	
Length	28
Height	27
Cervical vertebra 15	
Length	31
Height	30
Cervical vertebra 16	
Length	30
Height	29
Cervical vertebra 17	
Length	32
Height	33
Cervical vertebra 18	
Length	32
Height	32
Cervical vertebra 19	
Length	34
Height	33
Cervical vertebra 20	
Length	35
Height	33
Cervical vertebra 21	
Length	38
Height	35
Cervical vertebra 22	
Length	38
Height	34
Cervical vertebra 23	
Length	39
Height	37
Cervical vertebra 24	
Length	38
Height	35
Cervical vertebra 25	
Length	39
Height	37
Cervical vertebra 26	
Length	40
Height	38
Cervical vertebra 27	
Length	41
Height	39
Cervical vertebra 28	
Length	42
Height	40
Cervical vertebra 29	
Length	43
Height	41
Cervical vertebra 30	
Length	42
Height	41
Cervical vertebra 31	
Length	43
Height	41
Cervical vertebra 32	
Length	42
Height	43
Cervical vertebra 33	
Length	43
Height	41
Cervical vertebra 34	
Length	42
Height	43
Cervical vertebra 35	
Length	42
Height	41
Cervical vertebra 36	
Length	40
Height	43
Cervical vertebra 37	
Length	40
Height	41
**Pectoral vertebrae**	
Pectoral vertebra 1	
Length	37
Height	41
Pectoral vertebra 2	
Length	37
Height	39
Pectoral vertebra 3	
Length	38
Height	44
**Dorsal vertebrae**	
Dorsal vertebra 1	
Length	37
Height	42
Dorsal vertebra 2	
Length	35
Height	43
Dorsal vertebra 3	
Length	35
Height	43
Dorsal vertebra 4	
Length	36
Height	40
Dorsal vertebra 5	
Length	35
Height	41
Dorsal vertebra 6	
Length	34
Height	42
Dorsal vertebra 7	
Length	33
Height	40
Dorsal vertebra 8	
Length	33
Height	39 i.c.
Dorsal vertebra 9	
Length	33
Height	38 i.c.
Dorsal vertebra 10	
Length	32
Height	37
Dorsal vertebra 11	
Length	33
Height	36
Dorsal vertebra 12	
Length	31
Height	37
Dorsal vertebra 13	
Length	31
Height	37
Dorsal vertebra 14	
Length	32
Height	36
Dorsal vertebra 15	
Length	30
Height	32
Dorsal vertebra 16	
Length	32
Height	35
Dorsal vertebra 17	
Length	30
Height	39
Dorsal vertebra 18	
Length	32
Height	33
Dorsal vertebra 19	
Length	30
Height	31
**Sacral vertebrae**	
Sacral vertebra 1	
Length	30
Height	29
Sacral vertebra 2	
Length	30
Height	31
Sacral vertebra 3	
Length	29
Height	30
**Caudal vertebrae**	
Caudal vertebra 1	
Length	28
Height	34
Caudal vertebra 2	
Length	27
Height	36
Caudal vertebra 3	
Length	25
Height	31
Caudal vertebra 4	
Length	26
Height	33
Caudal vertebra 5	
Length	26
Height	34
Caudal vertebra 6	
Length	27
Height	34
Caudal vertebra 7	
Length	26
Height	34
Caudal vertebra 8	
Length	24
Height	31
Caudal vertebra 9	
Length	24
Height	31
Caudal vertebra 10	
Length	25
Height	31
Caudal vertebra 11	
Length	21
Height	31
Caudal vertebra 12	
Length	23
Height	31
Caudal vertebra 13	
Length	23
Height	29
Caudal vertebra 14	
Length	22
Height	32
Caudal vertebra 15	
Length	22
Height	33
Caudal vertebra 16	
Length	20
Height	32
Caudal vertebra 17	
Length	20
Height	29
Caudal vertebra 18	
Length	19
Height	30
Caudal vertebra 19	
Length	19
Height	25
Caudal vertebra 20	
Length	18
Height	27
Caudal vertebra 21	
Length	16
Height	21
Caudal vertebra 22	
Length	13
Height	22
**Neural spines of selected cervical vertebrae**	
Cervical vertebra 3	
Height caudally	12
Length at base	23
Cervical vertebra 21	
Height caudally	24
Length at base	51
Cervical vertebra 29	
Height caudally	34
Length at base	64
Cervical vertebra 37	
Height caudally	51
Length at base	71

**Notes.**

i.c. incomplete

#### Cervical vertebrae and ribs

**Figure 15:**
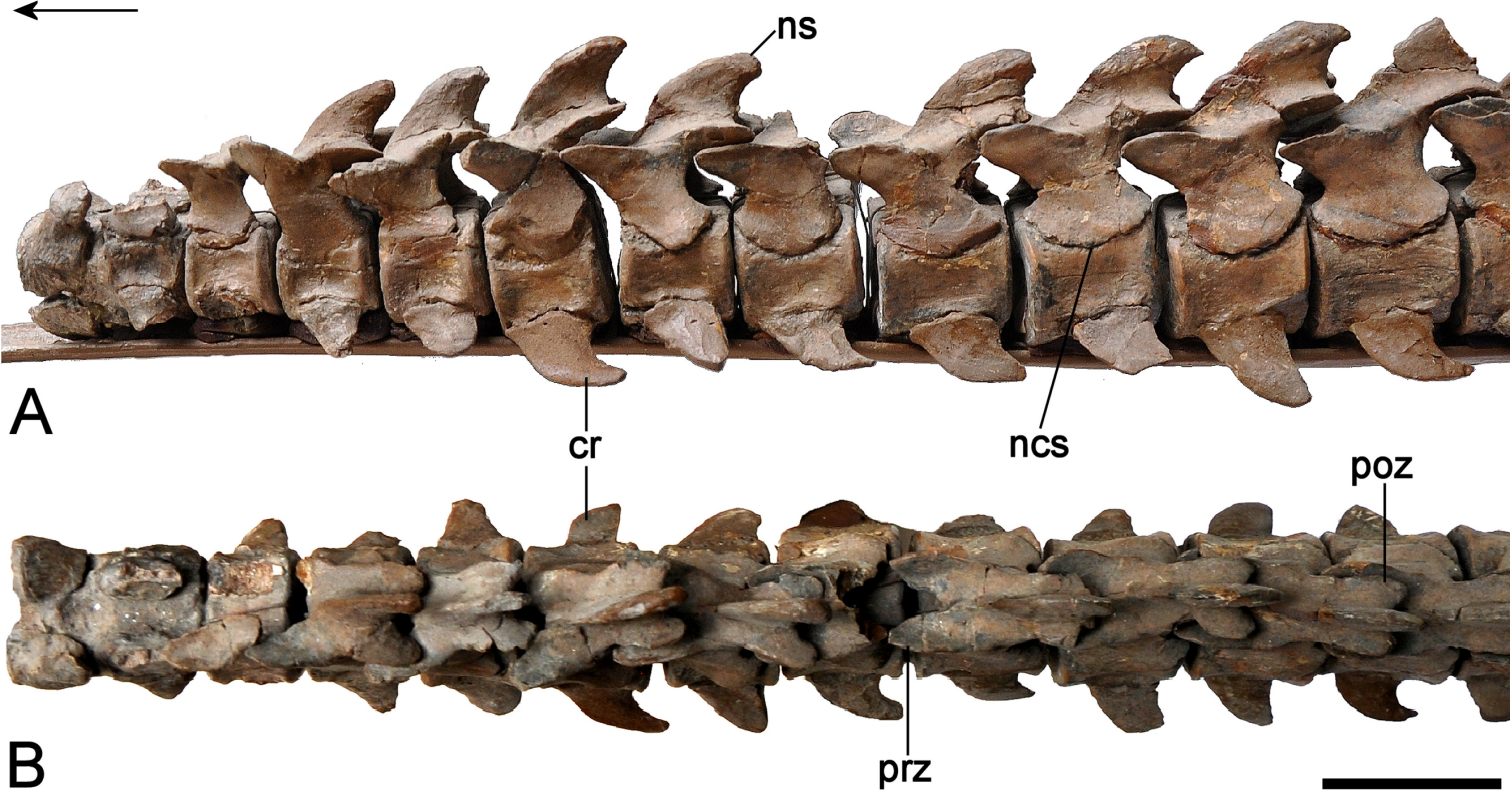
*Brancasaurus brancai*, GPMM A3.B4 (holotype), cranial cervical series. (A) lateral, and (B) dorsal views. Scale bar = 30 mm. The arrow points towards cranial. Abbreviations: cr, cervical rib; ncs, neurocentral suture; ns, neural spine; poz, postzygapophysis; prz, prezygapophysis.

There are 35 cervical vertebrae (*sensu* Sachs, Kear & Everhart, 2013) in addition to the atlas-axis complex (Fig. 15). All lack fusion between the neural arches and centra. The centrum proportions of GPMM A3.B4 are wider than long and high, whereas the length equals the height (see Table 2). In the craniad and mid-cervicals the centra are only slightly wider than long/high (relations in CV 10 = 1:1.14 and in CV 20 = 1:1.15), whereas the width increases relative to the length/height in the caudad cervical vertebrae (relations in CV 37 = 1:1.24). Welles (1952) pointed out that the dimensions of the cervical centra are ontogenetically variable, being proportionately shorter in osteologically immature individuals. All of the articular surfaces are slightly amphicoelous, oval in outline and shallowly concave, deepening sharply towards their centres. In the craniad cervicals the caudal articulation facet is slightly flatter, but becomes more concave (relative to the cranial one) towards the caudal end of the cervical column. The caudal-most cervicals also display a ventrally projecting articular surface rim (also present in the craniad cervicals, see Wegner, 1914, plate 7, Fig. 1A), which corresponds to the “lip” on the cranial articular surface described by Benson et al. (2013a: 246) in *Hastanectes valdensis*. Irrespectively, a prominent central notochordal pit and rounded articular edges are present throughout (but more distinct cranially), and there is also no evidence of ventral notching otherwise indicative of elasmosaurids (see discussion in Sachs, Kear & Everhart, 2013; Sachs & Kear, 2015a; Sachs & Kear, 2015b). Wegner (1914: 257) reported that the notochordal pit in the third–eighth cervical (which is not observable due to the display armature) is 1–3 mm in diameter and deeply inset to about a quarter of the centrum length. By the 16th–18th cervical the notochordal pit is slit-like, becoming circular more caudally.

The lateral surfaces of the centra are weakly craniocaudally concave (Fig. 15A), especially towards the craniad end of the column. They also lack the longitudinal ridges evident in many other long-necked plesiosauromorphs, although this trait is affected by ontogeny (Brown, 1981: 289). A pair of vertical buttresses (about 10 mm long) are present on the last cervical, and extend from the caudodorsal margin of the rib facet towards the neural arch. Each cervical centrum bears a single lateroventrally directed oval rib facet that is centrally placed; these become more circular and caudally situated towards the pectoral region. The cervical ribs are incompletely preserved. The only non-restored example is on the 33rd cervical and measures 93 mm long by 18.7 mm across its base. It is dorsoventrally flattened and weakly expanded at its oval proximal head. The lateroventrally directed rib shaft is tapered, but expands after about a quarter of its length to form a hooked distal extremity with a convex leading edge.

The neural canal floor is surfaced in smooth bone and very slightly concave. The neural arches have been glued to the centra for display. Nevertheless, the ventrally convex neurocentral sutures are clearly discernible throughout the entire column. The neural arch pedicles extend almost the full length of the accompanying centrum and enclose an oval neural canal. The prezygapophyes are craniocaudally longer than the postzygapophyses, but become more equidimensional caudally. Their lateral edges are elevated. Cranially, the prezygapophyses extend over the articular face of the centrum; in contrast the postzygapophyses exceed entirely beyond it. Both the prezygapophyses and postzygapophyes slope at ∼45°and are laterally narrower than the centra (Fig. 15B). The prezygapophyseal articulation facets are flat to slightly concave, oval in outline and conjoined at their bases. They also enclose a narrow cavity that incises the proximal third of the neural spine on each successive vertebra from the 32nd cervical onwards. Similar excavations have elsewhere been identified as ligamentous attachments (Sato, Hasegawa & Manabe, 2006; Sachs & Kear, 2015a). In GPMM A3.B4, a corresponding cavity also occurs between the postzygapophyses from the 29th cervical, but becomes deeper and expands into the distal third of the neural spine in more caudad vertebrae.

**Figure 16:**
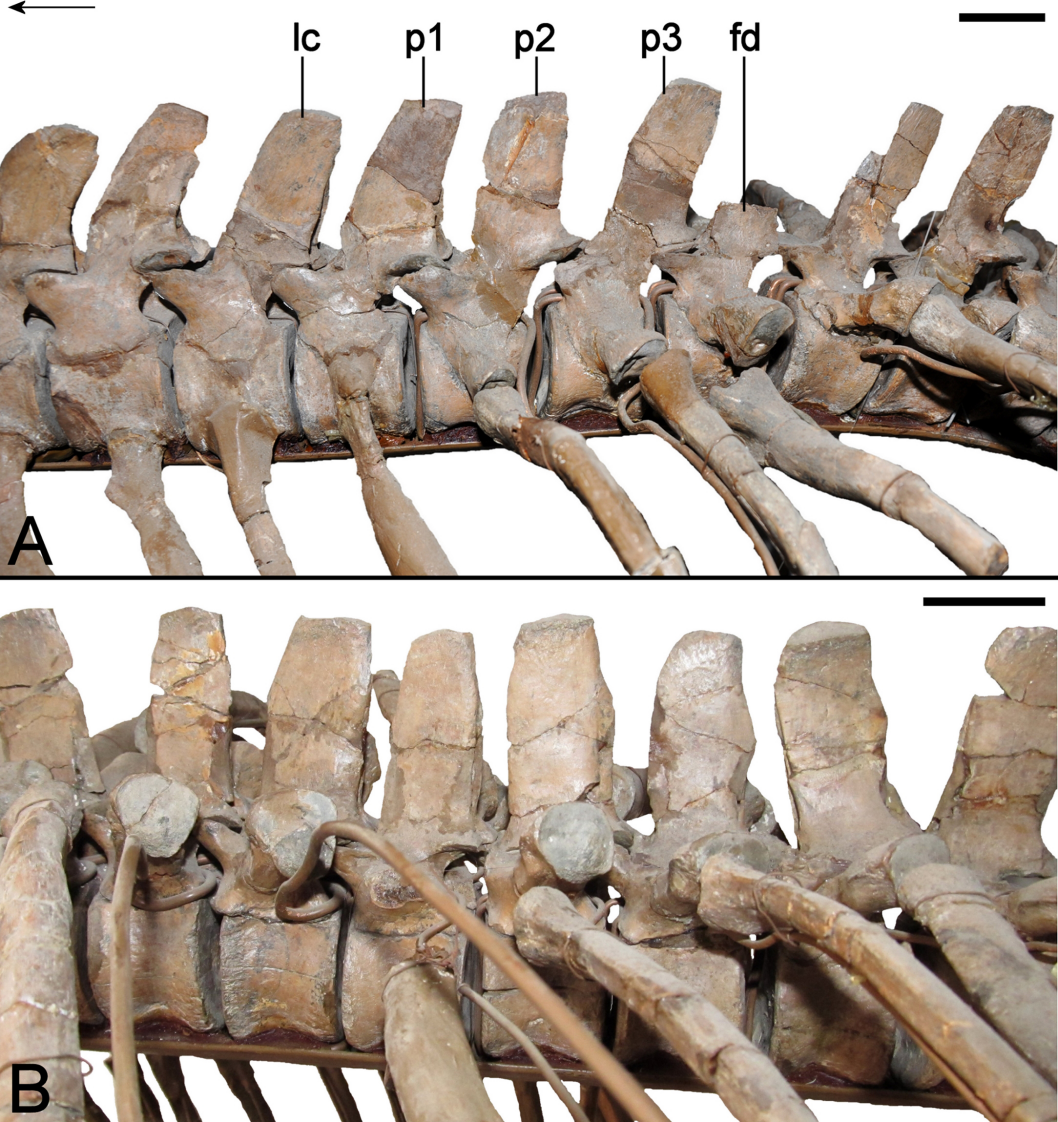
*Brancasaurus brancai*, GPMM A3.B4 (holotype). (A) Cervical-dorsal vertebral transition in lateral view. (B) Dorsal vertebrae in lateral view. Scale bars = 30 mm. The arrow points towards cranial. Abbreviations: fd, first dorsal; lc, last cervical; p1–p3, first, second and third pectoral vertebra.

Components of most cervical neural spines are preserved in GPMM A3.B4 but some have been restored. The original shape is longer than high and distinctly triangular, being caudally arcuate (“shark-fin” like) in the craniad and middle cervical vertebrae, but trending towards rectangular and higher than long in the more caudad cervicals. The leading edges of the neural spines are noticeably convex, with a concave caudal margin and elliptical dorsal apices that are transversely concave and often culminate in a tapered projection caudally. Only the neural spine of the last cervical has straight cranial and caudal edges (Fig. 16A). The swollen apices may also bear a rounded lateral ledge at their base.

The mounting armature obstructs the ventral surfaces of the cervicals. Nevertheless, Wegner (1914: 256) mentioned that the polymorphic sharp to rounded mid-ventral keel progressively broadens (occupying about a third of the transverse width of the centrum) and merges with the rugose edges of the centrum articular facets in the more caudad vertebra. The mid-line keel is also bordered laterally by paired depressions which are deeper in the craniad cervicals. They enclose the foramina subcentralia, which are placed directly adjacent to the mid-ventral keel.

#### Pectoral vertebrae

Three vertebrae (Fig. 16A) conform to the definition of plesiosaurian pectorals proposed by Sachs, Kear & Everhart (2013). They otherwise resemble the terminal cervicals in being shallowly concave and shorter than high with a deep central notochordal pit and rounded articular surface rims. The neural arch pedicles extend laterally to contact the rib facets. The transverse processes are short on the first pectoral (15 mm in maximum transverse width), with approximately one third of the oval rib facet formed by the neural arch. This increases to two-thirds on the second pectoral (24.98 mm in maximum transverse width), and half for the third. Relative concavity of the rib facet also increases along the sequence, as does the height of a buttressing ridge on the dorsal side of the transverse process. The transverse processes on the pectorals are caudolaterally directed. The neural canal is more circular in outline than in the cervicals, and the prezygapophyseal facets are slightly larger than the postzygapophyses. The pectoral neural spines bear compatible cavities vertically incising the cranial and caudal edges; their lateral profile is rectangular and slightly caudally directed. The dorsal apices are weakly expanded to form a shallow concavity. There is no evidence of alternating asymmetry (*sensu* Benson et al., 2013a), and the spine apex on the third pectoral is incomplete.

#### Dorsal vertebrae and ribs

The 19 dorsal vertebrae reassembled in GPMM A3.B4 (Fig. 16B) are all shallowly concave and higher than long (see Table 2). The notochordal pits and prominent rounded rims are reduced, and both the lateral and ventral centrum surfaces exhibit more pronounced concavity than those of the cervicals. The craniad dorsals have elevated transverse processes that are slightly backswept, but decline and become more caudally directed from the 13th dorsal onwards. A blunt keel extends across the ventral side of the transverse processes; this migrates to the leading edge in subsequent dorsals and is paired with another keel on the trailing edge. A shallow depression is formed between these two structures (= subdiapophyseal fossa of Hampe, 2013; see also Benson et al., 2013a). The rib articulations on the transverse processes of the first–third dorsal are oval in shape, but become more circular throughout the mid-dorsal region where they are longitudinally expanded. All of the accompanying ribs are single headed, with shallowly concave proximal articulations. Some ribs posses a medial prong-like process. Blunt keels run along the craniodorsal edge behind the articular head, but there is no distinctive adjacent concavity as reported in *Leptocleidus superstes* (Kear & Barrett, 2011). Both the pectoral and dorsal ribs are otherwise circular in cross-section and transversely compressed at their distal extremities.

The dorsal neural canals are triangular in outline with intermittent small nutritive foramina perforating the lateral centrum sides. The dorsal zygapophyses resemble those of the pectorals, and the neural spines likewise bear concavities on their leading and trailing edges (the later being larger and more prominent). In lateral view, the neural spine profile is rectangular and clearly higher than the accompanying centrum (see Wegner, 1914: 266–267, Table 2; this is in contrast with Benson et al., 2013a, who implied that the neural spine height was equal to that of the centrum). The spine apices are transversely narrow, elongate and straight. Benson et al. (2013a: 239; p. 240, Fig. 4E) stated that: “*Brancasaurus* also show(s) alternating asymmetry in the outline of the anterior (craniad) dorsal neural spines in dorsal view.” This is not consistent, and several dorsal neural spines are either incomplete or missing. Moreover, the entire post-pectoral vertebral column has been reconstructed (Wegner, 1914) and might not be precisely articulated or sequenced as in life.

#### Sacral vertebrae and ribs

**Figure 17:**
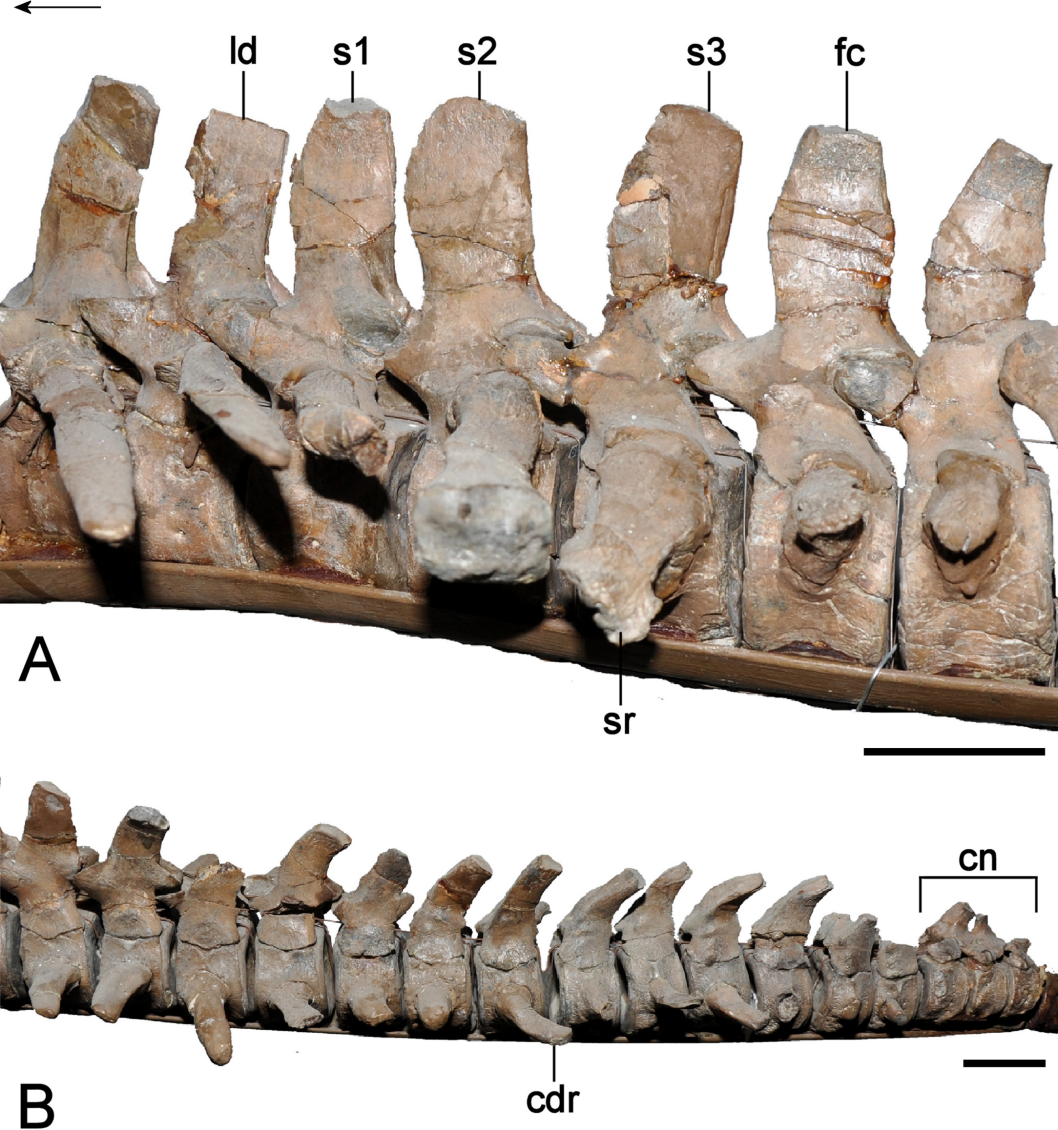
*Brancasaurus brancai*, GPMM A3.B4 (holotype). (A) Sacral vertebrae in lateral view. (B) Terminal caudal series in lateral view. Scale bars = 30 mm. The arrow points towards cranial. Abbreviations: cdr, caudal rib; cn,coossified neural arches; fc, first caudal vertebra; ld, last dorsal vertebra; s1–s3, first, second and third sacral vertebra; sr, sacral rib.

The three identifiable sacral vertebrae are all shallowly concave and marginally higher than long (Fig. 17A). The centrum articular faces resemble those of the dorsals. The first sacral according to Wegner (1914) is intermediate in morphology between the dorsal and sacral series (characterised by the second and third sacral vertebrae). Its zygapophyses are approximately equal in length, unlike the second sacral in which the postzygapophyses are somewhat longer (zygapophyseal facets are obscured in the third sacral). Like the dorsals, the sacral neural spines are rectangular, straight and have prominent cavities along their edges. The dorsal apices are also only weakly expanded and the neural canal is triangular in outline.

The first sacral rib facets are situated on the neural arch, with only their basal edges contacting the centrum. They are also buttressed ventrally by a vertical ridge. The first sacral rib is noticeably smaller and more caudolaterally directed than those of the succeeding vertebrae. Its shaft is oval in cross-section (incorporating a caudolaterally projecting flange on the right-hand side) and the distal ilial facet is sub-circular. Conversely, the rib articulation on the second sacral has a lesser contribution from the neural arch (about two-thirds of its height); this further decreases in the third sacral where the neural arch forms only the dorsal margin of the facet. The second sacral rib is the longest of the three, and is slightly caudally directed. In contrast, the third sacral rib is lateroventrally oriented. The left-hand second sacral rib shaft is slender but expands towards its ends; the right-hand rib is asymmetrical and appears to be diagenetically distorted. The proximal articulations on the left second and third sacral ribs are dorsoventrally elongate, convex, and rectangular in outline (this is oval in the malformed right rib). Opposing ridges extend along both the craniodorsal and caudad edges.

#### Caudal vertebrae and ribs

Wegner (1914: 273) listed 25 caudal vertebrae, of which 22 are still preserved. All of the caudal centra are higher than long and have shallowly concave articulation facets that deepen towards their centres (Fig. 17B). The proximal-most two caudals have a minor contact of the neural arch with the lateral rib facets. From the third caudal onwards, the rib facets are situated from the dorsolateral to mid-level of the centrum. They are oval in the first two caudals but become more circular in the succeeding vertebrae. The caudal ribs are mostly restored, although a complete example attached to the fifth vertebra is dorsoventrally compressed, triangular in outline and caudolaterally tapered. The proximal rib head is circular with narrow keels extending along the cranial and caudal edges of the shaft.

The caudal neural spines are rectangular in lateral view, trending to low and recurved from the eighth caudal; there are no obvious cavities along the leading and trailing edges. The dorsal apices are elongate and oval to sub-circular distally. The zygapophyses are not well preserved but the prezygapophyses are slightly larger than the postzygapophyses in at least the first caudal. The neural canal is sub-triangular and some caudals exhibit foramina on the lateroventral margins of the centra.

Wegner (1914: 274, Fig. 8) noted that the distal-most neural arches, of which only the bases are still preserved, were co-ossified to form a ‘pygostyle-like’ structure at the tail tip (*sensu* Kear, Schroeder & Lee, 2006).

#### Gastral ribs

Wegner (1914: 280) reported that 10 rows of gastralia and remnants of 37 separate elements were initially preserved. In the present mount, there are four symmetrically arched gastralia. They taper laterally and have a concave dorsal, and ventromedially inclined ventral side incised by a deep furrow. Remnants of 18 strap-shaped lateral elements are also present. These have a circular cross-section and bear tapering ventral and dorsal ends.

### Appendicular skeleton

#### Interclavicle and clavicles

**Figure 18:**
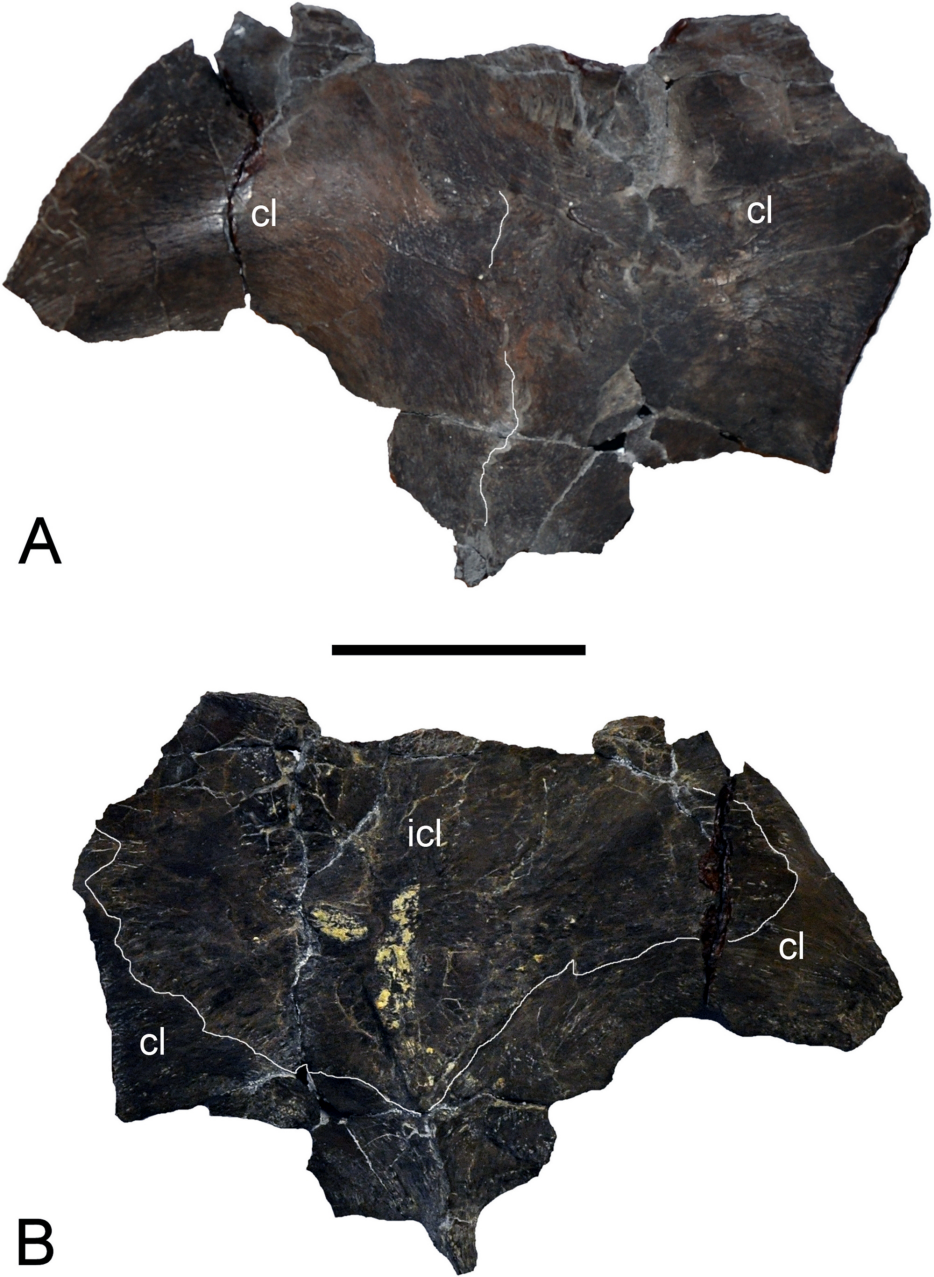
*Brancasaurus brancai*, GPMM A3.B4 (holotype), clavicle-interclavical complex. (A) Ventral, and (B) dorsal views. Scale bar = 50 mm. Abbreviations: cl, clavicle; icl, interclavicle.

The complete interclavicle was originally preserved in articulation with the clavicles (Wegner, 1914, plate 9, Fig. 1). A fully fused plate-like bone complex stored in the GPMM collection likely represents the remainders of these elements (Fig. 18). Based on this fossil and Wegner’s (1914) description, we reconstruct the cranial edge of the interclavicle as being widely embayed and bordered by oblique sutures for the clavicles. A rounded midline projection is present caudally, and the ventral surface bore a deep cleft bordered by lateral convexities, which are evident on the GPMM specimens; the dorsal surface is concave and smooth.

Wegner (1914, plate 9, Fig. 1) showed the clavicles to be overlain by the interclavicle. His brief discussion also reported participation of the clavicles in the craniomedial fenestra (termed the “foramen interscapulae” by Wegner, 1914: 279) and that they failed to meet along the midline. There is no evidence of this in the specimen.

#### Scapulae

**Figure 19:**
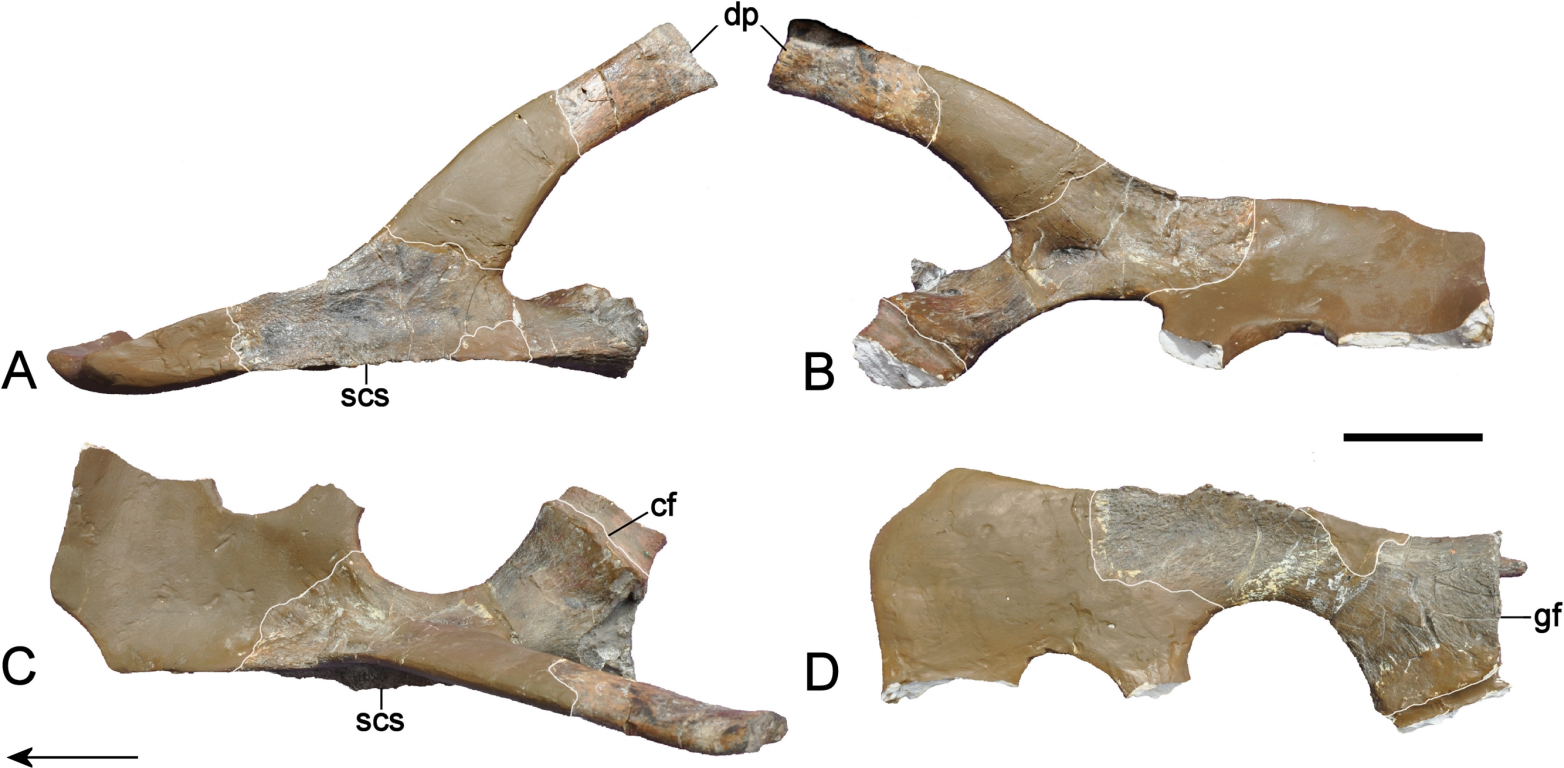
*Brancasaurus brancai*, GPMM A3.B4 (holotype), detailed photos of left scapula that is assembled with the coracoid in mounted skeleton. (A) Lateral, (B) medial, (C) dorsal, and (D) ventral views. Scale bar = 50 mm. The arrow points towards cranial. Abbreviations: cf, coracoid facet; dp, dorsal process; gf, glenoid facet; scs, scapular shelf.

Originally both scapulae included large sections of the dorsal processes and ventral plates. However, the pectoral girdle was restored from numerous pieces and some features such as the craniomedial process (labelled “y” in Wegner, 1914, plate 9, Fig. 1) are of uncertain veracity (see Wegner, 1914: 279). As preserved today, the scapulae comprise fragments of the glenoid regions, ventral plates and dorsal processes (Fig. 19). The more complete left scapula measures 135 mm in reconstructed length; a 79 mm long section of the right scapula incorporates the bases of both the glenoid and dorsal processes. The glenoid and coracoid facets are approximately equal in length and triangular in outline. The coracoid facet tapers medially, whereas the glenoid facet is craniolaterally directed. The articulation surface on the left scapula is coated in plaster, but the glenoid is clearly concave and rugose for cartilage attachment. The lateral scapular shelf is prominent and extends from the glenoid facet cranially along the entire length of the bone. The glenoid ramus is 586.1 mm long and concave dorsally. The dorsal process arises 44 mm from the glenoid facet. It is rod-like and inclined caudally by about 40°(see Wegner, 1914: 279). The cranial edge is produced into a low ridge; the caudal edge is rounded. The distal apex of the dorsal process is transversely compressed and slightly tapered, but the end is missing. The medial edge of the ventral plate is produced into a shelf that runs from the coracoid facet to border the pectoral fenestra; craniolaterally there was apparently also a small facet (Wegner, 1914: 279), possibly for an interscapular cartilage. The ventral surface is flat to concave beneath the base of the dorsal process.

**Figure 20:**
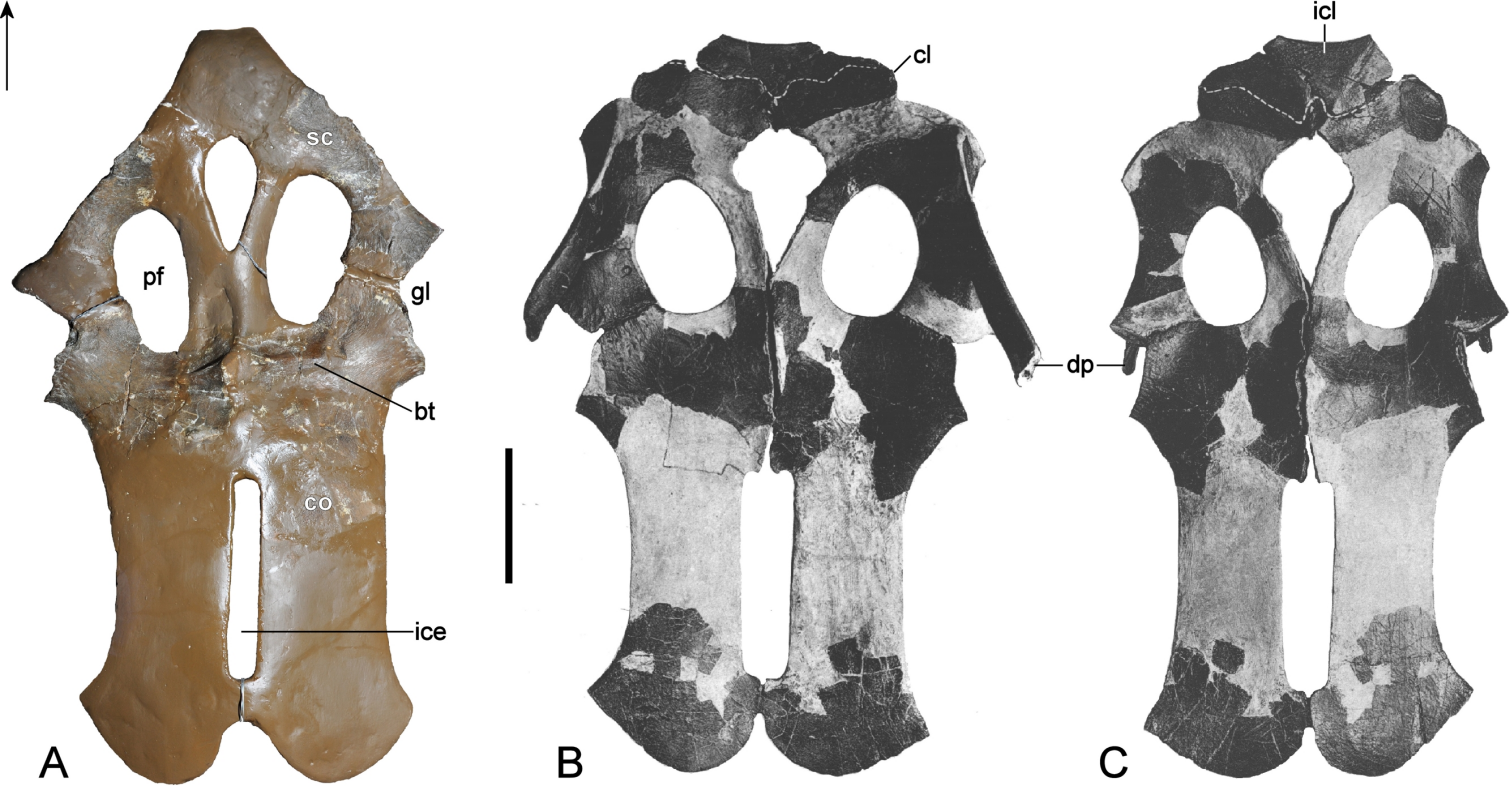
*Brancasaurus brancai*, GPMM A3.B4 (holotype), pectoral girdle. (A) As currently mounted in ventral view, and (B, C) as depicted by Wegner (1914) in (B) dorsal, and (C) ventral views. Scale bar = 100 mm. The arrow points towards cranial. Abbreviations: bt, buttress-like structure; cl, clavicle; co, coracoid; dp, dorsal process; gl, glenoid; ice, intercoracoid embayment; icl, interclavicle; pf, pectoral fenestra; sc, scapula.

#### Coracoids

Only the glenoid and inter-coracoid regions of both coracoids are preserved (Fig. 20). The glenoid facet (52 mm in maximum diameter) exceeds the scapular facet (42 mm in maximum diameter) in size. The triangular surface of the glenoid facet is visible in lateral view. It is caudolaterally tapered, concave and heavily pitted for the attachment of cartilage. The craniomedial edge of the coracoid is dorsoventrally flattened and seems to have projected anteriorly to frame the pectoral fenestra (Wegner, 1914, plate 9, Fig. 1; see also Figs. 20B and 20C). The coracoids are deepest at the medial intercoracoid symphysis where they become vaulted dorsally and ventrally project as a midline process, although this is restored in plaster and was separated along the intercoracoid contact in Wegner’s figure (Wegner, 1914, plate 9, Fig. 1). Prominent transverse butresses run across the ventral surfaces of the coracoids from the symphysis towards the tip of the scapular facet. Less prominent buttresses are also present at the same level on the dorsal side. The cranial edges of both coracoids are incomplete and coated in plaster.

The caudal extremities of the coracoids were depicted by Wegner (1914, plate 9, Fig. 1) with caudolateral cornua. Brown (1981) reported that development of these structures was ontogenetically influenced, thus they might have been larger in an osteological mature individual. The rounded caudomedial edges of the coracoids in Wegner’s (1914) photograph are also scalloped along the caudal-most margin of the intercoracoid facet (right coracoid), and at their distal mid-line contact. The coracoids thus formed an opening that seemingly equates with an intercoracoid embayment (see Figs. 20B and 20C). Wegner (1914: 279) furthermore reported that a facet was present in the caudal margins on the coracoids. Ketchum & Benson (2010: 380–381) stated: “[o]ne reason that O’Keefe (2001) recovered *Brancasaurus* as an elasmosaurid may be that a posterior intracoracoid embayment (149.1; an elasmosaurid synapomorphy) was scored as present in his dataset. Wegner (1914) could not distinguish between the presence of this structure and the presence of round holes adjacent to the median margin of the coracoid (152.1) based on preserved materials of *Brancasaurus*.” Wegner (1914: 280), however, did not explicitly refer to either of these structures; rather he only mentioned that the craniomedial margins were potentially curved (labled “x” in Wegner, 1914, plate 9, Fig. 1A). Conversely, our observations found no original remnants of the distal parts of the coracoids, therefore any character state reconstructions of this feature are presently speculative (see discussion).

#### Humeri

**Figure 21:**
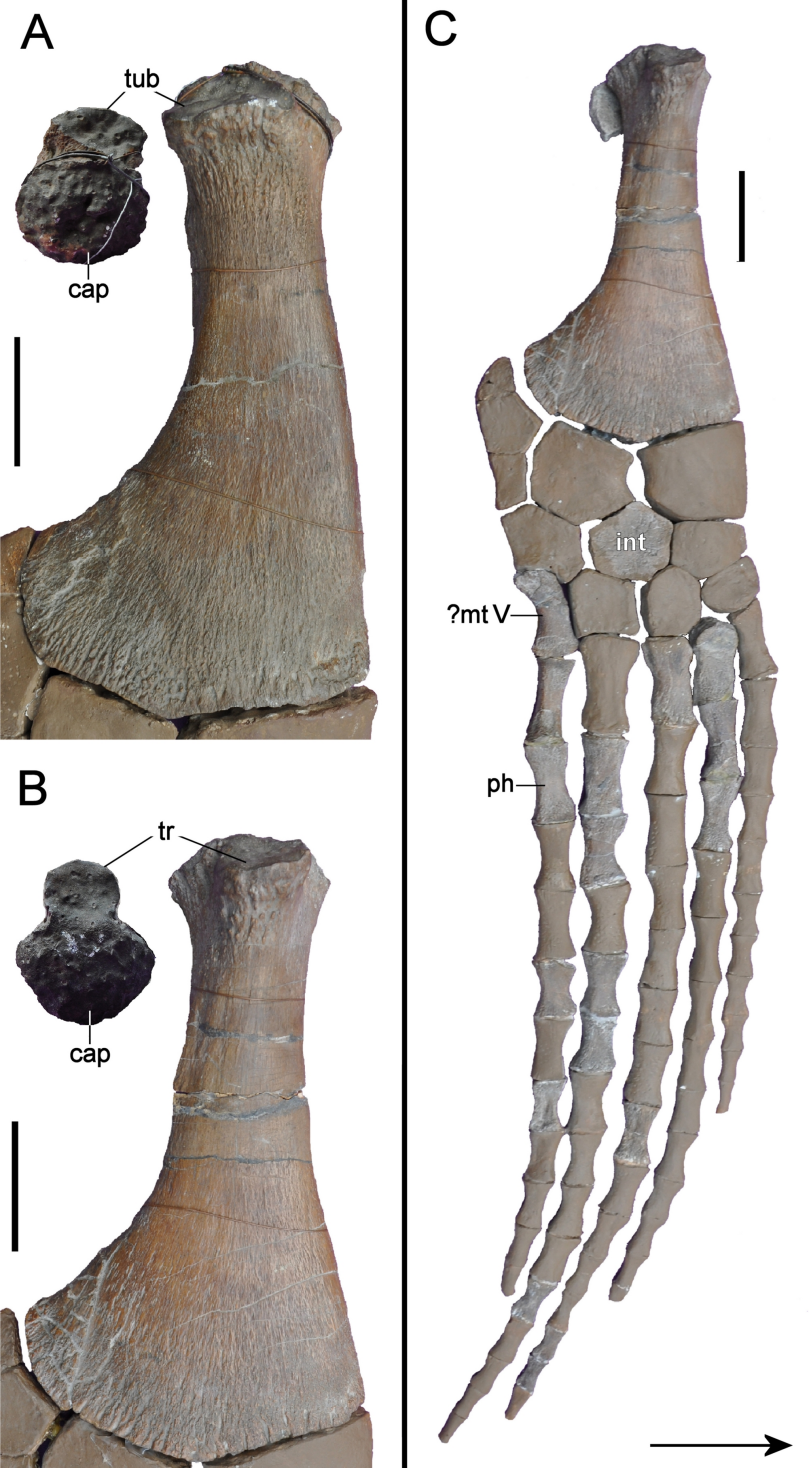
*Brancasaurus brancai*, GPMM A3.B4 (holotype), appendicular elements. (A) Right humerus in dorsal and articular views. (B) Right femur in dorsal and articular views, and (C) Right pelvic limb in dorsal view. Scale bars = 50 mm. The arrow points towards cranial. Abbreviations: cap, capitulum; int, intermedium; mt V, possible metatarsal V; ph, phalange; tr, trochanter; tub, tuberosity.

Both humeri are preserved. They are slightly longer and more robust than the recovered right femur (Table 3) and also exhibit a greater degree of curvature along their caudad edge (Fig. 21A). Their maximum width is about half the length (ratio 1:1.9; see Table 3). Their proximal heads are expanded to form a hemispherical capitulum, which is rugose indicating a cartilage cap. The dorsal tuberosity is oval in outline and separated from the capitulum by a distinct groove. The craniad margins of both humeri are slightly proximodistally convex and weakly sigmoidal. The caudad margins, on the other hand, are convex proximally trending towards caudodistally curved. The humeral shafts are oval in cross-section. The distal extremities are dorsoventrally flattened and bear distinct rugose facets for the radius, ulna and a supernumerary ossification. The radial facet is longest (74 mm in maximum diameter on the right humerus) and caudodistally offset. Its edges are straight as opposed to the ulnar facet, which is concave and 49 mm in maximum diameter and caudoproximally offset. The caudad-most supernumerary facet is convex and 50 mm in maximum diameter. The intersections between each of the distal facets are raised into prominent ridges.

**Table 3:** Measurements (mm) of the appendicular skeleton of *Brancasaurus brancai* (GPMM A3.B4).

**Coracoid**	
Width transversely	274
Height of glenoid facet	42 (left)
Preserved length of glenoid facet	92 (left)
Height of glenoid facet	44 (right)
Preserved length of glenoid facet	96 (right)
**Scapula**	
Height of dorsal process at caudal edge	100
Width of dorsal process at base	61
Width at preserved dorsal end of dorsal process	279
Transverse width of articular end	73
Height of articular end	36
**Humerus (left)**	
Length	241
Distal length craniocaudally	127
Proximal height dorsoventrally	74
Proximal width craniocaudally	58
**Humerus (right)**	
Length	230
Distal length craniocaudally	130
Proximal height dorsoventrally	73
Proximal width craniocaudally	62
**Ilium (left)**	
Height dorsoventrally	113
Width of dorsal end	43
Diameter of ventral end	44
**Ilium (right)**	
Height dorsoventrally	121
Width of dorsal end	39
Diameter of ventral end	38
**Ischium (left)**	
Greatest length (medially)	152
Width transversely	129
Height of acetabular facet	38
Length of acetabular facet	73
**Ischium (right)**	
Greatest length (medially)	139
Width transversely	140
Height of acetabular facet	38
Length of acetabular facet	64
**Pubis (left)**	
Transverse width	191
Craniocaudal length	167
Height of acetabular facet	35
Width of acetabular facet	79
**Pubis (right)**	
Transverse width	177
Craniocaudal length	157
Height of acetabular facet	32
Width of acetabular facet	80
**Femur (right)**	
Length	215
Distal length craniocaudally	124
Proximal height dorsoventrally	58
Proximal length craniocaudally	68

#### Pubes

**Figure 22:**
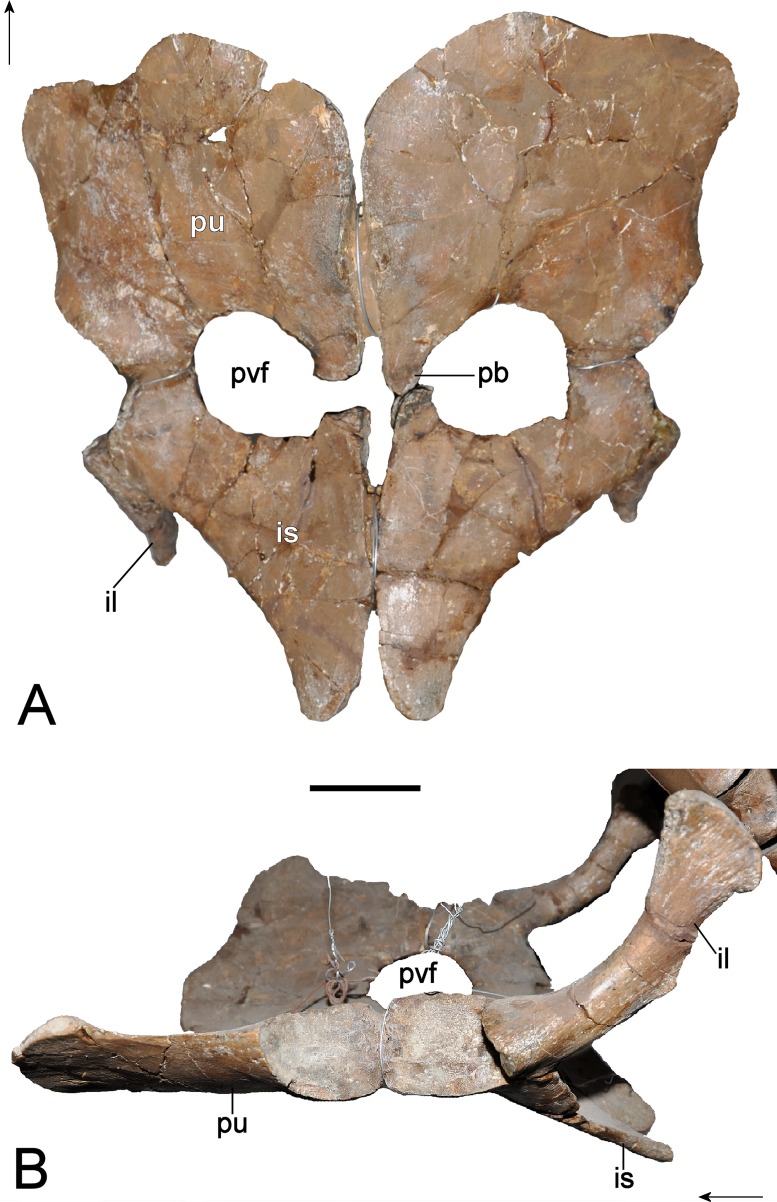
*Brancasaurus brancai*, GPMM A3.B4 (holotype), pelvic girdle as currently mounted for display. (A) ventral; and (B) lateral views. Scale bar = 50 mm. The arrows point towards cranial. Abbreviations: il, ilium; is, ischium; pb, pelvic bar; pu, pubis; pvf, pelvic fenestra.

The pelvic girdle comprises considerable original bone. The pubes are sub-rectangular in outline and slightly dished (Fig. 22). The acetabular facets (58 mm maximum diameter) exceed the ischial facets in length (32 mm maximum diameter). In lateral view the acetabular facet is triangular in outline and tapered towards its craniad extremity. Its articular surface is concave and rugose. The lateral edge is 62 mm long, and weakly concave where it extends to the craniolaterally directed pubic cornu (27 mm in maximum width). The craniomedial margin is convex. The medial symphysis is rugose, thickened and apparently incorporated a substantial space between the adjacent elements. The caudomedial extremity of the pubis is thickened and projects to meet the ischium in the pelvic bar. The laterally curved edges of both pubes and ischia (orginally more complete, see Wegner, 1914, pl. 9, Fig. 2) indicate that a rhombic space was present in the centre of the pelvic bar, similar to the condition in the elasmosaurid *Futabasaurus suzukii* Sato, Hasegawa & Manabe, 2006 (see Sato, Hasegawa & Manabe, 2006, Fig. 2). The broadly embayed medial margin of the pubis contributes to the cranial half of the pelvic fenestra.

#### Ischia

The ischia are classically plesiosauroid-like (*sensu* Brown, 1981) with their laterally positioned articular heads separated from the proportionately craniocaudally short (Table 3) medial blade by a stout shaft. Most of the ischial body is dorsoventrally flat and plate-like. The comparatively thicker articular head comprises an elongate caudodorsally offset acetabular facet (52 mm in maximum length) separated from the pubic facet (32 mm in maximum length) by a low ridge. The articular surfaces are deeply pitted and irregular indicating the presence of cartilage in life. The craniad margin of each ischium is broadly embayed where it borders the pelvic fenestra; medially paired projections extend forward to contact the pubes along the pelvic bar (this is broken on the left-hand ischium but was originally in contact). The dorsal surfaces of the ischia are slightly convex adjacent to the symphysis. Laterally the inter-ischium contact is concave in ventral view. The caudad-most extremities are rounded and diverge around a triangular space, which was again likely occupied by cartilage.

#### Ilia

The ilia are rod-like with a caudad curvature in lateral view. The ventral articulation is expanded and accommodates the concave acetabular and ischial facets; their articulation surfaces are triangular and smooth. A low ridge arises ventrolaterally and extends to the mid-level of the shaft. The convex caudad edge of the iliac shaft bears a blunt mid-line tubercle, approximately 14 mm long. The dorsal end of the ilium is transversely compressed and fan-like; its apex is gently convex and smooth. The lateral and medial surfaces are rounded trending towards flat dorsally.

#### Femur

Only the right femur is present in the mounted skeleton (Figs. 21B and 21C). Its length is almost twice its width (ratio 1:1.8; see Table 3). The proximal articular head is expanded and exceeds the width of the shaft. The capitulum is hemispherical and heavily pitted for cartilage attachment. The dorsal trochanter is about half the width of the capitulum and situated slightly distally along the shaft; its rugose proximal surface is separated from the capitulum by a shallow groove. The leading edge of the femoral shaft is proximodistally concave, whereas the trailing edge is almost straight becoming strongly curved distally. The mid-shaft cross-section is oval, and a low process is present 110 mm along the caudad margin.

The distal facets for the tibia, fibula and supernumerary ossification all have concave and rugose articular surfaces. The elongate tibia facet (63 mm in maximum diameter) is caudodistally offset while the shorter fibula facet (44 mm in maximum diameter) is caudoproximally offset. Both are separated by a low vertical ridge. In contrast, the caudad-most supernumerary facet (35 mm maximum diameter) merges smoothly with the fibular facet but is inflected medially with a convex tapered edge.

#### Epipodials

There are no epipodials retained with the mounted skeleton. However, Wegner (1914: 286) mentioned that a radius, tibia and fibula were originally preserved. The radius was similar to the tibia, except in its smaller dimensions and straighter articular edges. The tibia was alternatively more rounded with an epipodial foramen present between the tibia and fibula.

#### Mesopodials and phalanges

Wegner (1914: 285) stated that the distal limb bones were completely disarticulated and not all were collected. Indeed, only fourteen mesopodials are now retained in GPMM A3.B4. One hexagonal element restored in the right hindlimb probably represents the intermedium; the others cannot be identified with certainty.

All of the 14 original phalanges are proximodistally elongate and constricted at their midsection to form an hourglass shape. They vary in length from 20–52 mm and likely derive from both the fore- and hindlimbs.

### Possible soft tissue and ingested material

Wegner (1914) and Wegner (1926) documented a 0.5–1.5 mm thick sheet of fibrous to granular “sparitic calcite” that encased the articulated pectoral region of GPMM A3.B4. This is now lost but the same unusual matrix also covered a 600 mm^2^ area around the excavation site, and encased the entire skeleton according to quarry workers. Wegner (1914) and Wegner (1926) described the surface texture of this material as gently undulating but otherwise smooth, with a cross-section comprising thin internal and external lamellae enclosing a thicker medial layer. Wegner (1914) and Wegner (1926) argued that the continuity of diagenetic cracks extending from the surrounding sediment negated the possibility of calcite precipitation from percolating groundwater. Rather, he interpreted the deposits as “skin replacement,” formed during protracted degradation of the dermis. We obviously cannot confirm this hypothesis, but note that calcium phosphate soft tissue mineralization has been linked to lithification of chemoautotrophic microbial mats in anoxic Ca-rich microenvironments (Iniesto et al., 2013) like those of the Isterberg Formation.

In addition, Wegner (1914) and Wegner (1926) mentioned an unusually coarse-grained sediment accumulation (140 × 150 × 20 mm) aligned in close proximity with dorsal vertebrae 11–14. This was completely removed during preparation but apparently comprised medium-sized granules and small pebbles cemented in a coarse-grained quartzose sand with abraded bone fragments. The sediment surface adhering to the left wall of the body cavity was also irregularly folded while the exposed layer was smooth. Ribs from the left side of the body were deeply impressed into this sandy matrix. Wegner (1914) and Wegner (1926) therefore surmised that this material must have been allochthonous because it did not match the surrounding sediment. Moreover, he proposed interpretation as a lithified mass of ingested unconsolidated sand and food remains (bones) from the gastrointestinal tract. Wegner (1914) and Wegner (1926) used the term “gastrolith” to describe this residue, however if correct, it would be more accurately identified as an accumulation of gastroliths (Wings, 2007) and demalites (bone fragments, Hunt & Lucas, 2012). As a whole, this assemblage should be considered a bromalite (Hunt, 1992; Hasiotis et al., 2007). In the original definition this term comprised all “matter” (Hunt, 1992)—“organic and inorganic” (Hasiotis et al., 2007)—which entered an organism orally and has been expelled or retained inside the body. Hunt & Lucas (2012) alternatively modified their definition to encompass organic matter (food) only, thus excluding gastroliths. However, we recommend here to apply the original definition, as multicomponent accumulations of food remains and gastroliths are common occurrences (see e.g., Bartholomäus et al., 2004; McHenry, Cook & Wroe, 2005 for examples in plesiosaurians).

## Discussion

### Synonymy of *Brancasaurus brancai* and *Gronausaurus wegneri*

**Figure 23:**
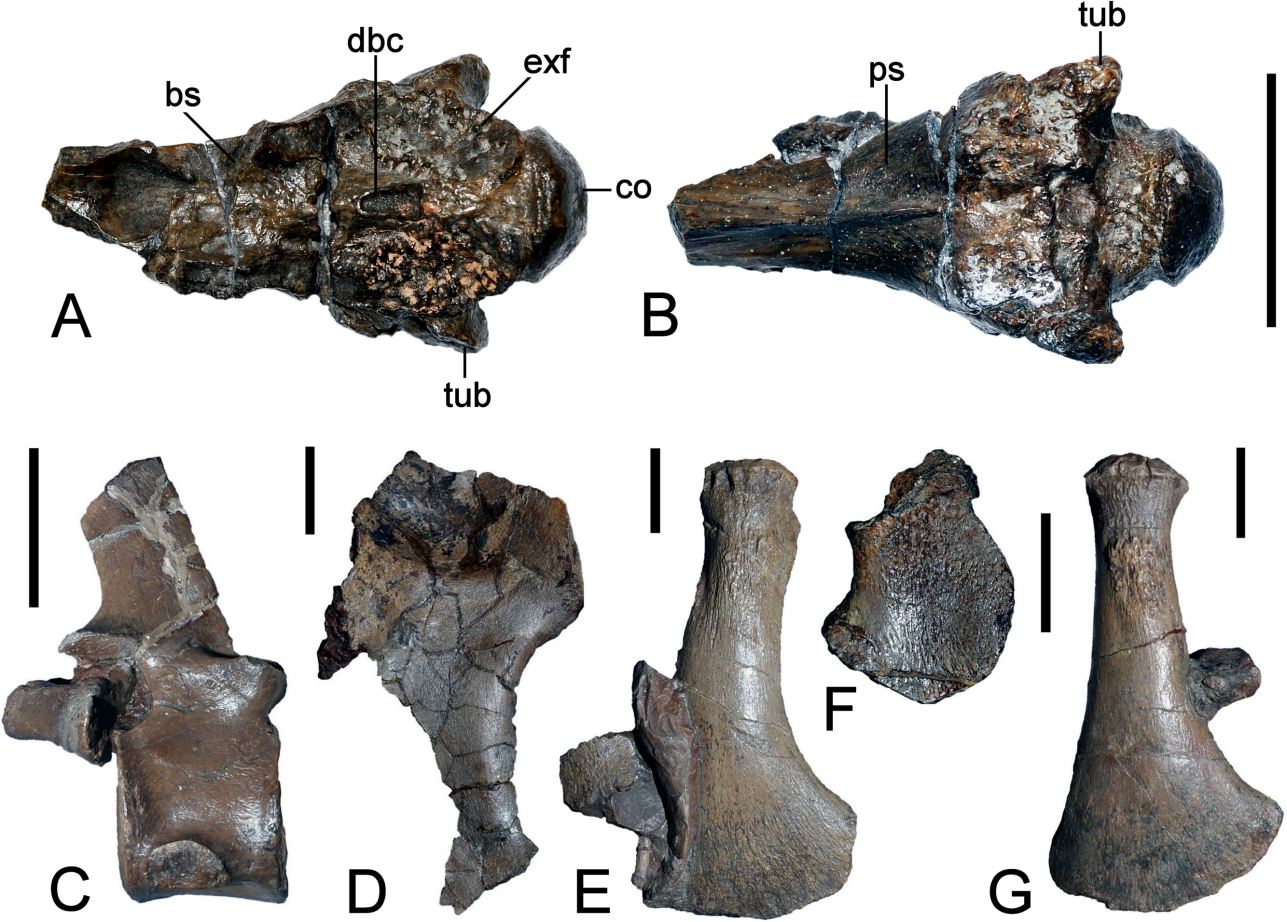
*Brancasaurus brancai*, referred specimen GPMM A3. B2 (holotype of *Gronausaurus wegneri* Hampe, 2013), Isterberg Formation, Gronau (Westfalen). (A, B) Basioccipital and basisphenoid-parasphenoid complex in (A) dorsal, and (B) ventral views; (C) cervical vertebra in lateral view; (D) left coracoid in ventral view; (E) right humerus in ventral view; (F) fibula in ventral view; (G) right femur in ventral view. Scale bars in (A) = 30 mm; (C–G) = 50 mm. Abbreviations: bs, basisphenoid; co, condylus occipitalis; dbc, depression in basioccipital; exf, facet to exoccipital-opisthotic and prootic; tub, tubera.

The GPMM A3.B4 holotype individual of *Brancasaurus brancai* shows closest osteological and stratigraphical compatibility with the skeletal remains of *Gronausaurus wegneri* (Fig. 23), also recovered from the upper Bückeberg Group (albeit eight metres higher in the sequence) at the Gerdemann & Co. clay-pit (Hampe, 2013). *Gronausaurus wegneri* has been classified as a leptocleidid (Hampe, 2013) or basal elasmosaurid (Benson & Druckenmiller, 2014). The only known specimen of this taxon (GPMM A3.B2) is an incomplete postcranial skeleton of an osteologically mature individual with referred cranial fragments (Wegner, 1914: 237 mentioned vertebrae and a few other bones). Circumstances surrounding the discovery of these remains are unknown (M Bertling, pers. comm., 2012), but Hampe (2013) stated that the isolated cranial components (previously stored in the GPMM bulk collection but now labelled as GPMM A3.B2) can be referred to *G. wegneri* because they were seemingly distinguishable from *B. brancai*. In contrast, our first-hand inspection of these fossils revealed close trait compatibility. For example, the parasphenoid underlaps the basioccipital in both *B. brancai* and *G. wegneri* (Wegner, 1914; Hampe, 2013). Wegner (1914) additionally depicted a ventrolateral flange adjacent to the basioccipital tuber in *B. brancai* (23A and 23B). Hampe (2013) considered this feature to be a key difference with *G. wegneri*, but it is no longer visible in the restored GPMM A3.B4 skull. Furthermore, a clearly delimited lateral parasphenoid flange underlies the right basioccipital tuber in GPMM A3.B2. The basioccipital tubera are otherwise heavily abraded, and in our opinion, their abbreviated extent does not separate *G. wegneri* from *B. brancai* (*sensu* Hampe, 2013: 489).

The “cochlear facet” identified by Hampe (2013: 475) was illustrated in Wegner’s (1914) drawing of *B. brancai* (Fig. 12C). The notochordal pit, on the other hand, was not easily discernible in Wegner’s (1914) depiction; however, it is actually present high on the occipital condyle (contra Hampe, 2013; Fig. 7A). In addition, Hampe (2013) emphasized that the absence of a condylar groove differentiated *B. brancai* from *G. wegneri*, but this structure is insipiently expressed in *B. brancai* as a shallow circumscribing indentation (Fig. 7A).

Hampe (2013) listed additional cranial specimens (dentigerous bone fragments, parts of the parietals and squamosals). Components of the pterygoids and vomers were also stored with these elements in the GPMM collection, but have not been included in in Hampe’s (2013) study. All of these specimens are morphologically identical to the equivalent bones in *B. brancai*.

Hampe (2013: 475) described the articular surfaces on the cervical centra of *G. wegneri* as “procoelous”—the caudal face being flattened relative to the cranial face. In contrast, we found the centrum articular surfaces of *G. wegneri* to be polymorphic and platycoelous to shallowly amphicoelous as in *B. brancai* (this is also an ontogenetically variable trait: see Brown, 1981). *Gronausaurus wegneri* also reportedly had four pectoral vertebrae whereas *B. brancai* has only three; nevertheless, the count in *G. wegneri* was estimated from rib facet positions because the neural arches were fully fused (Hampe, 2013: 478).

Another defining trait of *G. wegneri* was the “presence of a subdiapophyseal fossa below the transverse processes on the second–fourth pectoral and anterior dorsal vertebrae” (Hampe, 2013: 475). Comparable structures likewise occur on the dorsals of *B. brancai*. Supernumerary facets on the propodials are also absent. Other features such as the “sharply bent” ilia (Hampe, 2013: 489) and elongate humerus relative to the femur (see Table 3) are also indistinguishable. However, noticeable differences include the proportionately shorter ischia (Hampe, 2013 measured this relative to the pubis, which is ∼20% longer in *B. brancai*: ischium/pubis length = 152/167 versus 175/153 in *G. wegneri*) and epipodials that are wider than long (see Wegner, 1914: 286); although the radius and fibula of *G. wegneri* are damaged and the ulna is equidimensional (see Hampe, 2013: 488). Differences are also evident in the dorsal/sacral vertebral counts (19/3 in *B. brancai*, 17/4 in *G. wegneri*, note that Hampe’s (2013) fourth pectoral is in fact the first dorsal according to the definition of Sachs, Kear & Everhart, 2013). However, Brown (1981) and Großmann (2007, Table 2) demonstrated that the number of vertebrae can vary within one plesiosaurian species (e.g., *Seeleyosaurus guilelmiimperatoris* shows 16–19 dorsals and 2–3 sacrals or *Microcleidus* (*Hydrorion*) *brachypterygius* 14–20 dorsals and 2–3 sacrals).

Hampe (2013) also stated that there is no evidence for a pelvic bar, however, the preservation does not permit this statement and the right pubis (Hampe, 2013, Fig. 7A) shows a caudomedial process, which is an indicator for a pelvic bar. Finally, Hampe (2013) stated that distal-most caudal centra of *G. wegneri* were not fused; this is compatible with *B. brancai*, which only exhibits co-ossification of the terminal neural arches.

**Table 4:** Conflicting character scores between the holotype specimens of *Brancasaurus brancai* (GPMM A3.B4) and *Gronausaurus wegeri* (GPMM A3.B2).

Dataset	Character	Scores recorded in this analysis	
		*Brancasaurus brancai*	*Gronausaurus wegeri*
Benson et al. (2013a)	130. Height of cervical neural spines	(0) taller than their craniocaudad length; (1) longer than tall	(0) taller than their craniocaudad length
Benson & Druckenmiller (2014)	173. Cervical centra	(0) mediolateral width subequal to height or less; (1) at least 1.2 times as wide mediolaterally as high dorsoventrally	(0) mediolateral width subequal to height or less
	183. Dorsal neural spines with strong craniocaudad constriction at base	(0) absent; (1) present	(1) present

Three conflicting character states remain, which are listed in Table 4:

1. Height of cervical neural spines (character 130 of Benson et al., 2013a). In *G. wegneri* only six caudal cervical vertebrae are preserved. They bear neural spines that are taller relative to the length of the centra (character state 0). The same condition is present in the caudal cervicals of *B. brancai*, but in the cranial cervicals the neural spines are longer than tall (character state 1).
2. Proportions of cervical centra (character 173 of Benson & Druckenmiller, 2014). In most cervicals of *B. brancai* the width/height dimensions are not significantly different (character state 0, see Wegner, 1914: 258–259). In four of the last five cervicals (which are compatible with those preserved in *G. wegneri*), the width/height dimensions range between 1:1.08 and 1:1.15. Only in the terminal cervical vertebra, the dimensions are higher with 1:1.24 (character state 1). The width/height dimensions in the cervicals of *G. wegneri* are ranging between 1:1.10 and 1:1.17 (possibly even 1:1.20 in C4, see Hampe, 2013, Table 1). We consider this a minor difference well within the range of individual and ontogenetic variation.
3. Dorsal neural spines with strong craniocaudad constriction at base (character 183 of Benson & Druckenmiller, 2014). The dorsal neural spines of *B. brancai* bear constricted bases, but only on some vertebrae. We therefore scored this character as a polymorphism (character states 0/1). *G. wegneri* alternatively displays marked constrictions, and the neural spines are taller, a feature we attribute to relative degree of ossification (see Brown, 1981).

In summation, the differences between *B. brancai* and *G. wegneri* are minor and would not justify seperation at genus level; this is also supported by our phylogenetic analysis (see below). We therefore consider these states coherent with ontogenetic and/or intraspecific variation (e.g., Brown, 1981; Großmann, 2007), and conclude that *B. brancai* and *G. wegneri* are synonymous.

### Comparisons with other European “Wealden facies” plesiosaurians

Various other plesiosaurian specimens have been reported from the European “Wealden facies.” Koken (1887) and Koken (1896) provided the first descriptions of material from Lower Cretaceous strata of northwestern Germany and established two species, *Plesiosaurus limnophilus* and *Plesiosaurus degenhardti.*

*Plesiosaurus limnophilus* was based upon three isolated cervical centra found in the upper Bückeberg Group at Ummeln east of Hannover, and at Kniggenbrink hill near Barsinghausen in Lower Saxony. The type specimen from Ummeln (GPMM A3B.5) has recently been rediscovered and closely resembles the craniad cervicals of *B. brancai* in proportions. The concave articular faces also bear a notochordal pit and deep grooves next to the mid-ventral keel.

**Figure 24:**
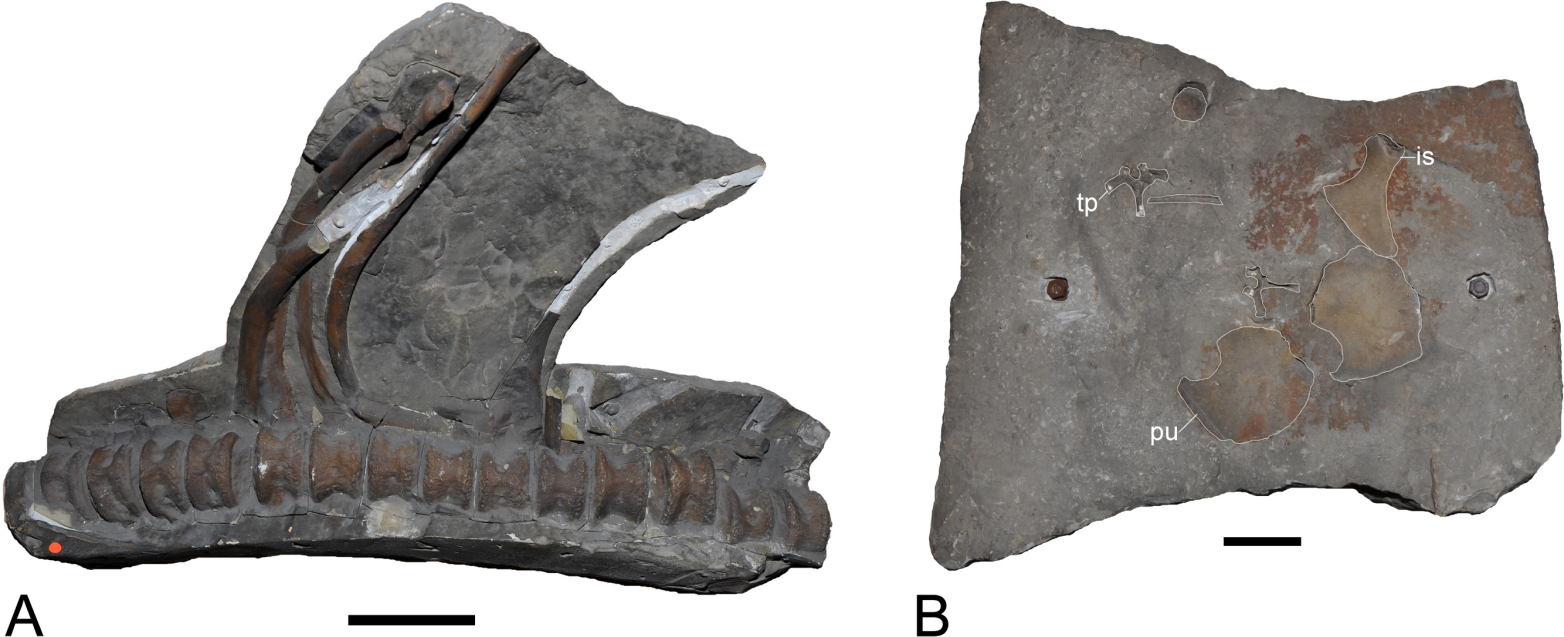
Plesiosaurian remains from the Obernkirchen Sandstone, Barsinghausen Member, Deister Formation, Bückeberg Group. (A) Plesiosauria indet, MB.R.1993.8–10 (plaster cast of holotype of *Plesiosaurus degenhardti* Koken, 1887), Obernkirchen, articulated dorsal vertebrae and ribs. (B) *Brancasaurus* sp. (GZG.BA.0079), Bückeburg area, associated postcranial elements, preserved as natural molds. Scale bars = 100 mm. Abbreviations: is, ischium; pu, pubis; tp, transverse process.

*Plesiosaurus degenhardti* was established by Koken (1887), based on 21 dorsal vertebrae and ribs (MB.R.1993.1-10) from the Obernkirchen Sandstone (Barsinghausen Member, Deister Formation, “Wealden 3”) of Obernkirchen in Lower Saxony. These were all preserved as natural moulds in a carbonate-depleted quartzose sandstone matrix (see Koken, 1887; Hornung, Böhme & Reich, 2012) with some historical plaster cast positives still preserved in the MB collection (Fig. 24A), although not all specimens mentioned by Koken were available for examination. Koken’s (1896) description also indicates that the type material of *P. degenhardti* shows no characters that would justify a referral to *B. brancai,* or are otherwise diagnostic to genus or family level. Later Koken (1896) assigned 23 articulated cervicals and pectorals (likewise stored in the MB collection) from the same locality and stratum to *P. degenhardti.* The centra have slightly rounded articular surface rims, but differ from *B. brancai* by the presence of double-headed cervical ribs and the absence of “shark-fin” shaped neural spines. *P. degenhardti* is thus best considered a *nomen dubium* as suggested by Welles (1962).

Koken (1905) ascribed an associated row of mid-dorsals and one sacral vertebra, rib fragments, two broken proximal femora and part of a jaw from the Isterberg Formation of the Gerdemann & Co. clay-pit to *P. degenhardti.* These apparently derived from a more complete skeleton that was destroyed prior to recovery (Koken, 1905). Wegner (1914: 297–299) considered the vertebrae to be distinct from *B. brancai* because they had shorter neural spines with shallow cavities on their edges. However, we consider these to be minor differences, which cannot be confirmed because the whereabouts of the original fossils are unknown.

GZG.BA.0079 comprises a sandstone slab from the Obernkirchen Sandstone of the Bückeburg area, Lower Saxony, with associated postcranial elements, including both pubes, an ischium, two detached dorsal neurapophyses, a centrum, and a rib fragment of a subadult plesiosaurian (Fig. 24B). This material shows a morphological overlap with *Brancasaurus brancai*, including an undulating cranial rim of the pubis, a pubic section of the pelvic bar that formed a conspicuous rhombic opening in the midsection of the pelvic bar, and subdiapophyseal fossae on the dorsal transverse processes. However, the latter seem to be more restricted to the proximal parts of the transverse processes in the Gronau material. Because of this difference and the incompleteness, GZG.BA.0079 is here referred to as *Brancasaurus* sp.

Plesiosaurian material from Jurassic–Cretaceous boundary strata of the Purbeck Limestone Group in southern England incorporate osteologically immature cervical centra (NHMUK R1607), which are morphologically similar to *B. brancai* (see Kear, Milner & Barrett, 2009: 123, Figs. 2A–2C). In contrast, the iconic taxon *Leptocleidus superstes* from the Barremian upper Weald Clay Formation of the Wealden Supergroup is clearly phylogenetically distinct (e.g., O’Keefe, 2001; Druckenmiller & Russell, 2008a; Ketchum & Benson, 2010; Kear & Barrett, 2011; Benson et al., 2013a; Benson & Druckenmiller, 2014). Kear & Barrett (2011) and Ketchum (2011) discussed NHMUK R609, the holotype (a partial skeleton) of *Cimoliasaurus valdensis* (miss-spelled “*Cimoliosaurus*” by Lydekker, 1889: see Kear, 2002) from the Valanginian Wadhurst Clay Formation, which Benson et al. (2013a) referred to a new genus, *Hastanectes*, based on its apparently unique interruption of the ventral ridge on the cervical centra by the subcentral foramina (which we cannot confirm in *B. brancai*), and craniad expansion of the ventral ridge to form a triangular platform. *Hastanectes valdensis* further manifested a diagnostic state combination: at least 20 cervical vertebrae; broadly spaced cervical prezygapophyses; cervical centra with a prominent ventral “lip” on the cranial articular surface (elsewhere considered characteristic of polycotylids: Sato & Storrs, 2000; Kear, 2005c); a narrow ventral midline ridge on the cervical centra; single-headed cervical rib facets; and a sigmoidal humerus (seemingly based on the referred specimen NHMUK R5264). All of these features, except for the apparently interrupted ventral ridge and broadly spaced prezygapophyses are evident in *B. brancai*, rendering generic distinction of *H. valdensis* uncertain. Potential referral of the “*Plesiosaurus valdensis* (Lydekker, 1889)” (*sensu* Koken, 1905) material from Gronau to *H. valdensis* likewise cannot be demonstrated.

Benson et al. (2013a) erected *Vectocleidus pastorum* from the late Barremian Vectis Formation based upon a partial postcranial skeleton with autapomorphic “dorsal neural spines that are craniocaudally short, and successive spines alternate[ing] between being transversely compressed, and being expanded to the right” (Benson et al., 2013a: 235). Benson et al. (2013a: 239) remarked that similar traits were conspicuous in *B. brancai*, but that the dimensionally greater length versus height of the caudal cervical centra served to segregate this taxon. Pointedly, however, only two disarticulated cervicals were recovered with the holotype (MIWG 1997.302) of *V. pastorum*, and the cervical proportions of *B. brancai* vary from slightly longer than high to shorter than high along the middle- caudal cervical column (the cervical centra of *G. wegneri* are also slightly shorter than high: Hampe, 2013: 481, Table 1). The lack of a definitive placement for the cervicals in *V. pastorum* therefore leads us to consider its diagnosis as inadequate. Moreover, we find that the purportedly longer dorsal neural spine apices (Benson et al., 2013a: 240) to be inconsistent. Given these observations, and the as yet unexplored potential for taphonomic, diagenetic or pathological (e.g., Voss, Asbach & Hilger, 2011) modifications, we treat the validity of *V. pastorum* as an open question and regard both *B. brancai* and *L. superstes* to be the only unequivocally definable plesiosaurian taxa currently known from the “Wealden facies” of Europe.

### Phylogenetic analysis

**Figure 25:**
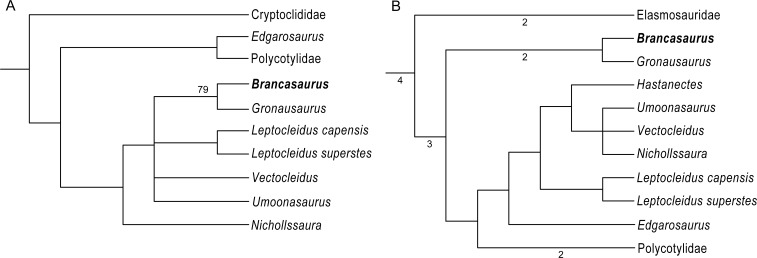
Alternative topological placements of *Brancasaurus brancai*. (A) Strict consensus of four most parsimonious trees (Length [L] = 20,663; CI = 0.3408; RCI = 0.2185) derived from the modified Benson et al. (2013a) dataset. (B) Strict consensus of >10,000 trees (= Maxtrees) recovered from parsimony analysis of the modified Benson & Druckenmiller (2014) dataset (L = 1,401; CI = 0.3445; RCI = 0.222). Bremer index (depicted where >0) and bootstrap values were calculated using parameters described in Ketchum & Benson (2010) and Benson & Druckenmiller (2014) respectively.

The holotype skeleton of *Brancasaurus brancai* (GPMM A3.B4) has sustained considerable damage and concomitant information loss since its initial description by Wegner (1914). Irrespectively, it still remains the most completely known European Early Cretaceous plesiosaurian, and one of the most historically famous plesiosaurian taxa documented worldwide. Enigmatically, however, its phylogenetic relationships are persistently conflicting. To explore this contention, we rescored the original fossils of *B. brancai* and its osteologically ‘adult’ synonym *Gronausaurus wegneri* into two recently published character datasets of Plesiosauria: Benson et al. (2013a), which was specifically compiled to accommodate for Wealden plesiosaurians; and Benson & Druckenmiller (2014), which returned deviating placements of *B. brancai* and *G. wegneri* within Leptocleididae versus Elasmosauridae respectively. We additionally modified scores for the Late Cretaceous elasmosaurid *Libonectes morgani* Welles, 1949 as advocated by Sachs & Kear (2015a). Our PAUP*4.0b10 (Macintosh: Swofford, 2002) searches of the Benson et al. (2013a) dataset used a default heuristic setting with TBR (tree-bisection-reconnection) branch swapping and zero length branches collapsed (‘amb-’ setting). All gap-weighted characters were treated as ordered while non-quantitative characters were unordered and weighted by 26 in accordance with the maximum number of states designated in the Ketchum & Benson (2010) parent matrix. *Cymatosaurus* was designated as the user-defined outgroup. The unordered characters from Benson & Druckenmiller (2014) were likewise analyzed using our default settings in PAUP*, with *Yunguisaurus liae* set as the outgroup taxon. Support measures (bootstrap/Bremer index) were calculated following parameters designated in Benson et al. (2013a), and Benson & Druckenmiller (2014) respectively. These analyses produced strict consensus topologies (Figs. 25A and 25B) that unanimously placed *B. brancai* + *G. wegneri* as sister taxa. In fact, these terminals differed by only a few polymorphisms comprising 0.5% (1/216) to 0.7% (2/270) of all available states (Table 4). *Brancasaurus brancai* + *G. wegneri* were alternately nested within Leptocleididae (Fig. 25A), or intercalated between Elasmosauridae and Leptocleididae + Polycotylidae (Fig. 25B). The arrangement of other taxa within these clades also contrasted with Benson et al. (2013a: 241, Fig. 5A) and Benson & Druckenmiller (2014: 8, Fig. 2), probably reflecting inherent homoplasy and/or missing information as suggested by previous evaluations (see Sachs & Kear, 2015a).

Given these results, we conclude that the higher-level classification of *B. brancai* cannot be unanimously resolved using current phylogenetic datasets. Furthermore, the various states advocating its alternating affinities are open to interpretation.

1. Substantially reduced rostral-most premaxillary aveoli. Benson et al. (2013a) coined this trait (apparently shared with *Leptocleidus capensis*: see Cruickshank, 1997: 219) from Wegner’s (1914: 243, Fig. 1C) drawings. The premaxillary alveoli are no longer preserved in GPMM A3.B4 thus their number and size cannot be discerned.
2. Maxilla-squamosal contact. Despite conflicting polarity (see O’Keefe, 2001; Sato, 2002), this character was used to exclude *B. brancai* from Elasmosauridae (see Sato, 2002). The left maxilla of GPMM A3.B4 does form a maxilla-squamosal contact (*sensu* Sato, 2002); however, Druckenmiller & Russell (2008a) considered the range of variation observed in other plesiosaurians to be too extreme to establish meaningful homology.
3. Elongate caudoventral process on the postorbital. This process is broken off in GPMM A3.B4 but was originally described by Wegner (1914: 249). Druckenmiller & Russell (2008a: 26) codified the postorbital in terms of its overlap with the squamosal; this was apparently substantial in *B. brancai*, unlike the leptocleidids *L. capensis* and *Nichollssaura borealis* in which the posterolateral process is prominent but “shares little, if any, contact with the squamosal”.
4. Dorsomedian foramen on the frontals. Sato (2002) nominated this feature to distinguish *B. brancai* from elasmosaurids. Druckenmiller & Russell (2008a) also identified a dorsomedian foramen in *L. capensis* and rhomaleosaurids (*sensu* Cruickshank, 1997); its expression in *N. borealis* was otherwise equivocal. Delimitation of the dorsomedian foramen is similarly controversial in other plesiosaurians (Smith & Dyke, 2008; Benson et al., 2013a) and the character is considered phylogenetically indeterminate (Druckenmiller & Russell, 2008a).
5. Triangular fossa tapering proximally from the pineal foramen to the merge with the sagittal crest. Benson et al. (2013a, Appendix S1: 2–3, character 37) cited the “parietal table” as uniquely shared by *B. brancai* and *N. borealis*. Its presence is otherwise uncertain in *Umoonasaurus demoscyllus* (Kear, Schroeder & Lee, 2006) and *Leptocleidus* spp. (Druckenmiller & Russell, 2008a; Kear & Barrett, 2011). Pointedly, our character transformations treat it as independently derived (see Benson et al., 2013a matrix scores, Appendix S1: 27), thus it does not intrinsically diagnose leptocleidid affinity.
6. Presence of a notch on the dorsal surface of the articular adjacent to the glenoid. Ketchum & Benson (2010) documented this structure in *B. brancai* as well as the polycotylids *Edgarosaurus muddi* Druckenmiller, 2002 and *Dolichorhynchops* Williston, 1902. Benson et al. (2013a) subsequently added *L. capensis* based on photographs (although a glenoid notch was not reported in the first-hand examinations of Cruickshank, 1997 or Druckenmiller & Russell, 2008a), and a comparable state was scored in *Plesiopleurodon wellesi* Carpenter, 1996 (Benson & Druckenmiller, 2014) and is likewise evident in a Pliensbachian plesiosaurian from Germany (Sachs, Schubert & Kear, 2014). Our phylogenies returned it as an unequivocal synapomorphy for Leptocleididae + Polycotylidae but it is not yet demonstrable in all constituent taxa.
7. Dorsoventrally broad trough on the lateral surface of the mandible. This trait likewise consistently discriminates Leptocleididae + Polycotylidae and is unequivocally manifest in *B. brancai*. However, it is also variously distributed throughout rhomaleosaurids and other plesiosaurians (Benson et al., 2013a; Benson & Druckenmiller, 2014) indicating widespread homoplasy.
8. Cervical neural spines curve caudodorsally. Relative curvature of the cervical neural spines changes along the reassembled column of GPMM A3.B4 (Benson et al., 2013a listed only the curved state). The caudal-most cervicals and pectorals of *G. wegneri* likewise differ in having rectangular neural spines (see Hampe, 2013: 476, Fig. 5A).
9. Caudal-most cervical neural spines with expanded suboval and concave dorsal apices. The cervcial–pectoral neural spines of GPMM A3.B4 are craniocaudally elongate (Benson et al., 2013a) and convex but become progressively concave along the reassembled column. The caudal-most cervical neural spines of the *G. wegneri* type remains are also transversely narrow and convex to flat in profile (Hampe, 2013: 476, Figs. 5A and 5B).
10. Dorsal neural apices with alternating asymmetrical morphology. This is identifiable in a few vertebrae of GPMM A3.B4 but many neural spines are remodelled, some are missing, and the column itself has been artificially reconstructed from disassociated components.
11. Dorsal neural spines subequal to height of the centrum. Contrary to Benson et al. (2013a) and Wegner (1914) clearly recorded neural spine heights in GPMM A3.B4 that exceeded those of the accompanying centrum. A comparable trend is observable in *G. wegneri* (Hampe, 2013).
12. Scapular shelf. Druckenmiller & Russell (2008a) listed the scapular shelf as a critical synapomorphy for Leptocleididae + Polycotylidae (= Leptocleidoidea *sensu* Druckenmiller & Russell, 2008a). Sato (2002), however, reported difficulty in differentiating the scapular ridge of elasmosaurids (e.g., *Hydrotherosaurus alexandrae* Welles, 1943: Welles, 1962). Kear & Barrett (2011) noted the presence of scapular ridges in other plesiosaurians (e.g., *Bishanopliosaurus youngi* Dong, 1980; Sato, Li & Wu, 2003; *Simolestes vorax* Andrews, 1909; Andrews, 1913; see also Evans, 2012; Sachs, Schubert & Kear, 2014), and this trait is known to be ontogenetically variable in basal sauropterygians (Sander, 1989; Sato, 2002).
13. Intercoracoid vacuity. The occurrence of this classic elasmosaurid synapomorphy (*sensu* Welles, 1962; O’Keefe, 2001; Sachs & Kear, 2015a) in *B. brancai* was inferred from Wegner’s (1914) idealized plaster restoration. However, based on Wegner (1914, plate 9, Fig. 1) the concave medial extremities of the coracoids suggest that some form of opening might indeed have been present. Kear & Barrett (2011: 674) similarly suggested that the intercoracoid “notch” of *Leptocleidus superstes* was interpretively ambiguous. The medial margin of the coracoid in *G. wegneri* is broken (Fig. 23D) and does not demonstrate the presence of an “intercoracoid embayment” (contra Hampe, 2013: 487).
14. Ventral midline projection on the coracoids. This is conspicuous in elamosaurids (Sato, 2002; Hiller & Mannering, 2005; Druckenmiller & Russell, 2006), and weakly expressed in both *B. brancai* and *G. wegneri.* However, disparate state distributions imply convergent derivation.
15. ‘S-curved’ humerus. The leading edge of the humerus in *Leptocleidus superstes*, *Hastanectes valdensis* and polycotylids is ‘sigmoidal’ or ‘S-curved’ in profile (see Albright III, Gillette & Titus, 2007). Benson et al. (2013a: 245) stated that “this character is difficult to assess as it is absent in *Nichollssaura* (Druckenmiller & Russell, 2008b),” and it is also present in some elasmosaurids (e.g., *Wapuskanectes betsynichollsae* Druckenmiller & Russell, 2006). We further indicate that it is relatively less pronounced in GPMM A3.B4 compared with *G. wegneri* and appears to vary with ontogeny (see Kear, 2007).
16. Proximodistally long epipodials. The epipodials of GPMM A3.B4 have been mislaid, but were widest mediolaterally (see Wegner, 1914: 286) in conflict with the scoring of Benson et al. (2013a). Hampe (2013) stated that the epipodials of *G. wegneri* were also proximodistally elongate but this is inconsistent (see Hampe, 2013: 488).

**Figure 26:**
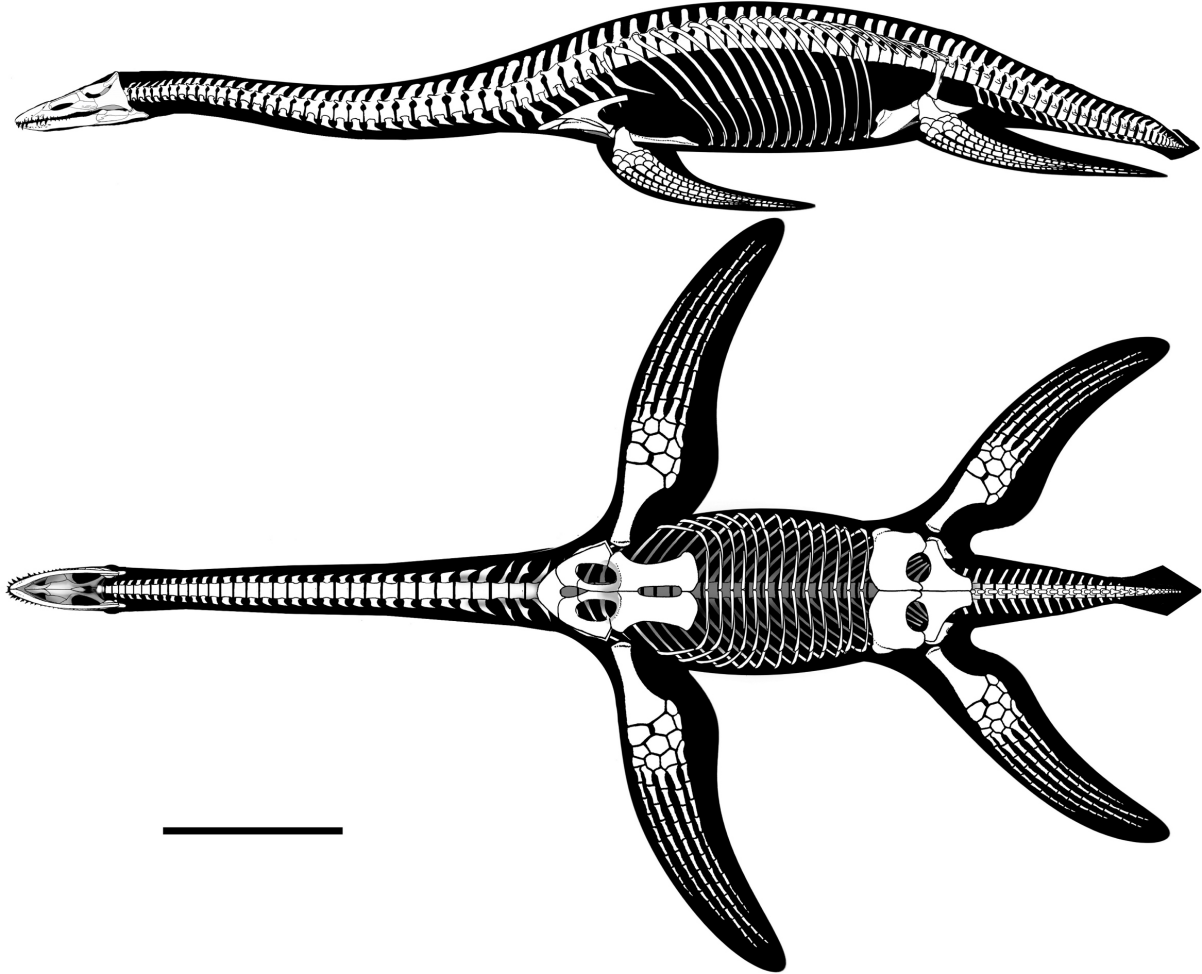
*Brancasaurus brancai*, skeletal reconstruction in lateral and ventral views based upon the preserved elements and the figures of Wegner (1914). The length of the pectoral paddle is hypothetical. Scale bar = 500 mm. Artwork by Jaime Headden.

**Figure 27:**
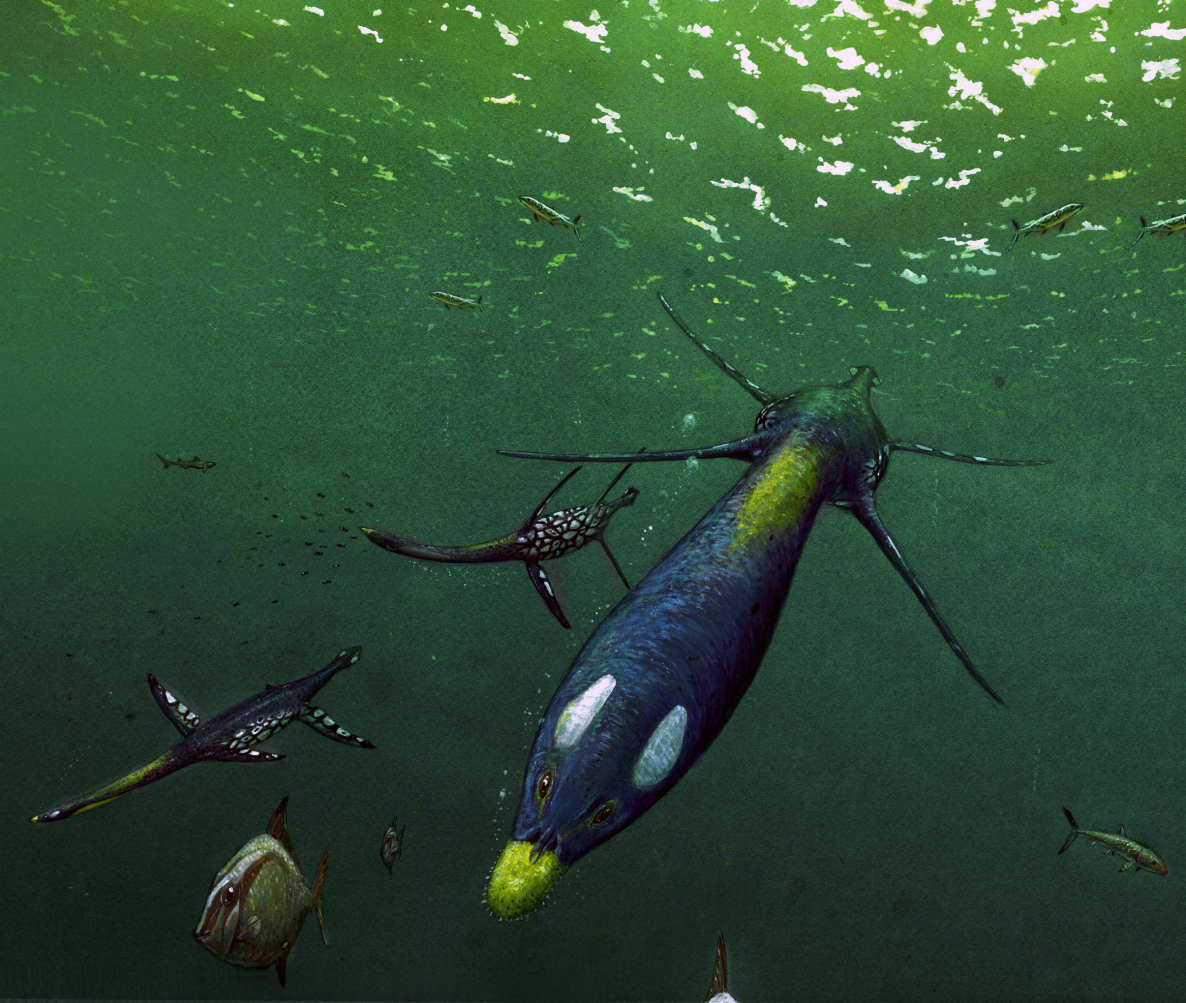
Life reconstruction of *Brancasaurus brancai* in its habitat. Artwork by Joschua Knüppe.

Benson & Druckenmiller (2014) ascribed the doubtful relationships of *B. brancai* to its mosaic of shared leptocleidid, polycotylid and elasmosaurid character states. Sato (2002) also emphasized that osteological immaturity potentially masked the phylogenetic signal of the holotype (GPMM A3.B4). We concur with both these denouements, and underscore that the ontogenetic changes observed in GPMM A3.B4 versus the ‘adult’ *G. wegneri* GPMM A3.B2 have a particularly significant impact on topological arrangements (see Phylogenetic Definition herein)—especially relative fusion of the basicranial components, and prominence of the basioccipital tubera, notochordal pit and condylar groove (see Brown, 1981; Maisch, 1998; Kear, 2007); vertebral count, centrum proportions, articular facet concavity and neural spine morphology (which vary widely along the column: Brown, 1981; O’Keefe & Hiller, 2006; Sachs, Kear & Everhart, 2013; Sachs & Kear, 2015a); and shape of the girdle elements together with ossification of the pectoral/pelvic bar and proportions of the propodials (Brown, 1981; Carpenter, 1999; Kear, 2007). We therefore recommend that the taxonomic affinities of *B. brancai* (Figs. 26 and 27) remain provisional until further data can better elucidate both character distributions and growth-related intraspecific change in what appears to be one of the most phylogenetically pivotal Cretaceous plesiosaurian taxa documented globally.

## Conclusions

The holotype specimen of *Brancasaurus brancai* from the uppermost strata of the Bückeberg Group (upper Berriasian) of northwestern Germany is one of the anatomically most complete Early Cretaceous plesiosaurian fossils known from Europe. Since its initial description in 1914, the specimen has suffered severe damage. Nonetheless a unique combination of diagnostic traits is present, including: rectangular conjoined frontals with a concave dorsal surface and ventrally confluent lateral sides; parietals forming a parietal table; cranial and middle cervicals with distinctly triangular neural spines; dorsal transverse processes bearing subdiapophyseal fossae; scapula with a prominent lateral shelf; pelvic bar formed by the pubes and ischia; and craniolateral cornua present at the pubes. Pointedly, the holotype specimen of *B. brancai* was ostologically immature, as indicated by the unfused neural arches and vertebral centra. However, other features (e.g., presence of cornuae on the pubes, and well defined epipodial facets on the propodials) indicate expression of at least ‘sub-adult’ character state development. Another but more incomplete plesiosaurian skeleton from the *B. brancai* type locality in the upper Bückeberg Group has been named *Gronausaurus wegneri*, but likely represents a more mature conspecific individual. Some variation is present in the number of dorsal/sacral vertebrae. Our phylogenies otherwise detected character state conflict only in the height of the cervical neural spines, proportions of the cervical centra, and basal constriction of the dorsal neural spines. Nevertheless, these constituted polymorphisms that probably reflect specimen completeness and/or differing ontogenetic stage, suggesting that *G. wegneri* represents a junior synonym of *B. brancai*. Finally, in our opinion, the weakly supported alternative topological nesting of *B. brancai* + *G. wegneri* either within Leptocleididae, or interpolated between Elasmosauridae and Leptocleididae + Polycotylidae dictates that the taxonomic affinities of *B. brancai* must, at present, remain provisional.

## Institutional abbreviations

DLM: Driland Museum, Gronau (Westfalen), Germany
GPMM: Geomuseum der Universität Münster, Münster in Westfalen, Germany
GZG: Geowissenschaftliches Zentrum der Georg-August-Universität Göttingen, Göttingen, Germany
MB: Museum für Naturkunde, Berlin, Germany
MTWE: Museum TwentseWelle, Enschede, The Netherlands
MIWG: ‘Dinosaur Isle’ Museum of Isle of Wight Geology, Sandown, UK
NHMUK: Natural History Museum, London, UK
SMF: Naturmuseum Senckenberg, Frankfurt am Main, Germany

## Appendix

Revised character scores (NEXUS format) for holotype specimens of *Brancasaurus brancai* (GPMM A3.B4) and *Gronausaurus wegneri* (GPMM A3.B2).

Benson et al. (2013a)

Brancasaurusbrancai_R

???0??????102??1??0?????????1?021?1?311?11110????????1?????????????000????????121???0??????? 10???1?????0?????????0010H1000(12)(01)20211(01)111081(01)1???0???1??????C9?11011?????? 1111016171??010110??0?(01)?110011?10?0???02???1(01)(01)0?10

Gronausauruswegneri_R

?????????????????????????????????????????1?????????????????????????0001???????121???0???????????????? ?????110?????????1?00(12)(01)??21101????1(01)???????????????C9?1101??81???11?1?1?171????? ??????????10????10?0?????????(01)(01)0110

Benson & Druckenmiller (2014)

Brancasaurus_brancai_R

00???0?0?0?????0102???0011??????0??????1?010??02??3?1?1?110?001101?(01)1???1???????0022?? ?????1???????????????????????100?1110???2??1??????????2010???10?(12)4100?0(01)232012?01011 10(01)0(02)1110011(01)0000(12)01?101????1??1??20???????210?0?010121021010??????0111? 02001022210????????????????0

Gronausaurus_wegneri_R

?????????????????????????????????????????????????????????1?????????????????????????????????????????????? ??????????????????????????????????????????????????01?(01)2?20?2101011??00?111001110000(12)01 1101???????????????????210???010121021010??????0111?02001022210???????0????????0

## Additional Information and Declarations

### Data Availability

The following information was supplied regarding data availability: The research in this article did not generate, collect or analyse any raw data or code.
